# Genetic architecture and mechanism of seed number per pod in rapeseed: elucidated through linkage and near-isogenic line analysis

**DOI:** 10.1038/srep24124

**Published:** 2016-04-12

**Authors:** Yuhua Yang, Jiaqin Shi, Xinfa Wang, Guihua Liu, Hanzhong Wang

**Affiliations:** 1 Oil Crops Research Institute of the Chinese Academy of Agricultural Sciences, Key Laboratory of Biology and Genetic Improvement of Oil Crops, Ministry of Agriculture, Wuhan 430062, China

## Abstract

Seed number per pod (SNPP) is one of the major yield components and breeding targets in rapeseed that shows great variation and is invaluable for genetic improvement. To elucidate the genetic architecture and uncover the mechanism of SNPP, we identified five quantitative trait loci (QTLs) using the BnaZNRIL population, which were integrated with those of previous studies by physical map to demonstrate a complex and relatively complete genetic architecture of SNPP. A major QTL, *qSN.A6*, was successfully fine-mapped from 1910 to 267 kb using near-isogenic line (NIL). In addition, *qSN.A6* exhibited an antagonistic pleiotropy on seed weight (SW), which is caused by a physiological interaction in which SNPP acts “upstream” of SW. Because the negative effect of *qSN.A6* on SW cannot fully counteract its positive effect on SNPP, it also enhanced the final yield (17.4%), indicating its great potential for utilization in breeding. The following genetic and cytological experiments further confirmed that the different rate of ovule abortion was responsible for the ~5 seed difference between Zhongshuang11 and NIL-*qSN.A6*. This systematic approach to dissecting the comprehensive genetic architecture of SNPP and characterizing the underlying mechanism has advanced the understanding of SNPP and will facilitate the development of high-yield cultivars.

Seed number per pod (SNPP) is one of three components (including pod number and seed weight) of yield and an important target trait of breeding in rapeseed (*Brassica napus L*.). The SNPP of rapeseed shows great variation in its germplasm resources (from 5 to 35 seeds per pod)^1^, which is invaluable for genetic improvement. As with other crops, SNPP is usually negatively correlated with the other two yield components^2^. These trade-offs are commonly explained as competition among sinks^3^,^4^. Genetically, the correlation between traits is generally considered to be either genetic linkage or pleiotropy^5^. Genetic linkage means the loci for different traits are physically near one another. Pleiotropy refers to the effect of a locus on two or more traits (Fig. 1). In addition, pleiotropy may be due to physiological interactions among traits in which one trait acts “upstream” of another^6^.

SNPP in rapeseed is a highly complex trait determined by the number of ovules per ovary, the proportion of fertile ovules (ovule fertility), the proportion of ovules fertilized, and the proportion of fertilized ovules that develop into seeds. In rapeseed, the number of ovules per ovary and ovule fertility are determined by the process of ovule differentiation and development^7^,^8^. The proportion of ovules fertilized is determined by the fertilization process, which is dependent on many factors such as pollen sterility, the amounts of pollen deposited on the stigma, pollen grain germination^9^, pollination conditions^10^, and pollen tube growth^11^. The proportion of fertilized ovules that develop into seeds is determined by the process of zygote/seed development. Although the main developmental and biological processes that determine SNPP are largely clear, which mechanism(s) might be responsible for the natural variation of SNPP among the different germplasms of rapeseed remains unknown. The limited knowledge of the mechanism of SNPP variation in rapeseed has hindered its improvement.

Understanding the genetic architecture of SNPP is the first step towards its improvement. During the past decade, nearly ten linkage mapping studies^2^,^12^,^13^,^14^,^15^,^16^,^17^,^18^,^19^,^20^ and one association analysis study^21^ involving the SNPP of rapeseed have been reported. However, the genetic architecture of SNPP has remained fragmented and the underlying candidate gene(s) have remained unknown because these detected QTLs had not been integrated and the physical map was unavailable. In addition, only one major QTL of SNPP has been fine-mapped with a relatively large physical region of more than 1 Mb^18^. More importantly, the mechanisms of these QTLs have not been investigated and thus remain unclear. Therefore, it is difficult to narrow down and identify the candidate gene(s) for these SNPP QTLs. NIL is a basic strategy commonly used for fine-mapping. The traditional NIL strategy can fine-map any mendelizing variation in theory.

The main objectives of the current study were as follows: (1) to dissect the genetic architecture of SNPP variation in rapeseed by linkage mapping as well as physical map-based integration with previous studies; (2) fine-map the major QTL using NILs; (3) to dissect the mechanism of SNPP variation at a major QTL through an exclusion strategy using a series of ingenious genetic and cytological experiments based on the NILs; (4) to dissect the genetic causation of the opposite effect of the major QTLs on SNPP and SW; and (5) to evaluate the application potential of the major QTL by field testing of its effect on yield as well as on yield components and related traits.

## Results

### Mapping of QTLs for SNPP using the BnaZNRIL population

The two parents, Zhongshuang11 and No. 73290, showed extremely significant differences in SNPP in all four investigated environments. The SNPP of Zhongshuang11 was 21.7 ± 1.6, which was approximately twice the SNPP of No. 73290 (11.7 ± 1.4) (Table 1). The SNPP of the RIL population showed more or less normal distributions in all four investigated environments (Figure S1), indicating a quantitative inheritance suitable for QTL identification. In addition, the SNPP of the RIL population exhibited transgressive segregation but to a small degree, indicating that the favourable alleles were mainly distributed in one of the two parents. The broad-sense heritability (*h*^2^ = 81%) of SNPP in the RIL population was relatively high.

**Figure 2:**
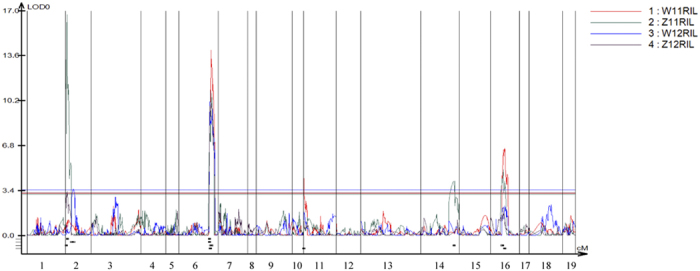
SNPP QTL scanning curves for the 19 linkage groups in four environments. The horizontal and vertical axes represent the genetic distance and LOD value, respectively. The lines and curves indicate the threshold and true LOD values, respectively. The different environments are represented using different colours, as indicated in the legend. W11, W12, Z11, and Z12 are the codes for the environments detailed in Materials and Methods.

The RIL genetic map consisted of 19 linkage groups and 2264 unique loci/bins (Table S1), which covered a total length of 2107 cM, with an average distance of 0.93 cM. Eight significant QTLs and three suggestive QTLs were detected for the SNPP. After deleting two non-reproducible suggestive QTLs, nine QTLs were identified (Fig. 2), which were distributed on the A02, A06, C01, C04, and C06 linkage groups (Table S2A). After the interaction of overlapping identified QTLs in different environments (Table S2B), five consensus QTLs were obtained, which explained 5.3–24.8% of phenotypic variance (Table 2). Of the two repeatable consensus QTLs, *qSN.A6* (defined as an 18.9 cM region between the markers Bn-A06-p22608192 and Bn-A06-p24274213) was consistently detected in all four environments and showed a relatively large effect (mean *R*^2^ = 20.1%; mean additive effect = 1.58), and thus it could be treated as a major QTL for further dissection.

### In silico integration of QTLs for SNPP in *Brassica napus*

**Figure 3:**
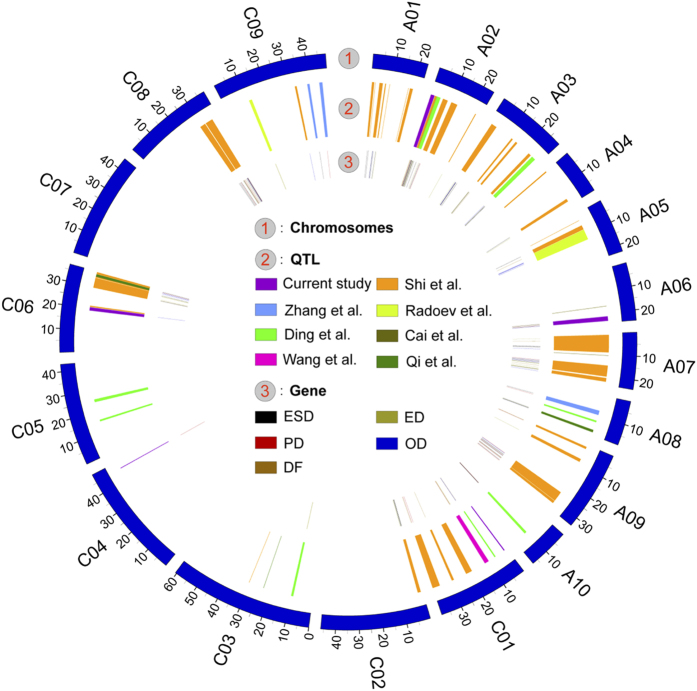
Integration of SNPP QTLs and candidate genes for on the reference genome of Brassica napus. Concentric circles show the different features that were drawn using the Circos program. 1, 19 chromosomes of rapeseed. 2, Consensus QTLs of SNPP, different colours represent different studies. 3, Genes referred to QTL mapping results for SNPP, ESD (embryo sac development), ED (embryo development), PD (pollen development), OD (ovule development), and DF (double fertilization), which are represented by different colours. QTL mapping range and genes are represented by squares and lines, respectively.

Of the total of 100 QTLs and seven association signals identified in the current and reported QTL mapping and association mapping studies (Table S3), 81 QTLs and four association signals were successfully anchored to the physical map, which were then subjected to meta-analysis, resulting in 70 consensus QTLs (57 non-overlapping QTLs and 13 overlapping QTLs) (Fig. 3). These consensus QTLs were distributed on almost all of the 19 chromosomes (except for C07). The R^2^ of these consensus QTLs ranged from 2.3 to 22.5. Most (70%) showed only small effects (R^2^ < 10%), and only five should be considered as major QTLs.

A total of 306 genes (Table S4) (for the main biological processes affecting SNPP) collected from Arabidopsis were aligned to 855 loci on the genome of Darmor by BLAST. Of these, 117 genes were found to underlie the corresponding 155 loci genomic regions of 41 consensus QTLs, which therefore should be considered as candidates. These candidates involve embryo sac development (ESD), ovule development (OD), pollen development (PD), double fertilization process (DF) and embryo development (ED).

### Fine-mapping of *qSN.A6* using BC_4_F_2_ population and BC_4_F_3_ progeny

The QTL-NILs of BC_4_F_1_ were obtained by the successive backcrossing of F_1_ with Zhongshuang11. During the backcrossing process, the progenies of each backcross were selected using two flanking SSR/InDel markers (BrSF47–389 and ni108) of the target region, which were very close to Bn-A06-p22608192 and Bn-A06-p24274213, respectively. For the BC_4_F_1_ lines, 111 plants were surveyed using not only two flanking markers for the selected foreground but also a total of 80 SSR/SNP markers that were evenly distributed on the 19 linkage groups to screen the genetic background (Table S5). The background proportions of these plants ranged from 87.3% to 97.6%, and several individuals with > 95% were self-crossed to produce BC_4_F_2_.

**Figure 4:**
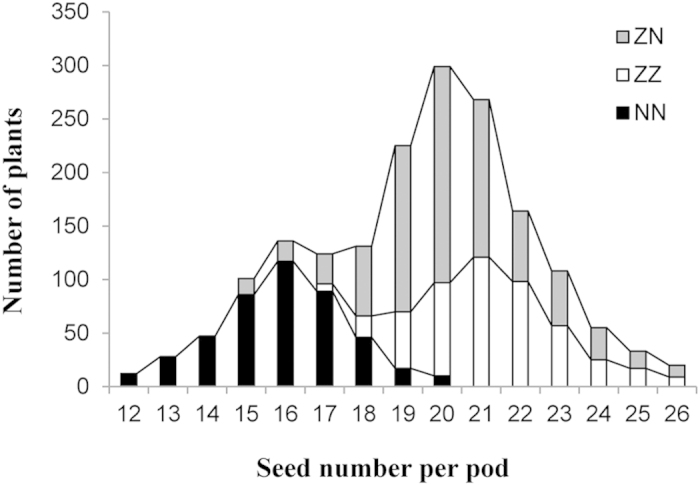
Frequency distribution of SNPP in the BC_4_F_2_ population. The horizontal axis represents the trait value of SNPP. The vertical axis represents the number of individuals. The three types of genotype are represented by the three column colours, as shown in the legend.

As expected, the frequency distribution of SNPP of the BC_4_F_2_ population deviated from the normal distribution (D = 0.16, *p* < 0.01) and appeared to be a bimodal distribution (Fig. 4). The ratio of the two phenotypic types was a good fit to the expected ratio of 1:3 (χ^2^ = 3.51, *p* = 5.6E–1), which indicated a single-locus Mendelian segregation. In the BC_4_F_2_ population, the numbers of three types of QTL genotypes (494:886:457), i.e., homozygous for the Zhongshuang11 allele (ZZ), heterozygous (ZN) and homozygous for the No. 73290 allele (NN), showed an expected ratio of 1:2:1 (χ^2^ = 3.79, *p* = 1.5E–1), indicating an absence of distorted segregation. Clearly, the SNPP of the BC_4_F_2_ plants with the ZZ genotype (21.6 ± 1.4) was slightly higher (*p* = 3.2E–2) than the SNPP of the heterozygotes (20.6 ± 1.6), and both were much higher (*p* = 1.1E–18 and 1.0E–15) than for the NN genotype (16.0 ± 0.4). These results suggested that the favourable allele of *qSN.A6* was from Zhongshuang11 and the mode-of-inheritance of *qSN.A6* was partially dominant. As expected, in the BC_4_F_2_ population, SNPP was significantly positively correlated with seed yield per plant (SY) (r = 0.51; *p* < 0.0001). Interestingly, SNPP was also significantly negatively correlated with seed weight (SW) (r = −0.30; *p* < 0.0001) but not significantly correlated with pod number (PN) (r = 0.10; *p* = 0.1587).

**Figure 5:**
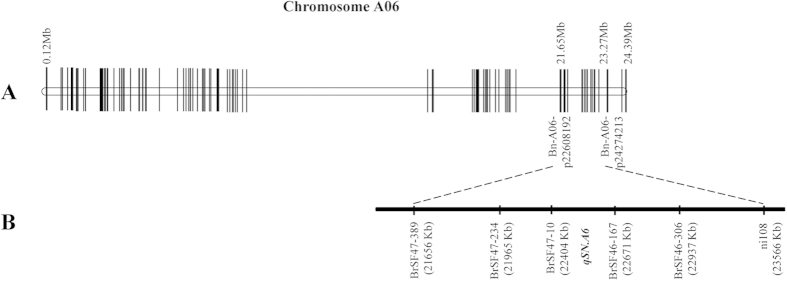
Fine-mapping of *qSN.A6*. (**A**) Position of *qSN.A6* on the A6 linkage group. The hollow vertical column shows the linkage group in which the position of each SNP marker is indicated by horizontal short lines. (**B**) The location of fine-mapped *qSN.A6*. The vertical solid columns show the physical map of *qSN.A6*, on which the physical position of each SSR/InDel marker is indicated with short lines.

Six evenly distributed co-dominant SSR/InDel markers within the 18.9 cM interval of *qSN.A6* (between SNP markers Bn-A06-p22608192 and Bn-A06-p24274213) were used to genotype the 2586 plants of the BC_4_F_2_ population (Fig. 5). A total of 20 individuals belonging to ten possible types of recombination were identified. Based on progeny testing of these lines, the ten types of recombination were classified into two groups based on the comparison of their SNPP with the recurrent parent Zhongshuang11 (21.2 ± 1.3) (Table 3). The first group included four types (type 1, 2, 9, and 10) whose SNPP (21.2 ± 1.7 to 21.8 ± 1.0) were not significantly different from Zhongshuang11. The SNPP (15.9 ± 0.8 to 16.7 ± 2.2) of the other group (including type 3, 4, 5, 6, 7, and 8) was significantly lower than for Zhongshuang11. All six types of recombination have a common introgression fragment between markers BrSF47–10 and BrSF46–167, which delimited *qSN.A6* to this interval.

### Dissection of the cytological mechanism of *qSN.A6* by the exclusion method

**Figure 6:**
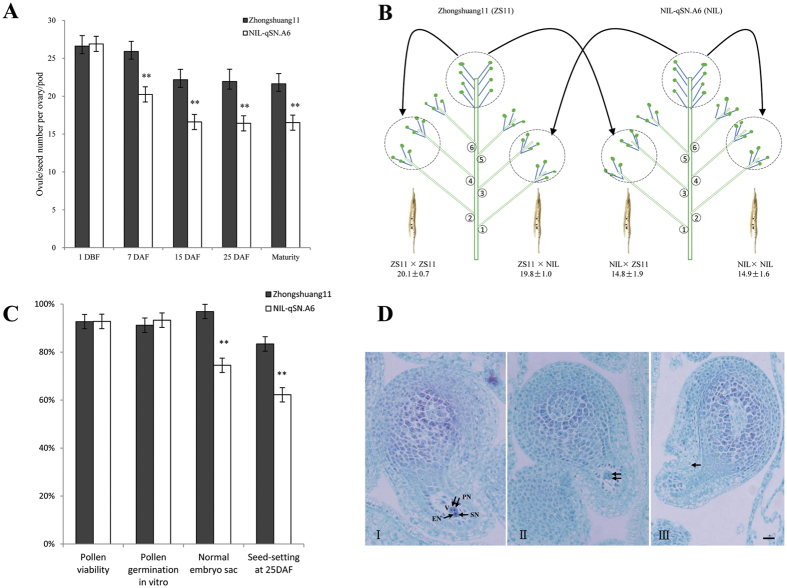
The cytological mechanism of *qSN.A6*. (**A**) Continuous observations of ovule/seed number per ovary/pod in Zhongshuang11 and NIL-*qSN.A6* from budding to maturity. The horizontal and vertical axes show the stages and number of ovules/seeds, respectively. The two lines, Zhongshuang11 and NIL-*qSN.A6*, are represented by black and white, respectively. The bars show the standard deviation of each value. DBF and DAF stand for days before flowering and days after flowering, respectively. ^**^represents a significance level of *P* < 0.01. (**B**) The SNPP of self- and cross-pollinations between Zhongshuang11 and NIL-*qSN.A6*. (**C**) The fertility-related traits of Zhongshuang11 and NIL-*qSN.A6*. (**D**) Normal embryo sac (I) and two types of abnormal embryo sac (II and III). A normal embryo sac consists of seven cells including eight nuclei: a single egg nucleus (EN), two polar nuclei (PN), two synergid nuclei (SN), and a central vacuole (V). The antipodal cells are degenerated or not clearly observed. The abnormal embryo sac exhibited two types of incomplete cellularization: (II) only two large cells were present in the embryo sac; (III) the embryo sac had not differentiated and lacked any visible cells. *Bar* equals 10 μm.

To determine the stage in which the SNPP difference formed between Zhongshuang11 and NIL-*qSN.A6*, continuous observations of ovule/seed number per ovary/pod from bud to maturity were conducted. At 1 DBF (day before flowering), the ovule number per ovary of Zhongshuang11 (26.6 ± 1.4) and NIL-*qSN.A6* (26.9 ± 1.4) did not differ significantly (*p* = 4.8E–1) (Fig. 6A). From 1 DBF to 7 DAF (day after flowering), the SNPP of both Zhongshang11 and NIL-*qSN.A6* decreased quickly, but the decrease speed of NIL-*qSN.A6* was faster than for Zhongshang11, and thus the SNPP of NIL-*qSN.A6* (20.2 ± 1.3) was significantly (*p* = 2.1E–13) lower than for Zhongshuang11 (25.9 ± 1.3). From 7 to 15 DAF, the SNPP of both Zhongshuang11 and NIL-*qSN.A6* decreased faster, with almost the same speed (Zhongshuang11: 22.2 ± 1.3; NIL-*qSN.A6*: 16.6 ± 2.3, *p* = 8.7E–15), and thus the difference between the two lines did not change from the previous stage (*p* = 8.8E–1). From 15 DAF to 25 DAF and to the maturity stage, the SNPP of both Zhongshuang11 and NIL-*qSN.A6* reached a stable period. These results implied that the SNPP difference between Zhongshang11 and NIL-*qSN.A6* forms before 7 DAF. From 1 DBF to 7 DAF, many factors affect the seed setting rate, such as pollen fertility, ovule fertility, and the fertilization process (pollen tube elongation). To identify the key factor responsible for the seed setting rate difference between Zhongshang11 and NIL-*qSN.A6*, the following genetic and cytological experiments were performed.

First, to identify the male or female origin of the SNPP difference between Zhongshang11 and NIL-*qSN.A6*, self- and cross-pollinations were performed by hand using the two lines (Fig. 6B). The results showed that, whether Zhongshang11 or NIL-*qSN.A6* was used as the mother plant, the SNPP of the self and cross-pollinated pods was not significantly different. These results strongly suggested that the seed setting rate difference of Zhongshang11 and NIL-*qSN.A6* was not related to pollen. To confirm this result, the main characteristics (including pollen viability, germination efficiency, and adherence to stigma) (Fig. 6C) reflecting the quality and quantity of pollen for both lines were checked, and no significant differences were found (Figure S2A–F). Second, to determine whether the seed setting rate difference between Zhongshuang11 and NIL-*qSN.A6* was related to the fertilization, the process of pollen tube elongation (Figure S2G,H) was observed, and no significant difference was found.

The results of the above experiments strongly indicated that the SNPP difference between Zhongshuang11 and NIL-*qSN.A6* should be related to the quality of ovules. Therefore, we conducted serial sectioning to investigate embryo sac fertility. As expected, the embryo sac fertility of NIL-*qSN.A6* was only 74.5 ± 4.5%, which was significantly (*p* = 9.0E–15) lower than for Zhongshang11 (96.9 ± 3.1%), which accurately explained the ~5 seeds per pod difference between Zhongshuang11 and NIL-*qSN.A6* (Fig. 6C). Generally, the fertile embryo sac contains seven cells including eight nuclei (Fig. 6D(I)). However, the cellularization of the embryo sac was incomplete, with one of the following two scenarios: (Fig. 6D(II)) only two large cells were present in the embryo sac or (Fig. 6D(III)) the embryo sac had not differentiated and lacked any visible cells. These results showed that the cytological mechanism of *qSN.A6* was due to ovule abortion caused by incomplete cellularization of the embryo sac.

### Effect of *qSN.A6* on yield and other important traits

In the large-scale field test at Wuhan in 2014–2015, the SNPP of Zhongshuang11 (21.2 ± 0.5) was significantly (*p* = 6.3E–10) higher (percentage = 25.1%) than in NIL-*qSN.A6* (15.9 ± 1.2) (Table 4). Regarding the other two yield component traits, the SW of Zhongshuang11 (4.29 ± 0.06) was significantly (*p* = 8.4E–07) lower (percentage = 9.1%) than in NIL-*qSN.A6* (4.68 ± 0.24), but the PN of the two lines did not differ. Strikingly, the SY of Zhongshuang11 was significantly (*p* = 4.7E–02) higher (percentage = 17.4%) than in NIL-*qSN.A6*. The other traits related to yield did not significantly differ between the two lines. In conclusion, *qSN.A6* exerts significant opposite effects on two of the three yield components but not on PN; however, the decrease in SW cannot counteract the increase in SNPP (which is also reflected by the moderately negative correlation between the two traits in the BC_4_F_2_ population), and the final seed yield is increased.

To further investigate the genetic causation of the effects of *qSN.A6* on both SNPP and SW, conditional QTL analysis was performed on the RIL population. Interestingly, when SNPP was conditioned by SW, the LOD value and R^2^ of *qSN.A6* showed small variation and remained significant (Table 5). However, the effect of *qSN.A6* became non-significant when SW was conditioned by SNPP (SW|SNPP). These results supported that the causation of the genetic effect of *qSN.A6* on both SNPP and SW was pleiotropy (rather than tight linkage) through a physiological interaction in which SNPP acts “upstream” of SW.

## Discussion

### The complexity of the genetic basis of the SNPP natural variation in rapeseed

By integrating our detected QTLs with the previous studies based on the recently completed physical map of rapeseed, we obtain a complex genetic architecture of SNPP that includes several major QTLs and numerous small-effect QTLs with underlying candidate genes. This result is understandable because SNPP is the final consequence of many developmental/biological processes, each of which is controlled by many genes. To our knowledge, this is the first comprehensive genetic framework of SNPP, which will deepen our understanding of this trait and provide a blueprint for the genetic improvement of SNPP.

To obtain a high-resolution genetic basis of SNPP variation in rapeseed, fine-mapping of the identified major QTL *qSN.A6* was performed using the NIL strategy from a preliminary interval of 1910 to 267 kb (Fig. 5). The mendelizing of *qSN.A6* in the BC_4_F_2_ population enabled an accurate estimate of its mode-of-inheritance. Similarly to the dominant inheritance of *qSS.C9*^18^, *qSN.A6* showed a partial dominant inheritance. These results supported the hypothesis of dominance rather than over-dominance as the genetic basis of the SNPP heterosis in rapeseed, which suggested the potential of *qSN.A6* and *qSS.C9* in the utilization of heterosis.

### The cytological mechanism of SNPP variation first uncovered via the systematic investigation of *qSN.A6*

Although the developmental processes (such as the fertility of ovule and pollen) that affect the SNPP of rapeseed were relatively clear^8^,^22^, which process is responsible for the natural variation of SNPP was unknown. The mendelizing of *qSN.A6* provides a unique opportunity to investigate the mechanism of SNPP variation at a single-locus level. A series of well-designed experiments were performed to exclude the abovementioned processes one by one, and finally, the different rate of ovule abortion (due to incomplete cellularization of the embryo sac) was found to be responsible for the ~5 seed difference between Zhongshuang11 and NIL-*qSN.A6*. To our knowledge, this report is the first to address the mechanism responsible for SNPP variation in rapeseed. The demonstration of the detailed cellular mechanism of *qSN.A6* also enhanced the accuracy of the identification of its candidate gene(s).

The incomplete cellularization of the embryo sac is commonly observed in other plants, such as *Arabidopsis*, rice, and maize^23^,^24^,^25^,^26^. Further research showed that abnormal meiosis of the megasporocyte or abnormal mitosis of the functional megasporocyte is the main reason^27^,^28^. Based on two detailed structures (Fig. 6D (II and III)), the incomplete cellularization of the embryo sac might be the product of a single or lacking division of the megaspore mother cell. Therefore, the incomplete cellularization of the embryo sac of *qSN.A6* observed in our study is likely caused by abnormal meiosis of the megasporocyte.

### The genetic causation of trade-off (antagonistic pleiotropy) among sink traits

Source limitation forces an organism to allocate energy to processes in a competitive manner^29^. In seed plants, major trade-offs among “sink” traits (such as the number and size of seeds) are commonly observed^30^,^31^, as reflected by typical negative correlations. For rapeseed, negative correlations among the main sink traits, i.e., the three yield components, PN, SNPP, and SW, are also commonly present^17^,^18^. However, the genetic causation of the trade-off among these sink traits has not been previously investigated and remains unknown.

The opposite effects of *qSN.A6* on SNPP and SW provide an ideal example to investigate the genetic causation of the trade-off among sink traits. The results of conditional QTL analysis showed that it was caused by antagonistic pleiotropy rather than tight linkage. The detailed results of reciprocal conditional QTL analysis further revealed that this type of antagonistic pleiotropy was due to a physiological interaction in which SNPP acts “upstream” of SW, which is highly consistent with the commonly accepted physiological mechanism of negative feedback among sinks. To our knowledge, this study is the first that clearly demonstrates the genetic causation of trade-off among sink/yield-component traits in rapeseed as well as in other crops. Regarding the three main sink traits of rapeseed, in addition to SNPP, *qSN.A6* has a pleiotropic effect on SW but not on PN. This finding is understandable because (1) the determination stage of PN is earlier than SN, and both are earlier than SW and (2) after flowering, the pod wall (rather than the leaf) is the main “source” organ responsible for the size of its “sink”, which is determined by the number and size of the seeds.

### Utilization potential of *qSN.A6* in rapeseed breeding

The negative/decreasing effect of *qSN.A6* on seed weight cannot fully counteract its positive/increasing effect on SNPP, which is consistent with the coefficients of negative correlation between SNPP and SW in the BC_4_F_2_ population. In fact, the coefficients of negative correlation among the three yield component traits were also moderate in all previous studies. As expected, *qSN.A6* increased the final seed yield by a proportion of 17.4%, which indicated the great potential of the breeding utilization of *qSN.A6* in rapeseed.

## Methods

### Plant materials, field trial, and trait measurement

The F_7_ generation recombinant inbred line (BnaZNRIL) population of 184 families was derived by single-seed descent from a cross between two sequenced rapeseed cultivars, Zhongshuang11 (high seed number) and No. 73290 (low seed number). The near-isogenic lines (NILs) of the BC_4_F_1_ generation were developed by successive backcrossing of F_1_ with Zhongshuang11 as the recurrent parent four times (Figure S3). The BC_4_F_3_ progeny of the recombinant and reference lines were phenotyped to determine the *qSN.A6* genotypes of these lines and to further fine-map the target region.

The BnaZNRIL population was arranged in a randomized complete block design with two replications. Each block contained 3 rows with a spacing of 33.3 cm between rows and 16.7 cm between individual plants. It was planted in Wuhan and Zhengzhou from Oct. 2010 to May 2011 and from Oct. 2011 to May 2012 (code W11RIL, W12RIL, Z11RIL and Z12RIL). All populations and the two parents were sown by hand with 15 plants per row, and the field management followed standard agriculture practice. At maturity, ten representative individuals in the middle of the second row of each block were harvested.

Harvested plants were air-dried and stored at room temperature for approximately two weeks before testing. SNPP was calculated as the average number of well-filled seeds from a whole plant’s well-developed pods. The other traits were tested according to previous studies^2^,^32^.

### SSR/InDel and SNP genotyping

The SSR/InDel markers used to genotype the BC_4_F_2_ were previously developed by our laboratory^32^,^33^,^34^. The PCR procedure, electrophoresis, and silver staining were performed as previously described^34^. The *Brassica* 60 K Illumina^®^ Infinium SNP array, which was recently developed by the international *Brassica* Illumina SNP consortium, was used to genotype the BnaZNRIL population. The array hybridization and data processing was conducted by Emei Tongde Co. (Beijing) according to the manufacturer’s protocol (http://www.illumina.com/technology/infinium_hd_assay.ilmn). Leaf tissue was collected from seedlings of the BnaZNRIL, BC_1_F_1_, BC_2_F_1_, BC_3_F_1_, BC_4_F_2_ and BC_4_F_3_ populations. Genomic DNA was extracted according to the CTAB method^35^.

### Map construction and QTL mapping and integration

The genetic linkage map was constructed using the JoinMap 4.0 software (http://www.kyazma.nl/index.php/mc.JoinMap) with a threshold for goodness-of-fit of ≦ 5, a recombination frequency of < 0.4, and a minimum logarithm of odds (LOD) score of 2.0. All genetic distances were expressed in centimorgans (cM), as derived by the Kosambi function.

The linkage mapping of QTL was performed by composite interval mapping^36^ using the WinQTL Cartographer 2.5 software (http://statgen.ncsu.edu/qtlcart/WQTLCart.htm). The experiment-wise LOD threshold was determined by permutation analysis^37^ with 1,000 repetitions. LOD scores corresponding to *p* = 0.05 were used to identify significant QTLs, and *p* = 0.5 was used to identify suggestive QTLs. The overlapping suggestive QTLs and all significant QTLs were admitted and termed identified QTLs^2^. QTL meta-analysis was used to estimate the number and positions of the meta-QTLs, which were repeatedly detected in different environments and populations located on the same chromosomal region^38^. The computation was conducted according to the methods of a previous study^2^,^32^. The availability of the pseudochromosomes of *B. napus* enabled the physical map-based comparison of the detected QTLs. The corresponding genomic intervals of these QTLs were determined by BLAST/e-PCR analysis using the associated markers (within confidence intervals) with available probe/primer sequences against the physical map of *B. napus*. A total of 306 genes of *Arabidopsis* with known functions relating to the number of fertile ovules per ovary, the number of ovules fertilized, and the number of fertilized ovules developing into seeds in this study were collected from the TAIR website (http://www.arabidopsis.org/).

To dissect the genetic basis (pleiotropy or tight linkage) of the co-localization of the SNPP and SW QTLs, conditional analysis was performed as described previously^32^. The conditional phenotypic values y (T1|T2) were obtained by the mixed model approach for the conditional analysis of quantitative traits using QGAStation 1.0 (http://ibi.zju.edu.cn/software/qga/index.htm), where T1|T2 indicates that trait 1 is conditioned by trait 2. Then, conditional QTL mapping was conducted using the conditional phenotypic values as in the unconditional QTL mapping.

### Observation of ovule/seed number per ovary/pod in Zhongshuang11 and NIL-*qSN.A6*

The observation was conducted in five stages, i.e., one day before flowering (DBF), seven, 15, and 25 days after flowering (DAF), and at maturity. The ovule/seed number per ovary/pod was measured as the average number of normal ovules/seeds of three ovaries/pods sampled from ten individuals. To maintain the developmental identity of the samples, flowers from the same day and in the middle of the main inflorescence from individuals with the same flowering time were marked (with red rope) for sampling. At every stage, samples of three ovaries/pods in every plant were taken from at least 30 plants.

### Identification of the female and/or male origin of the *qSN.A6* effect

To identify the female and/or male origin of the *qSN.A6* effect, an ingenious genetic-mating experiment was designed. The alternate branches on the same mother plant of Zhongshuang11/NIL-*qSN.A6* were hand-pollinated using its own pollen and that of NIL-*qSN.A6*/Zhongshuang11, respectively. Each self/out-cross was repeated three times, and pollinations were completed within one day. At maturity, the hand-pollinated pods were harvested and threshed to measure the SNPP. Then a multiple comparison of SNPP was conducted among the hand-pollinated pods of Zhongshuang11, NIL-*qSN.A6*, and the reciprocal cross.

### Evaluation of pollen vitality, pollen germination efficiency, and pollen tube growth as well as embryo sac fertility

To test pollen vitality, pollen of Zhongshuang11 and NIL-*qSN.A6* was collected from recently completely opened flowers and stained with 1% acetocarmine^39^. Then, the cytoplasm integrity of these stained pollen grains was checked individually under a light fluorescence microscope (IX-71; Olympus, Tokyo, Japan), according to their staining and shape.

To investigate *in vitro* pollen germination efficiency, pollen of Zhongshuang11 and NIL-*qSN.A6* was collected and shaken off to spread evenly on a PGM (10% sucrose, 0.005% H_3_BO_3_, 10 mM CaCl_2_, 0.05 mM KH_2_PO_4_, 6% PEG 4000) glass slide with 0.3% agar and was then left at room temperature for 3 h, as described previously^9^. To observe *in vivo* pollen germination efficiencies and the path of pollen tubes inside the pistil, pistils at one and three DAF were cut (ovary wall was removed) and fixed in 50% FAA (50% ethanol, 5% glacial acetic acid, 3.7% formaldehyde, v/v) for 24 h, softened in 8 M NaOH at 65 °C for 3 h, washed with 50 mM K-phosphate buffer (pH 7.5), and stained in 0.1% aniline blue. The stained pistils and pollen tubes were observed using a light fluorescence microscope (IX-71; Olympus, Tokyo, Japan) under UV light^11^.

To investigate the fertility of the embryo sac before flowering, paraffin sections were prepared as described by Li *et al.*^40^. The buds of one DBF were sampled and fixed in 50% FAA solution, dehydrated through an ethanol series of 75%, 85%, 95% and 100% (v/v), embedded in paraffin wax according to the method of a previous study^41^, and observed using a light fluorescence microscope (IX-71; Olympus, Tokyo, Japan).

### Statistical analysis

The broad-sense heritability was calculated as h^2^ = 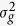/
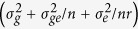
, as described previously, where 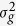, 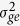, and 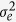 are the variance of genotype, genotype × environment, and error, respectively, and n and r are the number of environments and replications, respectively. The values of 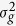, 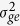, and 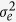 were estimated using the SAS ANOVA procedure. Pearson’s correlation coefficients, the Kolmogorov-Smirnov test, Duncan’s t-test, and multiple comparisons were performed using SAS software version 8.1.

## Additional Information

**How to cite this article**: Yang, Y. *et al.* Genetic architecture and mechanism of seed number per pod in rapeseed: elucidated through linkage and near-isogenic line analysis. *Sci. Rep.* **6**, 24124; doi: 10.1038/srep24124 (2016).

## Supplementary Material

Supplementary Information

## Figures and Tables

**Figure 1:**
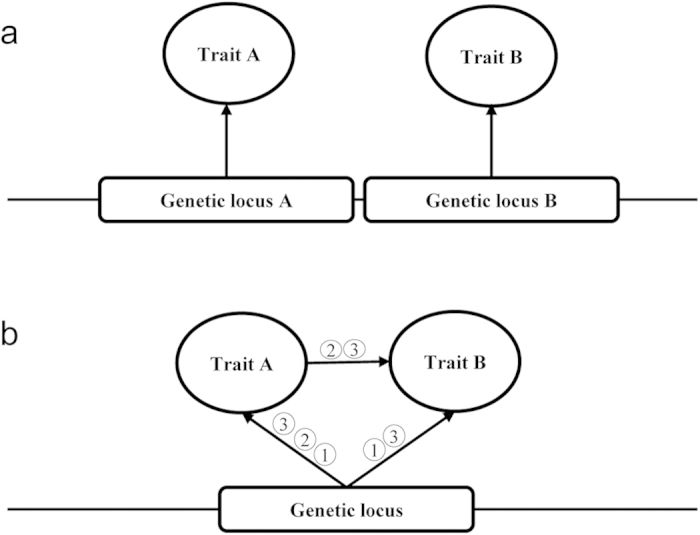
Diagram of the genetic causation of correlation among traits. (**a**) Genetic linkage; (**b**) pleiotropy ① including physiological interaction ② and the combination of pleiotropy and physiological interaction ③.

**Table 1:** Phenotypic variation of SNPP for Zhongshuang11 and No. 73290 as well as the derived RIL population in four investigated environments.

Experiment code	Parents			RIL population		
	Zhongshuang11	No. 73290	*p*_t-test_	Min	Max	Mean
W11	22.2 ± 1.6	12.7 ± 2.2	3.7E–10	6.3	25.1	15.6 ± 3.0
Z11	21.4 ± 1.6	11.1 ± 1.9	1.2E–17	7.3	27.3	17.7 ± 3.7
W12	21.7 ± 1.4	11.6 ± 1.1	5.6E–21	7.3	19.2	11.1 ± 3.8
Z12	21.3 ± 1.7	12.2 ± 1.4	2.1E–08	7.3	22.9	14.7 ± 3.6
Mean	21.7 ± 1.6	11.7 ± 1.4	2.0E–55	7.0	23.6	14.8 ± 2.8

**Table 2:** Consensus QTLs for SNPP.

Consensus QTL	Linkage group	Peak position	Confidence Interval	Genomic regions (Mb)	Additive effect	LOD value	*R*^2^(%)	Experiment code
*qSN.A02*	A02	4.2	3.0–5.0	32.8–39.4	2.16	16.7	24.8	Z11RIL
*qSN.A06*	A06	122.6	113.5–133.7	21.6–23.3	1.58	11.6	20.1	W11RIL/Z11RIL/W12RIL/Z12RIL
*qSN.C01*	C01	0.0	0.0–3.4	1.2–1.6	−0.77	4.3	6.2	W11RIL
*qSN.C04*	C04	129.6	129.2–129.6	45.5–45.7	−0.87	4.1	5.3	Z11RIL
*qSN.C06*	C06	54.5	50.7–58.2	18.9–20.4	0.96	5.74	8.18	W11RIL/Z11RIL

**Table 3:** QTL Genotypes and SNPP of ten types of recombinant NILs and the two parents.

	Lines	Number in BC_4_F_3_	Genotype of the six markers within QTL interval						SNPP in the BC_4_F_3_ lines	
			BrSF47-389	BrSF47-234	BrSF47-10	BrSF46-167	BrSF46-306	ni108	Mean ± SD	*P*
Parents	Zhongshuang11	30	A	A	A	A	A	A	21.2 ± 1.3	–
	No. 73290	30	B	B	B	B	B	B	12.2 ± 1.5	< 0.0001
NILs	Type−1	35	B	A	A	A	A	A	21.2 ± 1.7	0.3620
	Type−2	34	B	B	A	A	A	A	21.4 ± 2.2	0.7411
	Type−3	40	B	B	B	A	A	A	16.4 ± 1.9	< 0.0001
	Type−4	32	B	B	B	B	A	A	16.4 ± 1.7	< 0.0001
	Type−5	33	B	B	B	B	B	A	16.7 ± 2.2	< 0.0001
	Type−6	36	A	B	B	B	B	B	15.9 ± 0.8	< 0.0001
	Type−7	58	A	A	B	B	B	B	16.1 ± 2.2	< 0.0001
	Type−8	30	A	A	A	B	B	B	16.5 ± 0.3	< 0.0001
	Type−9	37	A	A	A	A	B	B	21.5 ± 1.6	0.8382
	Type−10	33	A	A	A	A	A	B	21.8 ± 1.0	0.6545

Genotype A and B represented homologous alleles from Zhongshuang11 and No. 73290, respectively.

**Table 4:** Yield traits at harvest for Zhongshuang11 and NIL-*qSN.A6.*

	Trait	Zhongshuang11	NIL-*qSN.A6*	*P*-Value	Rate
Yield	SY (g)	19.4 ± 0.9	16.0 ± 1.0	4.7E–02	17.4%
Yield component	SNPP	21.2 ± 0.5	15.9 ± 1.2	6.3E–10	25.1%
	SW (g)	4.29 ± 0.06	4.68 ± 0.24	8.4E–07	−9.09%
	PN	261 ± 5	273 ± 8	8.8E–01	−4.6%
Yield related	PL (mm)	84.9 ± 2.1	81.9 ± 3.8	9.3E–02	3.5%
	PH (cm)	185 ± 3	182 ± 3	6.2E–02	1.6%
	BN	6.20 ± 0.81	6.63 ± 1.10	3.9E–01	−6.5%
	MIL (cm)	46.2 ± 4.4	47.4 ± 3.1	7.7E–01	−2.2%
	Flowering time (days)	191 ± 3	191 ± 5	7.3E–01	0.0%
	Maturity time (days)	214 ± 6	216 ± 4	4.5E–01	−0.9%

Trait data are expressed as the means ± SD measured from 30 different plants. SY, SW, PL, PN, PH, BH and MIL represented seed yield per plant, seed weight, pod length, pod number, plant height, branch number and main inflorescence, respectively.

**Table 5:** Conditional analysis of *qSN.A6* on SNPP and SW in the RIL population.

Experiment code	LOD/*R*^*2*^(%)			
	SNPP	SNPP|SW	SW	SW|SNPP
W11	14.5/25.7	14.0/24.1	5.4/5.4	NA
Z11	10.3/24.5	7.8/16.9	3.5/9.0	NA
W12	3.1/4.6	8.4/12.9	2.9/4.5	NA
Z12	5.2/12.0	4.6/5.3	NA	NA

NA represented non-significant.
