# Supplementary material for: Genetic architecture and mechanism of seed number per pod in rapeseed: elucidated through linkage and near-isogenic line analysis
Source: Sci Rep. 2016 Apr 12;6:24124. doi: 10.1038/srep24124 (PMC4828700; doi:10.1038/srep24124)
Supplement: Supplementary Information [file srep24124-s1.doc]

**Supplementary Information**

**Genetic architecture and mechanism of seed number per pod in rapeseed: elucidated through linkage and near-isogenic line analysis**

Yuhua Yang^#^, Jiaqin Shi^#^, Xinfa Wang, Guihua Liu and Hanzhong Wang^*^

Oil Crops Research Institute of the Chinese Academy of Agricultural Sciences, Key Laboratory of Biology and Genetic Improvement of Oil Crops, Ministry of Agriculture, Wuhan 430062, China

^#^ These authors contributed equally to this work.

^*^ Corresponding author: Hanzhong Wang

E-mail: [wanghz@oilcrops.cn](mailto:wanghz@oilcrops.cn);

Tel: +86 010-82103016;

Fax: +86 027-86836125.

# Table S1. The constructed genetic linkage map for the RIL population

| Linkage group | Marker | Position (cM) |
| --- | --- | --- |
| A01 | Bn-A01-p102249 | 0 |
| A01 | Bn-A01-p146963 | 1.028 |
| A01 | Bn-A01-p195253 | 1.615 |
| A01 | Bn-A01-p178709 | 1.748 |
| A01 | Bn-A01-p147990 | 2.201 |
| A01 | Bn-A01-p644410 | 6.386 |
| A01 | Bn-A01-p436141 | 23.036 |
| A01 | Bn-A01-p2634563 | 26.144 |
| A01 | Bn-A01-p2699907 | 26.611 |
| A01 | Bn-A01-p2891863 | 27.073 |
| A01 | Bn-A01-p2718126 | 29.141 |
| A01 | Bn-A01-p3005310 | 29.775 |
| A01 | Bn-A01-p2874393 | 31.324 |
| A01 | Bn-A01-p3134159 | 32.824 |
| A01 | Bn-A01-p2448162 | 34.11 |
| A01 | Bn-A01-p975213 | 35.106 |
| A01 | Bn-A01-p1029668 | 35.981 |
| A01 | Bn-A01-p1001022 | 36.062 |
| A01 | Bn-A01-p1194009 | 36.183 |
| A01 | Bn-A01-p1152123 | 36.297 |
| A01 | Bn-A01-p1324586 | 36.368 |
| A01 | Bn-A01-p1133447 | 36.424 |
| A01 | Bn-A01-p1275284 | 36.646 |
| A01 | Bn-A01-p1125565 | 36.847 |
| A01 | Bn-A01-p1373838 | 36.918 |
| A01 | Bn-A01-p1373965 | 37.068 |
| A01 | Bn-A01-p877322 | 37.256 |
| A01 | Bn-scaff_15838_1-p517875 | 37.333 |
| A01 | Bn-A01-p1448811 | 38.383 |
| A01 | Bn-A01-p2569303 | 38.868 |
| A01 | Bn-A01-p2307144 | 39.344 |
| A01 | Bn-A01-p2453633 | 39.524 |
| A01 | Bn-A01-p2453758 | 39.552 |
| A01 | Bn-A01-p2509163 | 39.743 |
| A01 | Bn-A01-p2318972 | 39.99 |
| A01 | Bn-A01-p2323744 | 40.253 |
| A01 | Bn-A01-p2254004 | 40.568 |
| A01 | Bn-A01-p2341408 | 40.792 |
| A01 | Bn-A01-p2207452 | 41.007 |
| A01 | Bn-A01-p2121224 | 41.253 |
| A01 | Bn-A01-p3758784 | 41.566 |
| A01 | Bn-A01-p1551700 | 41.839 |
| A01 | Bn-A01-p1880406 | 41.961 |
| A01 | Bn-A01-p1661180 | 42.167 |
| A01 | Bn-A01-p1666732 | 42.201 |
| A01 | Bn-A01-p2004336 | 42.341 |
| A01 | Bn-A01-p1745099 | 42.514 |
| A01 | Bn-A01-p1746226 | 42.667 |
| A01 | Bn-A01-p1975231 | 42.835 |
| A01 | Bn-A01-p1580970 | 42.978 |
| A01 | Bn-A01-p1530160 | 43.081 |
| A01 | Bn-A01-p4506540 | 43.717 |
| A01 | Bn-A01-p4293847 | 43.805 |
| A01 | Bn-A01-p4354753 | 44.344 |
| A01 | Bn-A01-p4322340 | 49.14 |
| A01 | Bn-A01-p3744726 | 50.568 |
| A01 | Bn-A01-p3938241 | 50.916 |
| A01 | Bn-A01-p5570221 | 53.233 |
| A01 | Bn-A01-p5868598 | 53.382 |
| A01 | Bn-A01-p5550226 | 53.652 |
| A01 | Bn-A01-p5378430 | 54.708 |
| A01 | Bn-A01-p5335218 | 54.866 |
| A01 | Bn-A01-p5601232 | 55.957 |
| A01 | Bn-A01-p5586618 | 56.147 |
| A01 | Bn-A01-p5835558 | 56.352 |
| A01 | Bn-A01-p5570110 | 56.863 |
| A01 | Bn-A01-p4947577 | 58.002 |
| A01 | Bn-A01-p4920622 | 59.6 |
| A01 | Bn-A01-p4908924 | 61.106 |
| A01 | Bn-A01-p4785261 | 61.75 |
| A01 | Bn-A01-p4786787 | 62.049 |
| A01 | Bn-A01-p6687839 | 67.698 |
| A01 | Bn-A01-p6708539 | 67.866 |
| A01 | Bn-A01-p6877281 | 68.304 |
| A01 | Bn-A01-p6825673 | 68.883 |
| A01 | Bn-A01-p8017380 | 75.761 |
| A01 | Bn-A01-p8014995 | 76.362 |
| A01 | Bn-A01-p8051029 | 76.89 |
| A01 | Bn-A01-p9996240 | 85.334 |
| A01 | Bn-scaff_27129_1-p129919 | 85.418 |
| A01 | Bn-A01-p10105499 | 85.59 |
| A01 | Bn-A01-p10027328 | 86.38 |
| A01 | Bn-A01-p9982115 | 86.616 |
| A01 | Bn-scaff_16217_1-p143344 | 87.702 |
| A01 | Bn-A01-p10344498 | 88.419 |
| A01 | Bn-A01-p10611800 | 89.3 |
| A01 | Bn-A01-p10835605 | 89.874 |
| A01 | Bn-A01-p11441851 | 92.036 |
| A01 | Bn-A01-p11462315 | 92.205 |
| A01 | Bn-A01-p11459295 | 92.402 |
| A01 | Bn-A01-p11273927 | 93.035 |
| A01 | Bn-A01-p11702957 | 93.378 |
| A01 | Bn-A01-p12233209 | 94.144 |
| A01 | Bn-A01-p12077110 | 94.299 |
| A01 | Bn-scaff_22790_1-p1317315 | 94.518 |
| A01 | Bn-A01-p12454306 | 94.591 |
| A01 | Bn-A01-p12427149 | 94.644 |
| A01 | Bn-A01-p12535612 | 95.058 |
| A01 | Bn-A01-p12544054 | 95.203 |
| A01 | Bn-A01-p16086381 | 98.4 |
| A01 | Bn-A01-p16134838 | 98.974 |
| A01 | Bn-A01-p16200217 | 99.19 |
| A01 | Bn-A01-p16380496 | 99.644 |
| A01 | Bn-A01-p16720365 | 99.973 |
| A01 | Bn-Scaffold000582-p3247 | 100.204 |
| A01 | Bn-A01-p16730334 | 100.221 |
| A01 | Bn-A01-p16715637 | 100.59 |
| A01 | Bn-A01-p16749951 | 100.878 |
| A01 | Bn-A01-p16774157 | 101.057 |
| A01 | Bn-A01-p16770716 | 101.118 |
| A01 | Bn-A01-p17105372 | 101.34 |
| A01 | Bn-A01-p16857466 | 101.442 |
| A01 | Bn-A01-p17150875 | 101.533 |
| A01 | Bn-A01-p17121291 | 101.756 |
| A01 | Bn-A01-p17157752 | 101.761 |
| A01 | Bn-A01-p17081120 | 101.826 |
| A01 | Bn-A01-p17129906 | 101.953 |
| A01 | Bn-A01-p17049439 | 102.11 |
| A01 | Bn-A01-p17278340 | 102.404 |
| A01 | Bn-A01-p17494364 | 102.724 |
| A01 | Bn-A01-p17377721 | 102.865 |
| A01 | Bn-A01-p17518104 | 102.959 |
| A01 | Bn-A01-p17527782 | 102.982 |
| A01 | Bn-A01-p18330598 | 103.101 |
| A01 | Bn-A01-p18096974 | 103.124 |
| A01 | Bn-A01-p17845660 | 103.21 |
| A01 | Bn-A01-p17408107 | 103.319 |
| A01 | Bn-A01-p17935219 | 103.337 |
| A01 | Bn-A01-p17769742 | 103.346 |
| A01 | Bn-A01-p17887935 | 103.36 |
| A01 | Bn-A01-p18178802 | 103.635 |
| A01 | Bn-A01-p18200921 | 103.755 |
| A01 | Bn-A01-p18121827 | 103.828 |
| A01 | Bn-A01-p18027200 | 103.849 |
| A01 | Bn-A01-p18020258 | 103.873 |
| A01 | Bn-A01-p19340658 | 105.609 |
| A01 | Bn-A01-p19449612 | 106.435 |
| A01 | Bn-A01-p19722138 | 106.849 |
| A01 | Bn-A01-p19879925 | 107.664 |
| A01 | Bn-A01-p20181046 | 108.654 |
| A01 | Bn-A01-p20167230 | 108.877 |
| A01 | Bn-A01-p20207440 | 108.906 |
| A01 | Bn-A01-p20211387 | 109.256 |
| A01 | Bn-A01-p20246840 | 109.422 |
| A01 | Bn-A01-p20442457 | 109.798 |
| A01 | Bn-A01-p20397807 | 109.814 |
| A01 | Bn-A01-p20323059 | 109.942 |
| A01 | Bn-Scaffold001779-p127 | 110.678 |
| A01 | Bn-A01-p20745330 | 110.887 |
| A01 | Bn-A01-p20814699 | 110.926 |
| A01 | Bn-A01-p20836664 | 110.952 |
| A01 | Bn-A01-p21106687 | 112.162 |
| A01 | Bn-scaff_15879_1-p426186 | 112.187 |
| A01 | Bn-A01-p21079909 | 112.197 |
| A01 | Bn-A01-p21038677 | 112.545 |
| A01 | Bn-A01-p21141514 | 112.814 |
| A01 | Bn-A01-p21500509 | 114.666 |
| A01 | Bn-A01-p21512663 | 114.776 |
| A01 | Bn-A01-p21512514 | 114.818 |
| A01 | Bn-A01-p21605865 | 115.037 |
| A01 | Bn-A01-p21709916 | 115.558 |
| A01 | Bn-A01-p21715162 | 115.687 |
| A01 | Bn-A01-p21532512 | 116.013 |
| A01 | Bn-A01-p23478530 | 120.79 |
| A01 | Bn-A01-p23196022 | 121.312 |
| A01 | Bn-A01-p23181293 | 121.348 |
| A01 | Bn-A01-p23087044 | 121.419 |
| A01 | Bn-A01-p23096147 | 121.539 |
| A01 | Bn-A01-p22999151 | 121.891 |
| A01 | Bn-A01-p23538856 | 122.224 |
| A01 | Bn-A01-p23538267 | 122.327 |
| A01 | Bn-A01-p23473514 | 122.332 |
| A01 | Bn-A01-p27263558 | 137.261 |
| A01 | Bn-A01-p27389703 | 138.156 |
| A01 | Bn-A01-p27580681 | 139.368 |
| A01 | Bn-scaff_24117_1-p20746 | 143.31 |
| A01 | Bn-A01-p27846544 | 143.623 |
| A01 | Bn-A01-p27926195 | 143.805 |
| A01 | Bn-A01-p27926866 | 144.334 |
| A01 | Bn-A01-p27953261 | 144.578 |
| A01 | Bn-A01-p28278192 | 145.21 |
| A01 | Bn-A01-p25512934 | 147.596 |
| A01 | Bn-A01-p25509783 | 147.859 |
| A01 | Bn-A01-p25572254 | 148.054 |
| A02 | Bn-A02-p2572194 | 0 |
| A02 | Bn-A02-p2582999 | 0.272 |
| A02 | Bn-A02-p2572625 | 0.552 |
| A02 | Bn-A02-p2608125 | 0.927 |
| A02 | Bn-A02-p2542065 | 1.161 |
| A02 | Bn-A02-p2536779 | 1.291 |
| A02 | Bn-A02-p2535232 | 1.612 |
| A02 | Bn-A02-p2692116 | 2.206 |
| A02 | Bn-A02-p2642904 | 2.35 |
| A02 | Bn-A02-p1276717 | 3.296 |
| A02 | Bn-A02-p1341101 | 3.332 |
| A02 | Bn-A02-p2788144 | 3.736 |
| A02 | Bn-A02-p1232964 | 4.18 |
| A02 | Bn-A02-p2799146 | 4.222 |
| A02 | Bn-A02-p1092146 | 4.638 |
| A02 | Bn-A02-p2851231 | 5.396 |
| A02 | Bn-scaff_15714_1-p1934829 | 7.063 |
| A02 | Bn-A02-p3281452 | 7.83 |
| A02 | Bn-A02-p3295898 | 7.841 |
| A02 | Bn-A02-p3175610 | 8.483 |
| A02 | Bn-A02-p3207085 | 8.928 |
| A02 | Bn-A02-p3543810 | 13.813 |
| A02 | Bn-A02-p4259381 | 20.963 |
| A02 | Bn-A02-p4277649 | 21.426 |
| A02 | Bn-A02-p4262907 | 22.002 |
| A02 | Bn-A02-p5326222 | 34.057 |
| A02 | Bn-A02-p5924483 | 34.501 |
| A02 | Bn-A02-p5925648 | 34.765 |
| A02 | Bn-A02-p5925766 | 34.807 |
| A02 | Bn-A02-p5582861 | 35.227 |
| A02 | Bn-A02-p5612147 | 35.754 |
| A02 | Bn-A02-p5625172 | 35.854 |
| A02 | Bn-A02-p5661524 | 35.885 |
| A02 | Bn-A02-p6167491 | 36.475 |
| A02 | Bn-A02-p6360867 | 36.975 |
| A02 | Bn-A02-p6084757 | 37.185 |
| A02 | Bn-Scaffold009013-p209 | 37.706 |
| A02 | Bn-A02-p6843407 | 38.014 |
| A02 | Bn-A02-p6845958 | 38.049 |
| A02 | Bn-scaff_16269_1-p242999 | 38.469 |
| A02 | Bn-A02-p6587201 | 38.627 |
| A02 | Bn-A02-p6461773 | 38.81 |
| A02 | Bn-A02-p6816262 | 38.99 |
| A02 | Bn-A02-p7043311 | 39.363 |
| A02 | Bn-A02-p7043901 | 39.485 |
| A02 | Bn-A02-p7439205 | 40.127 |
| A02 | Bn-A02-p7463965 | 40.168 |
| A02 | Bn-A02-p7516226 | 40.936 |
| A02 | Bn-A02-p7736604 | 41.733 |
| A02 | Bn-A02-p7675505 | 42.21 |
| A02 | Bn-A02-p7675384 | 42.559 |
| A02 | Bn-A02-p8024157 | 43.196 |
| A02 | Bn-A02-p7893025 | 43.295 |
| A02 | Bn-A02-p7893461 | 43.305 |
| A02 | Bn-A02-p7958421 | 43.545 |
| A02 | Bn-A02-p7840077 | 43.836 |
| A02 | Bn-A02-p7784914 | 43.968 |
| A02 | Bn-A02-p7788379 | 44.064 |
| A02 | Bn-A02-p8199891 | 44.365 |
| A02 | Bn-A02-p8176181 | 44.5 |
| A02 | Bn-A02-p7926823 | 44.594 |
| A02 | Bn-A02-p7978230 | 44.671 |
| A02 | Bn-A02-p8234978 | 44.681 |
| A02 | Bn-A02-p7885731 | 45.012 |
| A02 | Bn-A02-p8404663 | 45.307 |
| A02 | Bn-A02-p8527757 | 45.655 |
| A02 | Bn-A02-p8538437 | 45.671 |
| A02 | Bn-A02-p8559435 | 46.033 |
| A02 | Bn-A02-p8571577 | 46.071 |
| A02 | Bn-A02-p8549867 | 46.255 |
| A02 | Bn-A02-p8509407 | 46.334 |
| A02 | Bn-A02-p8999771 | 46.518 |
| A02 | Bn-A02-p8954851 | 46.633 |
| A02 | Bn-A02-p8884712 | 46.838 |
| A02 | Bn-A02-p8836098 | 46.986 |
| A02 | Bn-A02-p8720739 | 46.993 |
| A02 | Bn-A02-p8726802 | 46.995 |
| A02 | Bn-A02-p8873630 | 47.027 |
| A02 | Bn-A02-p8873864 | 47.028 |
| A02 | Bn-A02-p8895388 | 47.072 |
| A02 | Bn-A02-p8881251 | 47.125 |
| A02 | Bn-A02-p8466214 | 47.501 |
| A02 | Bn-A02-p8323616 | 47.941 |
| A02 | Bn-A02-p8336294 | 48.045 |
| A02 | Bn-A02-p8453414 | 48.198 |
| A02 | Bn-A02-p8391383 | 48.421 |
| A02 | Bn-A02-p7775332 | 48.799 |
| A02 | Bn-A02-p9246552 | 49.277 |
| A02 | Bn-A02-p9229113 | 49.47 |
| A02 | Bn-A02-p9225798 | 49.733 |
| A02 | Bn-A02-p9255686 | 50.203 |
| A02 | Bn-A02-p10096185 | 51.293 |
| A02 | Bn-A02-p9993945 | 51.717 |
| A02 | Bn-A02-p10193709 | 52.088 |
| A02 | Bn-A02-p10227986 | 52.834 |
| A02 | Bn-A02-p10625720 | 53.11 |
| A02 | Bn-A02-p10623838 | 53.224 |
| A02 | Bn-A02-p10626782 | 53.608 |
| A02 | Bn-A02-p10866642 | 54.186 |
| A02 | Bn-A02-p11342711 | 55.489 |
| A02 | Bn-A02-p12164268 | 57.312 |
| A02 | Bn-A02-p12150733 | 57.736 |
| A02 | Bn-A02-p12192921 | 57.737 |
| A02 | Bn-A02-p11896294 | 57.756 |
| A02 | Bn-A02-p11939715 | 57.98 |
| A02 | Bn-A02-p12269431 | 58.518 |
| A02 | Bn-A02-p12284671 | 58.519 |
| A02 | Bn-A02-p12393617 | 58.659 |
| A02 | Bn-A02-p12003125 | 58.818 |
| A02 | Bn-A02-p12297473 | 59.173 |
| A02 | Bn-A02-p12295281 | 59.18 |
| A02 | Bn-A02-p12061350 | 59.529 |
| A02 | Bn-A02-p12508852 | 60.05 |
| A02 | Bn-A02-p12552323 | 60.546 |
| A02 | Bn-A02-p12550795 | 60.793 |
| A02 | Bn-A02-p12514179 | 60.809 |
| A02 | Bn-A02-p12516859 | 60.849 |
| A02 | Bn-A02-p12627047 | 61.672 |
| A02 | Bn-A02-p12642708 | 61.731 |
| A02 | Bn-A02-p12681201 | 62.173 |
| A02 | Bn-A02-p12996998 | 63.008 |
| A02 | Bn-A02-p13212386 | 65.203 |
| A02 | Bn-A02-p14028000 | 67.045 |
| A02 | Bn-A02-p14151274 | 68.127 |
| A02 | Bn-A02-p14103759 | 68.608 |
| A02 | Bn-A02-p14175730 | 68.617 |
| A02 | Bn-A02-p14293315 | 70.929 |
| A02 | Bn-A02-p14898010 | 71.313 |
| A02 | Bn-A02-p14925274 | 71.349 |
| A02 | Bn-A02-p14576479 | 71.607 |
| A02 | Bn-A02-p14992706 | 71.951 |
| A02 | Bn-A02-p14775699 | 72.844 |
| A02 | Bn-A02-p24366221 | 92.948 |
| A02 | Bn-A02-p24237359 | 93.106 |
| A02 | Bn-A02-p24269435 | 93.142 |
| A02 | Bn-scaff_17623_1-p648231 | 94.061 |
| A02 | Bn-A02-p24687309 | 94.495 |
| A02 | Bn-scaff_17623_1-p569874 | 95.104 |
| A02 | Bn-A02-p24552476 | 95.13 |
| A02 | Bn-A02-p24552203 | 95.155 |
| A02 | Bn-A02-p24552840 | 95.259 |
| A02 | Bn-A02-p24552639 | 95.286 |
| A02 | Bn-A02-p24911505 | 98.02 |
| A02 | Bn-A02-p24909771 | 98.187 |
| A03 | Bn-A03-p235039 | 0 |
| A03 | Bn-A03-p49020 | 0.496 |
| A03 | Bn-A03-p12026 | 1.032 |
| A03 | Bn-A03-p532166 | 1.197 |
| A03 | Bn-A03-p166402 | 1.33 |
| A03 | Bn-A03-p166292 | 1.682 |
| A03 | Bn-A03-p835162 | 2.008 |
| A03 | Bn-A03-p766322 | 2.25 |
| A03 | Bn-A03-p835987 | 2.529 |
| A03 | Bn-A03-p832473 | 3.232 |
| A03 | Bn-A03-p834379 | 3.238 |
| A03 | Bn-A03-p951365 | 3.452 |
| A03 | Bn-A03-p924676 | 3.679 |
| A03 | Bn-A03-p1008445 | 4.608 |
| A03 | Bn-scaff_16614_1-p677445 | 7.475 |
| A03 | Bn-A03-p1475495 | 11.917 |
| A03 | Bn-A03-p1698016 | 12.912 |
| A03 | Bn-A03-p2074890 | 15.192 |
| A03 | Bn-A03-p2130281 | 16.064 |
| A03 | Bn-A03-p2203942 | 16.387 |
| A03 | Bn-A03-p2366510 | 17.569 |
| A03 | Bn-A03-p2493541 | 21.326 |
| A03 | Bn-A03-p2596410 | 23.714 |
| A03 | Bn-A03-p3073797 | 25.544 |
| A03 | Bn-A03-p3115732 | 26.07 |
| A03 | Bn-A03-p3877500 | 33.462 |
| A03 | Bn-A03-p3877572 | 33.529 |
| A03 | Bn-A03-p4232493 | 35.181 |
| A03 | Bn-A03-p4196989 | 35.908 |
| A03 | Bn-A03-p4309562 | 38.351 |
| A03 | Bn-A03-p4295145 | 38.786 |
| A03 | Bn-A03-p4423212 | 39.587 |
| A03 | Bn-A03-p4407987 | 39.806 |
| A03 | Bn-A03-p4423604 | 40.31 |
| A03 | Bn-A03-p4409606 | 40.403 |
| A03 | Bn-A03-p4507718 | 41.282 |
| A03 | Bn-A03-p4498766 | 41.842 |
| A03 | Bn-A03-p4590174 | 44.957 |
| A03 | Bn-A03-p4593298 | 45.118 |
| A03 | Bn-A03-p4567578 | 45.357 |
| A03 | Bn-A03-p5360744 | 48.436 |
| A03 | Bn-A03-p5374417 | 48.69 |
| A03 | Bn-scaff_22728_1-p75030 | 48.722 |
| A03 | Bn-A03-p5170706 | 49.295 |
| A03 | Bn-A03-p5180655 | 49.319 |
| A03 | Bn-A03-p5414946 | 49.507 |
| A03 | Bn-A03-p5892557 | 50.252 |
| A03 | Bn-A03-p5956934 | 50.26 |
| A03 | Bn-A03-p5893002 | 50.271 |
| A03 | Bn-A03-p5564849 | 50.804 |
| A03 | Bn-A03-p5613665 | 51.174 |
| A03 | Bn-A03-p5612769 | 51.275 |
| A03 | Bn-A03-p5711923 | 51.491 |
| A03 | Bn-A03-p5809860 | 51.562 |
| A03 | Bn-A03-p5713023 | 51.67 |
| A03 | Bn-A03-p5643775 | 51.957 |
| A03 | Bn-A03-p5741340 | 52.065 |
| A03 | Bn-A03-p5749832 | 52.15 |
| A03 | Bn-A03-p5748775 | 52.336 |
| A03 | Bn-A03-p6228570 | 53.076 |
| A03 | Bn-A03-p5563280 | 53.501 |
| A03 | Bn-A03-p6332797 | 53.84 |
| A03 | Bn-A03-p6420355 | 54.451 |
| A03 | Bn-A03-p6425858 | 54.623 |
| A03 | Bn-A03-p6428220 | 54.707 |
| A03 | Bn-A03-p6585645 | 55.653 |
| A03 | Bn-A03-p6717785 | 55.999 |
| A03 | Bn-A03-p6646094 | 56.051 |
| A03 | Bn-A03-p6744344 | 56.405 |
| A03 | Bn-A03-p6804857 | 56.656 |
| A03 | Bn-A03-p6804536 | 56.757 |
| A03 | Bn-A03-p6771876 | 56.889 |
| A03 | Bn-A03-p6765024 | 56.933 |
| A03 | Bn-A03-p6772326 | 56.992 |
| A03 | Bn-A03-p6744274 | 57.297 |
| A03 | Bn-A03-p6791851 | 57.462 |
| A03 | Bn-A03-p6898220 | 57.608 |
| A03 | Bn-A03-p7090745 | 57.896 |
| A03 | Bn-A10-p1629545 | 57.951 |
| A03 | Bn-A03-p7007377 | 57.989 |
| A03 | Bn-A03-p7049396 | 58.201 |
| A03 | Bn-A03-p6387774 | 58.636 |
| A03 | Bn-A03-p7180288 | 59.089 |
| A03 | Bn-A03-p7284090 | 60.214 |
| A03 | Bn-A03-p7356882 | 60.683 |
| A03 | Bn-A03-p7357257 | 61.144 |
| A03 | Bn-A03-p7500107 | 61.701 |
| A03 | Bn-A03-p7514862 | 61.741 |
| A03 | Bn-A03-p7514571 | 61.75 |
| A03 | Bn-A03-p7514678 | 61.759 |
| A03 | Bn-A03-p7505194 | 61.792 |
| A03 | Bn-A03-p7378514 | 62.314 |
| A03 | Bn-A03-p7501352 | 62.605 |
| A03 | Bn-A03-p7571364 | 63.038 |
| A03 | Bn-A03-p7575620 | 63.053 |
| A03 | Bn-A03-p7613494 | 63.102 |
| A03 | Bn-A03-p7630628 | 63.327 |
| A03 | Bn-A03-p7631083 | 63.367 |
| A03 | Bn-A03-p7627590 | 63.63 |
| A03 | Bn-A03-p7642519 | 64.534 |
| A03 | Bn-A03-p7643795 | 64.986 |
| A03 | Bn-scaff_21312_1-p1156786 | 65.253 |
| A03 | Bn-A03-p7672403 | 65.625 |
| A03 | Bn-scaff_21312_1-p1111796 | 65.631 |
| A03 | Bn-A03-p7760102 | 65.95 |
| A03 | Bn-A03-p7761061 | 66.074 |
| A03 | Bn-A03-p7773812 | 66.103 |
| A03 | Bn-A03-p7814324 | 66.485 |
| A03 | Bn-A03-p7919195 | 67.24 |
| A03 | Bn-scaff_21312_1-p932709 | 67.392 |
| A03 | Bn-A03-p7969685 | 67.54 |
| A03 | Bn-A03-p7966805 | 67.767 |
| A03 | Bn-A03-p8033095 | 68.036 |
| A03 | Bn-A03-p7835836 | 68.23 |
| A03 | Bn-A03-p8152842 | 68.965 |
| A03 | Bn-A03-p8260551 | 69.366 |
| A03 | Bn-A03-p8181125 | 69.537 |
| A03 | Bn-A03-p8177644 | 69.674 |
| A03 | Bn-A03-p8181778 | 69.743 |
| A03 | Bn-A03-p8398790 | 70.971 |
| A03 | Bn-A03-p8396525 | 71.287 |
| A03 | Bn-A03-p8337378 | 71.495 |
| A03 | Bn-A03-p8333536 | 71.571 |
| A03 | Bn-A03-p8308938 | 71.686 |
| A03 | Bn-A03-p8483706 | 72.146 |
| A03 | Bn-A03-p8554739 | 72.518 |
| A03 | Bn-A03-p8638217 | 72.597 |
| A03 | Bn-A03-p8497543 | 72.86 |
| A03 | Bn-A03-p8631833 | 73.071 |
| A03 | Bn-A03-p8663803 | 73.462 |
| A03 | Bn-A03-p8756590 | 74.227 |
| A03 | Bn-A03-p8764481 | 74.662 |
| A03 | Bn-A03-p8741978 | 75.103 |
| A03 | Bn-A03-p8805441 | 75.529 |
| A03 | Bn-A03-p8842546 | 76.026 |
| A03 | Bn-A03-p8924852 | 76.391 |
| A03 | Bn-A03-p8956355 | 76.691 |
| A03 | Bn-A03-p8957734 | 76.723 |
| A03 | Bn-A03-p9020167 | 76.827 |
| A03 | Bn-scaff_23954_1-p239074 | 77.402 |
| A03 | Bn-A03-p9143356 | 77.488 |
| A03 | Bn-A03-p9203389 | 78.553 |
| A03 | Bn-A03-p9203992 | 78.644 |
| A03 | Bn-A03-p9255139 | 78.652 |
| A03 | Bn-A03-p9343295 | 79.232 |
| A03 | Bn-A03-p9338698 | 79.248 |
| A03 | Bn-A03-p9497697 | 79.868 |
| A03 | Bn-A03-p9537618 | 80.128 |
| A03 | Bn-A03-p9641671 | 80.503 |
| A03 | Bn-A03-p9765420 | 81.624 |
| A03 | Bn-A03-p9818699 | 82.089 |
| A03 | Bn-A03-p10047998 | 82.329 |
| A03 | Bn-A03-p9925550 | 82.588 |
| A03 | Bn-A03-p10086496 | 82.743 |
| A03 | Bn-A03-p9926783 | 82.929 |
| A03 | Bn-A03-p9939018 | 83.1 |
| A03 | Bn-A03-p10293071 | 83.43 |
| A03 | Bn-A03-p10248162 | 83.472 |
| A03 | Bn-A03-p9856923 | 83.912 |
| A03 | Bn-A03-p10466633 | 84.451 |
| A03 | Bn-A03-p10313368 | 84.793 |
| A03 | Bn-A03-p10566503 | 85.64 |
| A03 | Bn-A03-p10617866 | 85.795 |
| A03 | Bn-A03-p10833240 | 86.49 |
| A03 | Bn-A03-p10773339 | 87.204 |
| A03 | Bn-A03-p10862251 | 87.68 |
| A03 | Bn-A03-p10969109 | 88.745 |
| A03 | Bn-scaff_22466_1-p748313 | 91.055 |
| A03 | Bn-A03-p11232586 | 91.279 |
| A03 | Bn-A03-p11227755 | 91.446 |
| A03 | Bn-A03-p11191805 | 91.662 |
| A03 | Bn-A03-p11071320 | 92.166 |
| A03 | Bn-A03-p11070958 | 92.486 |
| A03 | Bn-A03-p11053857 | 92.601 |
| A03 | Bn-A03-p11325897 | 93.172 |
| A03 | Bn-A03-p11356180 | 93.435 |
| A03 | Bn-A03-p11256138 | 93.602 |
| A03 | Bn-A03-p11429520 | 93.862 |
| A03 | Bn-A03-p11381125 | 94.025 |
| A03 | Bn-A03-p11505658 | 94.235 |
| A03 | Bn-A03-p11519012 | 94.238 |
| A03 | Bn-A03-p11519264 | 94.27 |
| A03 | Bn-A03-p11655749 | 94.841 |
| A03 | Bn-A03-p11684352 | 95.232 |
| A03 | Bn-A03-p11751694 | 95.399 |
| A03 | Bn-A03-p11822847 | 95.8 |
| A03 | Bn-A03-p12186958 | 97.651 |
| A03 | Bn-A03-p12331575 | 97.907 |
| A03 | Bn-A03-p12250401 | 97.915 |
| A03 | Bn-A03-p12322277 | 98.126 |
| A03 | Bn-A03-p12342812 | 98.373 |
| A03 | Bn-A03-p12359936 | 98.497 |
| A03 | Bn-A03-p12286718 | 99.352 |
| A03 | Bn-A03-p12279706 | 99.59 |
| A03 | Bn-A03-p12279241 | 99.738 |
| A03 | Bn-A03-p12863264 | 102.785 |
| A03 | Bn-A03-p13302882 | 103.139 |
| A03 | Bn-A03-p13104283 | 103.908 |
| A03 | Bn-A03-p13169346 | 104.081 |
| A03 | Bn-A03-p13545223 | 106.968 |
| A03 | Bn-A03-p14003701 | 108.436 |
| A03 | Bn-A03-p14037892 | 109.147 |
| A03 | Bn-A03-p13996014 | 110.036 |
| A03 | Bn-A03-p14310386 | 110.809 |
| A03 | Bn-A03-p15139190 | 114.807 |
| A03 | Bn-A03-p15004059 | 115.809 |
| A03 | Bn-A03-p15708289 | 117.607 |
| A03 | Bn-A03-p15605355 | 117.926 |
| A03 | Bn-A03-p15622267 | 118.232 |
| A03 | Bn-A03-p15627698 | 118.35 |
| A03 | Bn-A03-p15960376 | 119.926 |
| A03 | Bn-A03-p16126013 | 122.548 |
| A03 | Bn-A03-p16379045 | 125.859 |
| A03 | Bn-A03-p16378233 | 126.315 |
| A03 | Bn-A03-p17182494 | 130.664 |
| A03 | Bn-A03-p21841965 | 152.134 |
| A03 | Bn-A03-p22002284 | 156.958 |
| A03 | Bn-A03-p22299476 | 164.009 |
| A03 | Bn-A03-p22369823 | 164.051 |
| A03 | Bn-A03-p22382828 | 164.207 |
| A03 | Bn-A03-p22349705 | 164.242 |
| A03 | Bn-A03-p22323976 | 164.249 |
| A03 | Bn-A03-p22339476 | 164.29 |
| A03 | Bn-A03-p24092986 | 173.973 |
| A03 | Bn-A03-p23992075 | 174.544 |
| A03 | Bn-A03-p23996628 | 175.01 |
| A03 | Bn-A03-p24104930 | 175.835 |
| A03 | Bn-A03-p24154359 | 177.137 |
| A03 | Bn-A03-p24397912 | 178.283 |
| A03 | Bn-A03-p24396004 | 178.417 |
| A03 | Bn-A03-p24424231 | 178.608 |
| A03 | Bn-A03-p24809290 | 178.659 |
| A03 | Bn-A03-p24394796 | 178.947 |
| A03 | Bn-A03-p25071259 | 179.848 |
| A03 | Bn-A03-p25337594 | 180.399 |
| A03 | Bn-A03-p25330901 | 180.829 |
| A03 | Bn-A03-p25314490 | 181.191 |
| A03 | Bn-A03-p25474850 | 182.036 |
| A03 | Bn-A03-p25489551 | 182.05 |
| A03 | Bn-A03-p25584526 | 184.252 |
| A03 | Bn-A03-p25636300 | 185.35 |
| A03 | Bn-A03-p25668597 | 185.895 |
| A03 | Bn-A03-p25679775 | 186.366 |
| A03 | Bn-A03-p25715049 | 186.878 |
| A03 | Bn-A03-p25734907 | 187.053 |
| A03 | Bn-A03-p25785875 | 187.535 |
| A03 | Bn-A03-p25868537 | 187.991 |
| A03 | Bn-A03-p26071059 | 189.418 |
| A03 | Bn-A03-p26075478 | 189.473 |
| A03 | Bn-A03-p26275444 | 189.482 |
| A03 | Bn-A03-p26121502 | 189.571 |
| A03 | Bn-A03-p26055806 | 189.587 |
| A03 | Bn-A03-p26311193 | 190.123 |
| A03 | Bn-A03-p26444464 | 190.75 |
| A03 | Bn-A03-p26405367 | 190.977 |
| A03 | Bn-A03-p26572622 | 191.156 |
| A04 | Bn-A04-p267294 | 0 |
| A04 | Bn-A04-p459395 | 1.24 |
| A04 | Bn-A04-p487513 | 1.65 |
| A04 | Bn-A04-p463864 | 1.92 |
| A04 | Bn-A04-p645702 | 2.203 |
| A04 | Bn-A04-p354688 | 2.606 |
| A04 | Bn-A04-p397523 | 2.882 |
| A04 | Bn-A04-p375224 | 2.9 |
| A04 | Bn-A04-p757668 | 3.284 |
| A04 | Bn-A04-p641998 | 3.355 |
| A04 | Bn-A04-p570250 | 3.471 |
| A04 | Bn-A04-p599845 | 3.548 |
| A04 | Bn-A04-p619001 | 3.636 |
| A04 | Bn-A04-p560622 | 3.777 |
| A04 | Bn-A04-p556991 | 3.821 |
| A04 | Bn-A04-p538802 | 4.25 |
| A04 | Bn-scaff_16714_1-p112449 | 4.407 |
| A04 | Bn-A04-p1018807 | 5.012 |
| A04 | Bn-A04-p870365 | 5.306 |
| A04 | Bn-A04-p980952 | 5.516 |
| A04 | Bn-A04-p1062119 | 6.853 |
| A04 | Bn-A04-p1274596 | 7.473 |
| A04 | Bn-A04-p1231127 | 7.814 |
| A04 | Bn-A04-p1311567 | 8.147 |
| A04 | Bn-A04-p1346738 | 9.293 |
| A04 | Bn-A04-p1390449 | 9.863 |
| A04 | Bn-A04-p1480226 | 10.144 |
| A04 | Bn-A04-p1585447 | 10.702 |
| A04 | Bn-A04-p1623151 | 11.467 |
| A04 | Bn-A04-p3135512 | 12.536 |
| A04 | Bn-A04-p3126855 | 12.927 |
| A04 | Bn-A04-p3181336 | 13.053 |
| A04 | Bn-A04-p1927138 | 13.522 |
| A04 | Bn-A04-p1900422 | 13.612 |
| A04 | Bn-A04-p1893968 | 13.72 |
| A04 | Bn-A04-p1815404 | 13.964 |
| A04 | Bn-A04-p1759257 | 14.101 |
| A04 | Bn-A04-p1780149 | 14.228 |
| A04 | Bn-A04-p1774926 | 14.425 |
| A04 | Bn-A04-p1895018 | 14.911 |
| A04 | Bn-Scaffold000104-p199071 | 18.615 |
| A04 | Bn-Scaffold000104-p138056 | 19.525 |
| A04 | Bn-Scaffold000104-p137791 | 19.771 |
| A04 | Bn-A04-p5982123 | 20.483 |
| A04 | Bn-A04-p6697580 | 22.088 |
| A04 | Bn-A04-p6463878 | 22.092 |
| A04 | Bn-A04-p8208799 | 26.55 |
| A04 | Bn-A04-p8287776 | 26.966 |
| A04 | Bn-A04-p8310328 | 27.006 |
| A04 | Bn-A04-p8017096 | 27.785 |
| A04 | Bn-A04-p8773241 | 30.247 |
| A04 | Bn-scaff_16694_1-p22548 | 30.577 |
| A04 | Bn-A04-p8796706 | 30.835 |
| A04 | Bn-A04-p8850036 | 30.837 |
| A04 | Bn-A04-p8847485 | 30.838 |
| A04 | Bn-A04-p8830454 | 30.975 |
| A04 | Bn-A04-p9086923 | 31.234 |
| A04 | Bn-A04-p9309213 | 31.681 |
| A04 | Bn-A04-p9426523 | 31.772 |
| A04 | Bn-A04-p9841094 | 33.351 |
| A04 | Bn-A04-p9847875 | 33.565 |
| A04 | Bn-A04-p9844020 | 33.623 |
| A04 | Bn-A04-p9870879 | 33.744 |
| A04 | Bn-A04-p9865712 | 33.947 |
| A04 | Bn-A04-p9930371 | 34.201 |
| A04 | Bn-A04-p9932606 | 34.239 |
| A04 | Bn-A04-p9878192 | 34.643 |
| A04 | Bn-A04-p9878444 | 34.722 |
| A04 | Bn-A04-p10393460 | 35.53 |
| A04 | Bn-A04-p10566018 | 37.482 |
| A04 | Bn-A04-p10719274 | 41.219 |
| A04 | Bn-A04-p10872405 | 41.897 |
| A04 | Bn-A04-p11588227 | 45.972 |
| A04 | Bn-A04-p11420466 | 46.149 |
| A04 | Bn-A04-p12051277 | 49.845 |
| A04 | Bn-A04-p12210199 | 50.824 |
| A04 | Bn-A04-p12236240 | 51.218 |
| A04 | Bn-A04-p12288635 | 51.403 |
| A04 | Bn-A04-p12243311 | 52.804 |
| A04 | Bn-A04-p12793456 | 52.929 |
| A04 | Bn-A04-p12256481 | 53.08 |
| A04 | Bn-A04-p12690759 | 53.088 |
| A04 | Bn-A04-p12659402 | 53.203 |
| A04 | Bn-A04-p12827460 | 53.944 |
| A04 | Bn-A04-p13116453 | 55.805 |
| A04 | Bn-A04-p13096878 | 56.052 |
| A04 | Bn-A04-p13129601 | 56.402 |
| A04 | Bn-A04-p13128840 | 56.549 |
| A04 | Bn-A04-p13368998 | 57.028 |
| A04 | Bn-A04-p13306369 | 57.411 |
| A04 | Bn-A04-p13583325 | 57.581 |
| A04 | Bn-A04-p13269135 | 57.947 |
| A04 | Bn-A04-p13532829 | 58.137 |
| A04 | Bn-A04-p13389622 | 58.609 |
| A04 | Bn-A04-p13336924 | 59.014 |
| A04 | Bn-A04-p13541465 | 59.174 |
| A04 | Bn-A04-p13430818 | 59.44 |
| A04 | Bn-A04-p14159558 | 64.759 |
| A04 | Bn-A04-p14271110 | 66.025 |
| A04 | Bn-A04-p14565883 | 68.509 |
| A04 | Bn-A04-p14397957 | 68.641 |
| A04 | Bn-A04-p14478925 | 69.418 |
| A04 | Bn-A04-p14483727 | 69.581 |
| A04 | Bn-A04-p15009639 | 71.111 |
| A04 | Bn-A04-p14999714 | 71.453 |
| A04 | Bn-A04-p14687930 | 71.754 |
| A04 | Bn-A04-p14673783 | 71.855 |
| A04 | Bn-A04-p14987934 | 72.653 |
| A04 | Bn-A04-p15122412 | 74.6 |
| A04 | Bn-A04-p15298740 | 75.572 |
| A04 | Bn-A04-p15287200 | 75.907 |
| A04 | Bn-A04-p15244369 | 76.146 |
| A04 | Bn-A04-p15195920 | 76.247 |
| A04 | Bn-A04-p15312437 | 76.294 |
| A04 | Bn-A04-p15164920 | 76.52 |
| A04 | Bn-A04-p15411699 | 77.278 |
| A04 | Bn-A04-p15475759 | 78.536 |
| A04 | Bn-A04-p15514869 | 79.295 |
| A04 | Bn-A04-p15710186 | 79.821 |
| A04 | Bn-A04-p15670197 | 80.018 |
| A04 | Bn-A04-p15535011 | 80.866 |
| A04 | Bn-A04-p15534626 | 81.019 |
| A04 | Bn-A04-p16104616 | 82.95 |
| A04 | Bn-A04-p16131108 | 82.959 |
| A04 | Bn-A04-p16233024 | 83.267 |
| A04 | Bn-A04-p16264494 | 83.426 |
| A04 | Bn-A04-p16445602 | 83.747 |
| A04 | Bn-A04-p16523010 | 83.795 |
| A04 | Bn-A04-p16486340 | 83.811 |
| A04 | Bn-A04-p16368852 | 83.957 |
| A04 | Bn-A04-p16744035 | 84.185 |
| A04 | Bn-A04-p16748162 | 84.219 |
| A04 | Bn-A04-p17019529 | 84.23 |
| A04 | Bn-A04-p17052071 | 84.259 |
| A04 | Bn-A04-p16745356 | 84.263 |
| A04 | Bn-A04-p16819445 | 84.406 |
| A04 | Bn-A04-p17058367 | 84.427 |
| A04 | Bn-A01-p18240444 | 85.234 |
| A04 | Bn-A04-p17171349 | 85.306 |
| A04 | Bn-A04-p17219743 | 85.46 |
| A04 | Bn-A04-p17223885 | 85.49 |
| A04 | Bn-A04-p17172246 | 85.678 |
| A04 | Bn-A04-p17281577 | 86.003 |
| A04 | Bn-A04-p17976746 | 89.72 |
| A04 | Bn-A04-p18003170 | 90.433 |
| A04 | Bn-A04-p17858149 | 90.575 |
| A04 | Bn-A04-p17871505 | 90.653 |
| A04 | Bn-A04-p17816796 | 90.805 |
| A04 | Bn-A04-p18417786 | 93.381 |
| A04 | Bn-A04-p18429573 | 94.169 |
| A04 | Bn-A04-p18471424 | 94.424 |
| A04 | Bn-A04-p18457550 | 94.686 |
| A04 | Bn-A04-p18530009 | 95.738 |
| A05 | Bn-A05-p19207735 | 0 |
| A05 | Bn-A05-p19267475 | 0.222 |
| A05 | Bn-A05-p19267153 | 0.57 |
| A05 | Bn-A05-p19250569 | 1.04 |
| A05 | Bn-A05-p19242100 | 1.368 |
| A05 | Bn-A05-p19242397 | 1.849 |
| A05 | Bn-A05-p19238134 | 2.313 |
| A05 | Bn-A05-p19096242 | 2.441 |
| A05 | Bn-A05-p19070214 | 2.739 |
| A05 | Bn-A05-p19031269 | 2.843 |
| A05 | Bn-A05-p18744312 | 3.299 |
| A05 | Bn-A05-p18761878 | 3.41 |
| A05 | Bn-A05-p19116891 | 3.899 |
| A05 | Bn-A05-p19128595 | 4.184 |
| A05 | Bn-A05-p19105857 | 4.804 |
| A05 | Bn-A05-p18768801 | 5.719 |
| A05 | Bn-A05-p18753257 | 5.962 |
| A05 | Bn-A05-p18391581 | 6.28 |
| A05 | Bn-A05-p17739455 | 6.852 |
| A05 | Bn-A05-p20074144 | 7.609 |
| A05 | Bn-A05-p17761769 | 8.077 |
| A05 | Bn-A05-p20127293 | 9.327 |
| A05 | Bn-A05-p20167176 | 9.415 |
| A05 | Bn-A05-p20163118 | 9.857 |
| A05 | Bn-A05-p16836518 | 12.816 |
| A05 | Bn-A05-p17203695 | 13.671 |
| A05 | Bn-A05-p16789633 | 13.854 |
| A05 | Bn-A05-p16846880 | 14.253 |
| A05 | Bn-A05-p16775295 | 14.51 |
| A05 | Bn-A05-p16844504 | 14.582 |
| A05 | Bn-A05-p16811156 | 14.616 |
| A05 | Bn-A05-p16755625 | 14.834 |
| A05 | Bn-A05-p16477848 | 15.44 |
| A05 | Bn-A05-p16664411 | 15.559 |
| A05 | Bn-A05-p16507306 | 15.58 |
| A05 | Bn-A05-p16460167 | 15.582 |
| A05 | Bn-A05-p16544454 | 15.764 |
| A05 | Bn-A05-p16422457 | 15.882 |
| A05 | Bn-A05-p16652401 | 16.014 |
| A05 | Bn-A05-p16581289 | 16.173 |
| A05 | Bn-A05-p16588569 | 16.242 |
| A05 | Bn-A05-p16274076 | 16.698 |
| A05 | Bn-A05-p16359545 | 16.825 |
| A05 | Bn-A05-p15905187 | 17.002 |
| A05 | Bn-A05-p15894915 | 17.293 |
| A05 | Bn-A05-p14830384 | 17.932 |
| A05 | Bn-A05-p14821280 | 18.03 |
| A05 | Bn-A05-p14709370 | 18.231 |
| A05 | Bn-A05-p14723523 | 18.237 |
| A05 | Bn-A05-p14740992 | 18.283 |
| A05 | Bn-A05-p14726690 | 18.289 |
| A05 | Bn-A05-p14747774 | 18.347 |
| A05 | Bn-A05-p14739008 | 18.371 |
| A05 | Bn-A05-p14840113 | 18.415 |
| A05 | Bn-A05-p15169361 | 18.658 |
| A05 | Bn-A05-p15082396 | 18.696 |
| A05 | Bn-A05-p15115420 | 18.821 |
| A05 | Bn-A05-p15169260 | 18.932 |
| A05 | Bn-A05-p15116220 | 18.944 |
| A05 | Bn-A05-p15385848 | 19.135 |
| A05 | Bn-A05-p11384651 | 19.345 |
| A05 | Bn-A05-p11012409 | 19.446 |
| A05 | Bn-A05-p14529716 | 19.581 |
| A05 | Bn-A06-p20320258 | 19.582 |
| A05 | Bn-A05-p14662656 | 19.675 |
| A05 | Bn-Scaffold000324-p11634 | 19.703 |
| A05 | Bn-A05-p12170375 | 19.792 |
| A05 | Bn-A05-p12199879 | 19.882 |
| A05 | Bn-A05-p13301511 | 19.945 |
| A05 | Bn-A05-p14338060 | 19.947 |
| A05 | Bn-A05-p12597194 | 20.006 |
| A05 | Bn-A05-p12119364 | 20.007 |
| A05 | Bn-A05-p12026117 | 20.013 |
| A05 | Bn-A05-p12418828 | 20.014 |
| A05 | Bn-A05-p12019087 | 20.073 |
| A05 | Bn-A05-p14175152 | 20.111 |
| A05 | Bn-A05-p13869168 | 20.112 |
| A05 | Bn-A05-p13000538 | 20.132 |
| A05 | Bn-A05-p13823408 | 20.134 |
| A05 | Bn-A05-p13774122 | 20.14 |
| A05 | Bn-A05-p11686214 | 20.377 |
| A05 | Bn-A05-p11088814 | 20.5 |
| A05 | Bn-A05-p6955996 | 20.755 |
| A05 | Bn-A05-p11151246 | 20.848 |
| A05 | Bn-A05-p11225095 | 21.013 |
| A05 | Bn-A05-p11255103 | 21.032 |
| A05 | Bn-A05-p11690317 | 21.175 |
| A05 | Bn-A10-p923788 | 21.453 |
| A05 | Bn-A05-p10951975 | 21.737 |
| A05 | Bn-A05-p10966950 | 21.901 |
| A05 | Bn-A05-p10948934 | 21.958 |
| A05 | Bn-A05-p10930228 | 22.095 |
| A05 | Bn-A05-p10719967 | 22.408 |
| A05 | Bn-A05-p10783057 | 22.457 |
| A05 | Bn-A05-p10367279 | 22.724 |
| A05 | Bn-Scaffold000218-p18252 | 24.114 |
| A05 | Bn-A08-p1308299 | 24.148 |
| A05 | Bn-A08-p1364252 | 24.395 |
| A05 | Bn-A08-p1332628 | 24.396 |
| A05 | Bn-A08-p1336421 | 24.467 |
| A05 | Bn-A08-p1369206 | 24.63 |
| A05 | Bn-A05-p10009197 | 24.866 |
| A05 | Bn-A05-p9758517 | 25.098 |
| A05 | Bn-A05-p9849822 | 25.181 |
| A05 | Bn-A05-p9890807 | 25.377 |
| A05 | Bn-A05-p9765872 | 25.605 |
| A05 | Bn-A01-p11993589 | 26.16 |
| A05 | Bn-A05-p20314128 | 28.034 |
| A05 | Bn-A05-p9766332 | 28.116 |
| A05 | Bn-A05-p7903619 | 28.284 |
| A05 | Bn-A05-p7729367 | 28.479 |
| A05 | Bn-A05-p7651411 | 28.916 |
| A05 | Bn-A05-p20487983 | 30.978 |
| A05 | Bn-A05-p20485998 | 31.214 |
| A05 | Bn-A05-p20673293 | 31.827 |
| A05 | Bn-A05-p20594196 | 32.032 |
| A05 | Bn-A05-p20591964 | 32.319 |
| A05 | Bn-A05-p20719343 | 32.582 |
| A05 | Bn-A05-p20692714 | 32.668 |
| A05 | Bn-A05-p20632153 | 32.76 |
| A05 | Bn-A05-p20623573 | 33.254 |
| A05 | Bn-A05-p7098949 | 35.098 |
| A05 | Bn-A05-p6995896 | 36.182 |
| A05 | Bn-A05-p6951707 | 36.368 |
| A05 | Bn-A05-p6957948 | 36.705 |
| A05 | Bn-A05-p6748150 | 37.458 |
| A05 | Bn-A05-p6688695 | 38.436 |
| A05 | Bn-A05-p22826924 | 43.529 |
| A05 | Bn-A05-p22789344 | 44.525 |
| A05 | Bn-A05-p22392750 | 45.343 |
| A05 | Bn-A05-p22454211 | 46.177 |
| A05 | Bn-A05-p22028401 | 49.268 |
| A05 | Bn-A05-p22021177 | 49.396 |
| A05 | Bn-A05-p21886422 | 49.877 |
| A05 | Bn-A05-p21889289 | 50.46 |
| A06 | Bn-A06-p55225 | 0 |
| A06 | Bn-A06-p149145 | 1.39 |
| A06 | Bn-A06-p137614 | 2.836 |
| A06 | Bn-A06-p172171 | 3.137 |
| A06 | Bn-A06-p172731 | 3.247 |
| A06 | Bn-A06-p709547 | 5.76 |
| A06 | Bn-A06-p781674 | 6.414 |
| A06 | Bn-A06-p899811 | 7.314 |
| A06 | Bn-A06-p1016417 | 8.094 |
| A06 | Bn-A06-p1149034 | 9.082 |
| A06 | Bn-A06-p1116930 | 9.224 |
| A06 | Bn-A06-p1119751 | 9.383 |
| A06 | Bn-A06-p1185371 | 9.65 |
| A06 | Bn-A06-p1173126 | 9.772 |
| A06 | Bn-A06-p1083892 | 9.931 |
| A06 | Bn-A06-p1106605 | 10.059 |
| A06 | Bn-A06-p1119681 | 10.244 |
| A06 | Bn-A06-p1345642 | 10.956 |
| A06 | Bn-A06-p1358955 | 11.448 |
| A06 | Bn-A06-p1348404 | 11.488 |
| A06 | Bn-A06-p1330567 | 11.636 |
| A06 | Bn-A06-p1404859 | 11.816 |
| A06 | Bn-A06-p1776668 | 12.497 |
| A06 | Bn-A06-p1768254 | 12.833 |
| A06 | Bn-A06-p1650529 | 13.153 |
| A06 | Bn-A06-p1722627 | 13.18 |
| A06 | Bn-A06-p1652088 | 13.294 |
| A06 | Bn-A06-p1626020 | 13.435 |
| A06 | Bn-A06-p1768918 | 13.516 |
| A06 | Bn-A06-p1661477 | 13.692 |
| A06 | Bn-A06-p1800467 | 14.372 |
| A06 | Bn-A06-p1925858 | 17.453 |
| A06 | Bn-A06-p2390609 | 22.783 |
| A06 | Bn-A06-p2345966 | 23.525 |
| A06 | Bn-A06-p2407080 | 24.015 |
| A06 | Bn-A06-p2413971 | 24.613 |
| A06 | Bn-A06-p2521454 | 25.481 |
| A06 | Bn-A06-p2437284 | 26.328 |
| A06 | Bn-A06-p2586825 | 26.56 |
| A06 | Bn-A06-p2619089 | 26.993 |
| A06 | Bn-A06-p2756247 | 28.583 |
| A06 | Bn-A06-p2718852 | 29.083 |
| A06 | Bn-A06-p2717967 | 29.452 |
| A06 | Bn-A06-p3051928 | 35.683 |
| A06 | Bn-A06-p3118627 | 36.457 |
| A06 | Bn-A06-p3207623 | 37.235 |
| A06 | Bn-A06-p3256782 | 37.51 |
| A06 | Bn-A06-p3434498 | 37.919 |
| A06 | Bn-A06-p3532353 | 38.496 |
| A06 | Bn-A06-p3444868 | 38.718 |
| A06 | Bn-A06-p3361377 | 38.972 |
| A06 | Bn-A06-p3584489 | 39.236 |
| A06 | Bn-A06-p3834324 | 40.258 |
| A06 | Bn-A06-p3867353 | 41.811 |
| A06 | Bn-A06-p3992289 | 43.288 |
| A06 | Bn-A06-p4013010 | 43.625 |
| A06 | Bn-A06-p4211266 | 44.676 |
| A06 | Bn-A06-p4179318 | 45.048 |
| A06 | Bn-A06-p4196008 | 45.274 |
| A06 | Bn-A06-p4461241 | 46.522 |
| A06 | Bn-A06-p4333144 | 47.374 |
| A06 | Bn-A06-p4337931 | 47.441 |
| A06 | Bn-A06-p4362700 | 47.451 |
| A06 | Bn-A06-p4340819 | 47.555 |
| A06 | Bn-A06-p4333227 | 47.955 |
| A06 | Bn-A06-p4436168 | 48.918 |
| A06 | Bn-A06-p5020498 | 53.099 |
| A06 | Bn-A06-p5020675 | 53.421 |
| A06 | Bn-A06-p4933932 | 54.492 |
| A06 | Bn-A06-p6679810 | 58.201 |
| A06 | Bn-A06-p6183859 | 59.62 |
| A06 | Bn-A06-p6520301 | 59.621 |
| A06 | Bn-A06-p6403382 | 60.406 |
| A06 | Bn-A06-p6761241 | 62.254 |
| A06 | Bn-A06-p6980159 | 62.621 |
| A06 | Bn-A06-p6847367 | 62.872 |
| A06 | Bn-A06-p7187455 | 63.528 |
| A06 | Bn-A06-p7279909 | 63.962 |
| A06 | Bn-A06-p7230364 | 64.051 |
| A06 | Bn-A06-p7658535 | 65.248 |
| A06 | Bn-A06-p7665759 | 65.381 |
| A06 | Bn-A06-p7626450 | 65.507 |
| A06 | Bn-A06-p7584691 | 65.932 |
| A06 | Bn-A06-p7726350 | 66.027 |
| A06 | Bn-A06-p9074653 | 66.65 |
| A06 | Bn-A06-p8263325 | 66.875 |
| A06 | Bn-A06-p8426071 | 67.039 |
| A06 | Bn-A06-p8402978 | 67.049 |
| A06 | Bn-A06-p8871997 | 67.112 |
| A06 | Bn-A06-p8090520 | 67.277 |
| A06 | Bn-A06-p8766088 | 67.295 |
| A06 | Bn-A06-p8546149 | 67.297 |
| A06 | Bn-A06-p8614872 | 67.339 |
| A06 | Bn-A06-p8634648 | 67.388 |
| A06 | Bn-A06-p7437586 | 68.504 |
| A06 | Bn-A06-p7499751 | 69.007 |
| A06 | Bn-A06-p7707699 | 69.123 |
| A06 | Bn-A06-p6995492 | 69.758 |
| A06 | Bn-A06-p6959460 | 69.942 |
| A06 | Bn-A06-p6747460 | 70.633 |
| A06 | Bn-A06-p14546946 | 77.025 |
| A06 | Bn-A06-p14864165 | 77.759 |
| A06 | Bn-A06-p14813811 | 78.055 |
| A06 | Bn-A06-p16460575 | 82.337 |
| A06 | Bn-A06-p16553640 | 83.201 |
| A06 | Bn-A06-p16678748 | 84.966 |
| A06 | Bn-A06-p16718752 | 85.306 |
| A06 | Bn-A06-p17033313 | 85.825 |
| A06 | Bn-A06-p17114458 | 86.009 |
| A06 | Bn-A06-p16645035 | 86.329 |
| A06 | Bn-A06-p16682224 | 86.336 |
| A06 | Bn-A06-p16840167 | 86.477 |
| A06 | Bn-A06-p16689717 | 86.513 |
| A06 | Bn-A06-p17220153 | 86.92 |
| A06 | Bn-A06-p17208027 | 87.238 |
| A06 | Bn-A06-p17176086 | 87.42 |
| A06 | Bn-A06-p17321987 | 87.641 |
| A06 | Bn-A06-p17226648 | 88.015 |
| A06 | Bn-A06-p17549603 | 88.9 |
| A06 | Bn-A06-p17549347 | 89.285 |
| A06 | Bn-scaff_15705_1-p780228 | 89.569 |
| A06 | Bn-A06-p17659127 | 91.542 |
| A06 | Bn-A06-p17939632 | 93.452 |
| A06 | Bn-A06-p18000403 | 93.527 |
| A06 | Bn-A06-p18033006 | 93.548 |
| A06 | Bn-A06-p18028791 | 93.575 |
| A06 | Bn-A06-p18028879 | 93.589 |
| A06 | Bn-A06-p18059678 | 93.907 |
| A06 | Bn-A06-p18102164 | 94.128 |
| A06 | Bn-A06-p18152497 | 94.818 |
| A06 | Bn-A06-p18207917 | 95.187 |
| A06 | Bn-A06-p18247490 | 96.282 |
| A06 | Bn-A06-p18432812 | 97.703 |
| A06 | Bn-A06-p22608192 | 113.463 |
| A06 | Bn-A06-p22644580 | 116.211 |
| A06 | Bn-A06-p22788890 | 118.394 |
| A06 | Bn-A06-p22781886 | 118.483 |
| A06 | Bn-A06-p22806976 | 118.819 |
| A06 | Bn-A06-p22835281 | 118.838 |
| A06 | Bn-A06-p22816078 | 118.84 |
| A06 | Bn-A06-p22921908 | 119.05 |
| A06 | Bn-A06-p22809449 | 119.116 |
| A06 | Bn-A06-p22782335 | 119.288 |
| A06 | Bn-A06-p22786948 | 119.434 |
| A06 | Bn-A06-p22914117 | 119.786 |
| A06 | Bn-A06-p22913862 | 119.995 |
| A06 | Bn-A06-p23350246 | 122.161 |
| A06 | Bn-A06-p23591616 | 122.625 |
| A06 | Bn-A06-p23612730 | 123.513 |
| A06 | Bn-A06-p23681386 | 126.481 |
| A06 | Bn-A06-p23706979 | 126.634 |
| A06 | Bn-A06-p23944667 | 127.603 |
| A06 | Bn-A06-p23806343 | 127.678 |
| A06 | Bn-A06-p23968879 | 127.82 |
| A06 | Bn-A06-p23750653 | 128.137 |
| A06 | Bn-A06-p24075375 | 128.539 |
| A06 | Bn-A06-p24099288 | 129.425 |
| A06 | Bn-A06-p24006085 | 129.582 |
| A06 | Bn-A06-p24278510 | 133.325 |
| A06 | Bn-A06-p24274213 | 133.715 |
| A06 | Bn-A06-p24267499 | 134.132 |
| A06 | Bn-A06-p24267745 | 134.357 |
| A06 | Bn-A06-p24814906 | 137.135 |
| A06 | Bn-A06-p24713872 | 137.509 |
| A06 | Bn-A06-p24744523 | 138.095 |
| A06 | Bn-A06-p24717767 | 138.538 |
| A06 | Bn-A06-p25878721 | 144.951 |
| A06 | Bn-A06-p26155068 | 150.009 |
| A06 | Bn-A06-p26173206 | 150.7 |
| A06 | Bn-A06-p26222718 | 151.436 |
| A07 | Bn-A07-p3275876 | 0 |
| A07 | Bn-A07-p2698690 | 0.117 |
| A07 | Bn-A07-p3262752 | 0.126 |
| A07 | Bn-A10-p3243369 | 0.13 |
| A07 | Bn-A07-p3216267 | 0.532 |
| A07 | Bn-A07-p3028546 | 0.536 |
| A07 | Bn-scaff_16721_1-p401767 | 0.615 |
| A07 | Bn-A07-p3041012 | 0.848 |
| A07 | Bn-A07-p2980414 | 0.894 |
| A07 | Bn-A07-p3538601 | 2.127 |
| A07 | Bn-A07-p3659345 | 2.955 |
| A07 | Bn-A07-p3599603 | 3.16 |
| A07 | Bn-A07-p4050475 | 3.793 |
| A07 | Bn-A07-p4387388 | 3.941 |
| A07 | Bn-A07-p4815239 | 4.077 |
| A07 | Bn-A07-p4414432 | 4.314 |
| A07 | Bn-scaff_17584_1-p683277 | 4.37 |
| A07 | Bn-A01-p10526561 | 4.463 |
| A07 | Bn-A01-p10550486 | 4.497 |
| A07 | Bn-scaff_17584_1-p223738 | 4.556 |
| A07 | Bn-A07-p4912709 | 4.772 |
| A07 | Bn-A07-p5020495 | 4.824 |
| A07 | Bn-A07-p4687740 | 4.885 |
| A07 | Bn-A07-p5095657 | 4.933 |
| A07 | Bn-A07-p5412930 | 5.03 |
| A07 | Bn-A07-p4151821 | 5.314 |
| A07 | Bn-A07-p4155337 | 5.331 |
| A07 | Bn-A07-p4191541 | 5.445 |
| A07 | Bn-A07-p4173583 | 5.517 |
| A07 | Bn-A07-p4109343 | 5.551 |
| A07 | Bn-A07-p4109459 | 5.69 |
| A07 | Bn-A07-p5234114 | 5.835 |
| A07 | Bn-A07-p3717215 | 6.038 |
| A07 | Bn-A07-p4709589 | 6.196 |
| A07 | Bn-A07-p3746131 | 6.42 |
| A07 | Bn-A07-p4142320 | 6.437 |
| A07 | Bn-A07-p3609912 | 6.522 |
| A07 | Bn-A07-p4309577 | 6.688 |
| A07 | Bn-A07-p4204647 | 6.769 |
| A07 | Bn-A07-p4307671 | 6.873 |
| A07 | Bn-A07-p5449174 | 7.109 |
| A07 | Bn-A07-p4898790 | 7.306 |
| A07 | Bn-A07-p4914940 | 7.467 |
| A07 | Bn-A07-p4777275 | 7.655 |
| A07 | Bn-A07-p4535707 | 7.925 |
| A07 | Bn-A07-p4937244 | 8.263 |
| A07 | Bn-A07-p4568148 | 8.297 |
| A07 | Bn-A07-p9639704 | 9.954 |
| A07 | Bn-A07-p9020570 | 11.121 |
| A07 | Bn-A07-p9071682 | 11.502 |
| A07 | Bn-A07-p9493282 | 12.166 |
| A07 | Bn-A07-p8935344 | 12.168 |
| A07 | Bn-A07-p9089710 | 12.566 |
| A07 | Bn-A07-p9041783 | 12.814 |
| A07 | Bn-A07-p9521819 | 13.319 |
| A07 | Bn-A07-p9321347 | 13.38 |
| A07 | Bn-A03-p5912430 | 13.425 |
| A07 | Bn-A07-p9441979 | 14.343 |
| A07 | Bn-A07-p9764930 | 14.515 |
| A07 | Bn-A07-p8854569 | 15.144 |
| A07 | Bn-A07-p7117229 | 15.653 |
| A07 | Bn-A07-p7117228 | 15.66 |
| A07 | Bn-A07-p9661444 | 15.8 |
| A07 | Bn-A07-p7139702 | 17.01 |
| A07 | Bn-A07-p7146249 | 17.122 |
| A07 | Bn-A07-p7297758 | 18.637 |
| A07 | Bn-A07-p7298603 | 18.893 |
| A07 | Bn-scaff_22536_1-p164551 | 19.106 |
| A07 | Bn-A07-p7309287 | 19.27 |
| A07 | Bn-A07-p7380906 | 19.274 |
| A07 | Bn-A07-p7302034 | 19.317 |
| A07 | Bn-A07-p7434126 | 19.629 |
| A07 | Bn-A07-p7367247 | 19.664 |
| A07 | Bn-A07-p7499400 | 19.798 |
| A07 | Bn-A07-p7562328 | 20.214 |
| A07 | Bn-A07-p7528095 | 20.228 |
| A07 | Bn-A07-p7606228 | 20.778 |
| A07 | Bn-A07-p7678712 | 20.963 |
| A07 | Bn-A07-p7793608 | 21.588 |
| A07 | Bn-A07-p7605204 | 22.069 |
| A07 | Bn-A07-p7740173 | 22.417 |
| A07 | Bn-A07-p8451913 | 24.694 |
| A07 | Bn-A07-p8696690 | 24.875 |
| A07 | Bn-A07-p8668003 | 25.071 |
| A07 | Bn-A07-p8718607 | 25.472 |
| A07 | Bn-A07-p8571463 | 25.709 |
| A07 | Bn-A07-p8598343 | 25.809 |
| A07 | Bn-A07-p8593730 | 26.092 |
| A07 | Bn-A07-p8790021 | 26.386 |
| A07 | Bn-A07-p8724769 | 26.684 |
| A07 | Bn-A07-p7009935 | 28.797 |
| A07 | Bn-A07-p7039686 | 28.88 |
| A07 | Bn-A07-p7246175 | 29.623 |
| A07 | Bn-A07-p6244683 | 30.319 |
| A07 | Bn-A07-p6576078 | 30.915 |
| A07 | Bn-scaff_28429_1-p325716 | 31.46 |
| A07 | Bn-A07-p6898363 | 32.392 |
| A07 | Bn-A07-p12397651 | 39.255 |
| A07 | Bn-A07-p12486479 | 39.887 |
| A07 | Bn-A07-p12576869 | 40.789 |
| A07 | Bn-A07-p12705404 | 41.258 |
| A07 | Bn-A07-p12594658 | 41.968 |
| A07 | Bn-A07-p12502804 | 42.801 |
| A07 | Bn-A07-p13192299 | 43.57 |
| A07 | Bn-scaff_15763_1-p1049180 | 44.137 |
| A07 | Bn-A07-p13167740 | 44.6 |
| A07 | Bn-A07-p12708718 | 45.334 |
| A07 | Bn-A07-p13283299 | 46.138 |
| A07 | Bn-A07-p13202840 | 46.42 |
| A07 | Bn-A07-p9897363 | 47.681 |
| A07 | Bn-A07-p14029820 | 49.429 |
| A07 | Bn-A07-p14121891 | 49.643 |
| A07 | Bn-A07-p13964923 | 50.73 |
| A07 | Bn-A07-p13971525 | 50.754 |
| A07 | Bn-A07-p14333680 | 51.022 |
| A07 | Bn-A07-p14142383 | 51.236 |
| A07 | Bn-A07-p14462380 | 52.278 |
| A07 | Bn-A07-p14472064 | 52.456 |
| A07 | Bn-A07-p14533453 | 53.057 |
| A07 | Bn-A07-p16871286 | 61.174 |
| A07 | Bn-A07-p16574221 | 61.614 |
| A07 | Bn-A07-p16519850 | 61.635 |
| A07 | Bn-A07-p16553147 | 61.749 |
| A07 | Bn-A07-p16527225 | 61.789 |
| A07 | Bn-A07-p16461287 | 61.802 |
| A07 | Bn-A07-p10003378 | 62.952 |
| A07 | Bn-A07-p16680070 | 63.053 |
| A07 | Bn-A07-p16687846 | 63.135 |
| A07 | Bn-A07-p16697491 | 63.264 |
| A07 | Bn-A07-p9916502 | 63.4 |
| A07 | Bn-A07-p16729233 | 63.565 |
| A07 | Bn-A07-p16717729 | 63.712 |
| A07 | Bn-A07-p16722383 | 63.725 |
| A07 | Bn-A07-p16727616 | 63.735 |
| A07 | Bn-A07-p16726568 | 63.947 |
| A07 | Bn-A07-p10207855 | 64.262 |
| A07 | Bn-A07-p10486045 | 64.428 |
| A07 | Bn-A07-p10204148 | 64.606 |
| A07 | Bn-A07-p10204886 | 64.782 |
| A07 | Bn-A07-p10253702 | 65.151 |
| A07 | Bn-A07-p10236552 | 65.436 |
| A07 | Bn-A07-p10233337 | 65.609 |
| A07 | Bn-A07-p10209077 | 65.923 |
| A07 | Bn-A07-p10398999 | 66.188 |
| A07 | Bn-A07-p10284031 | 67.135 |
| A07 | Bn-A07-p10337326 | 68.984 |
| A07 | Bn-A07-p10494571 | 69.159 |
| A07 | Bn-A07-p10501168 | 69.355 |
| A07 | Bn-A07-p12297809 | 70.32 |
| A07 | Bn-A07-p11951236 | 70.611 |
| A07 | Bn-A07-p12102140 | 70.673 |
| A07 | Bn-A07-p12050100 | 71.042 |
| A07 | Bn-A07-p11861194 | 71.266 |
| A07 | Bn-A01-p17052809 | 71.637 |
| A07 | Bn-A07-p11980798 | 71.811 |
| A07 | Bn-A07-p11724009 | 73.469 |
| A07 | Bn-A07-p11765232 | 73.647 |
| A07 | Bn-A07-p11603894 | 74.215 |
| A07 | Bn-A07-p11627477 | 74.534 |
| A07 | Bn-A07-p18230931 | 76.168 |
| A07 | Bn-A07-p18226326 | 76.474 |
| A07 | Bn-A07-p18308378 | 76.5 |
| A07 | Bn-A07-p18103505 | 76.7 |
| A07 | Bn-A07-p17869552 | 76.996 |
| A07 | Bn-A07-p18223256 | 77.52 |
| A07 | Bn-A07-p18115747 | 77.676 |
| A07 | Bn-A07-p18227328 | 78.066 |
| A07 | Bn-A07-p18208846 | 78.157 |
| A07 | Bn-A07-p17846018 | 78.428 |
| A07 | Bn-A07-p18914294 | 79.013 |
| A07 | Bn-A07-p18231736 | 79.949 |
| A07 | Bn-A07-p18990899 | 80.456 |
| A07 | Bn-A07-p18922121 | 82.013 |
| A07 | Bn-A07-p18955989 | 82.228 |
| A07 | Bn-A07-p19090612 | 82.734 |
| A07 | Bn-A07-p19009397 | 83.113 |
| A07 | Bn-A07-p19512603 | 83.509 |
| A07 | Bn-A07-p19486760 | 83.773 |
| A07 | Bn-A07-p19190510 | 85.12 |
| A07 | Bn-A07-p19515708 | 85.529 |
| A07 | Bn-A07-p19563230 | 85.58 |
| A07 | Bn-A07-p19192373 | 85.901 |
| A07 | Bn-A07-p19118066 | 86.134 |
| A07 | Bn-A07-p19142808 | 86.249 |
| A07 | Bn-A07-p19111807 | 86.505 |
| A07 | Bn-A07-p19109981 | 86.711 |
| A07 | Bn-A07-p19995985 | 89.836 |
| A07 | Bn-A07-p19871923 | 90.551 |
| A07 | Bn-A07-p19865918 | 90.608 |
| A07 | Bn-A07-p20044413 | 90.612 |
| A07 | Bn-A07-p19915062 | 90.753 |
| A07 | Bn-A07-p20485039 | 96.18 |
| A07 | Bn-A07-p21006643 | 98.9 |
| A07 | Bn-A07-p21328168 | 100.007 |
| A07 | Bn-A07-p21347324 | 100.498 |
| A07 | Bn-A07-p21377298 | 100.538 |
| A07 | Bn-A07-p21400508 | 100.87 |
| A07 | Bn-A07-p21193584 | 101.265 |
| A07 | Bn-A07-p21365616 | 101.271 |
| A07 | Bn-A07-p21449796 | 101.64 |
| A07 | Bn-A07-p21410292 | 101.787 |
| A07 | Bn-A07-p21601441 | 103.91 |
| A07 | Bn-A07-p21803967 | 105.344 |
| A07 | Bn-A07-p21916201 | 105.978 |
| A07 | Bn-A07-p21907519 | 106.199 |
| A07 | Bn-A07-p21918901 | 106.238 |
| A07 | Bn-A07-p21965076 | 106.38 |
| A07 | Bn-A07-p22253687 | 110.433 |
| A07 | Bn-A07-p22329359 | 111.476 |
| A07 | Bn-A07-p22309060 | 112.009 |
| A07 | Bn-A07-p22309183 | 112.143 |
| A07 | Bn-A07-p22398500 | 112.526 |
| A07 | Bn-A07-p22362597 | 112.737 |
| A08 | Bn-A08-p16673732 | 0 |
| A08 | Bn-A08-p16299351 | 0.615 |
| A08 | Bn-A08-p16328281 | 0.981 |
| A08 | Bn-A08-p16412952 | 1.907 |
| A08 | Bn-A08-p16562035 | 2.498 |
| A08 | Bn-A08-p16313016 | 2.784 |
| A08 | Bn-A08-p16653908 | 3.676 |
| A08 | Bn-A08-p16771030 | 4.101 |
| A08 | Bn-A08-p16859028 | 5.004 |
| A08 | Bn-A08-p16846907 | 5.44 |
| A08 | Bn-A08-p16943263 | 7.996 |
| A08 | Bn-A08-p17070496 | 8.509 |
| A08 | Bn-A08-p17202745 | 10.402 |
| A08 | Bn-A08-p17297704 | 11.156 |
| A08 | Bn-A08-p17351964 | 12.049 |
| A08 | Bn-A08-p17355609 | 12.081 |
| A08 | Bn-A08-p17354167 | 12.095 |
| A08 | Bn-A08-p17393018 | 12.255 |
| A08 | Bn-A08-p17402165 | 12.975 |
| A08 | Bn-A08-p17721455 | 13.954 |
| A08 | Bn-A08-p17710867 | 14.012 |
| A08 | Bn-A08-p17647291 | 14.267 |
| A08 | Bn-A08-p17654459 | 14.409 |
| A08 | Bn-A08-p17578990 | 14.543 |
| A08 | Bn-A08-p17816612 | 15.034 |
| A08 | Bn-A08-p17513789 | 15.87 |
| A08 | Bn-A08-p17686371 | 16.244 |
| A08 | Bn-A08-p18000876 | 17.126 |
| A08 | Bn-A08-p18070928 | 17.808 |
| A08 | Bn-A08-p18200896 | 18.584 |
| A08 | Bn-A08-p18153504 | 18.978 |
| A08 | Bn-A08-p18291268 | 20.906 |
| A08 | Bn-A08-p18375745 | 21.338 |
| A08 | Bn-A08-p18356257 | 21.347 |
| A08 | Bn-A08-p18331012 | 21.373 |
| A08 | Bn-A08-p18432919 | 22.107 |
| A08 | Bn-A08-p18462668 | 22.152 |
| A08 | Bn-A08-p18524830 | 22.209 |
| A08 | Bn-A08-p18526873 | 22.508 |
| A08 | Bn-A08-p18543984 | 22.808 |
| A08 | Bn-A08-p18538166 | 23.472 |
| A08 | Bn-A08-p18597209 | 24.381 |
| A08 | Bn-scaff_15923_1-p1096132 | 25.394 |
| A08 | Bn-A08-p19261458 | 31.002 |
| A08 | Bn-A08-p19428204 | 31.548 |
| A08 | Bn-A08-p19452520 | 32.193 |
| A08 | Bn-A08-p19449546 | 32.919 |
| A08 | Bn-A08-p19346369 | 33.021 |
| A09 | Bn-Scaffold000321-p23083 | 0 |
| A09 | Bn-A09-p5189883 | 0.005 |
| A09 | Bn-A09-p5327731 | 0.933 |
| A09 | Bn-A09-p5348717 | 0.974 |
| A09 | Bn-A09-p5958009 | 5.092 |
| A09 | Bn-A09-p7397116 | 5.574 |
| A09 | Bn-A01-p10166739 | 6.332 |
| A09 | Bn-A09-p7047022 | 8.59 |
| A09 | Bn-A09-p9135388 | 18.297 |
| A09 | Bn-A09-p9063447 | 18.613 |
| A09 | Bn-A09-p10212674 | 23.448 |
| A09 | Bn-A09-p10172534 | 23.746 |
| A09 | Bn-A09-p20569958 | 33.693 |
| A09 | Bn-A09-p23331135 | 39.038 |
| A09 | Bn-A09-p23330048 | 39.225 |
| A09 | Bn-A09-p23331059 | 39.463 |
| A09 | Bn-A09-p23356793 | 39.759 |
| A09 | Bn-A09-p23345719 | 41.609 |
| A09 | Bn-A05-p15808942 | 49.41 |
| A09 | Bn-A05-p15789974 | 49.69 |
| A09 | Bn-A09-p24720890 | 49.996 |
| A09 | Bn-A09-p25004061 | 50.593 |
| A09 | Bn-Scaffold000460-p4184 | 51.048 |
| A09 | Bn-A09-p25236454 | 51.2 |
| A09 | Bn-scaff_16770_1-p1969319 | 51.258 |
| A09 | Bn-A09-p25155338 | 51.352 |
| A09 | Bn-A09-p25491749 | 52.599 |
| A09 | Bn-A09-p25493173 | 52.702 |
| A09 | Bn-A09-p25738298 | 53.163 |
| A09 | Bn-A09-p25637924 | 53.28 |
| A09 | Bn-A09-p25536905 | 53.416 |
| A09 | Bn-A09-p25636375 | 53.419 |
| A09 | Bn-A09-p25635243 | 53.475 |
| A09 | Bn-A09-p25739064 | 54.03 |
| A09 | Bn-A09-p25779397 | 54.749 |
| A09 | Bn-A09-p25942252 | 55.281 |
| A09 | Bn-A09-p26346701 | 55.407 |
| A09 | Bn-A09-p26101323 | 55.956 |
| A09 | Bn-A09-p26246760 | 55.976 |
| A09 | Bn-A09-p26517614 | 55.995 |
| A09 | Bn-A09-p26461272 | 57.578 |
| A09 | Bn-A09-p28670849 | 72.081 |
| A09 | Bn-A09-p28654058 | 72.374 |
| A09 | Bn-A09-p28717690 | 73.066 |
| A09 | Bn-A09-p28758854 | 73.911 |
| A09 | Bn-A09-p31402193 | 89.448 |
| A09 | Bn-A09-p31427478 | 89.753 |
| A09 | Bn-A09-p31613718 | 91.212 |
| A09 | Bn-A09-p31611279 | 91.267 |
| A09 | Bn-A09-p31629427 | 91.612 |
| A09 | Bn-A09-p5037318 | 103.754 |
| A09 | Bn-A09-p32027483 | 104.848 |
| A09 | Bn-A09-p32167322 | 105.242 |
| A09 | Bn-A09-p32275168 | 105.855 |
| A09 | Bn-A09-p32321534 | 106.702 |
| A09 | Bn-A09-p32270596 | 106.877 |
| A09 | Bn-A09-p32257776 | 107.223 |
| A09 | Bn-A09-p32258181 | 107.232 |
| A09 | Bn-A09-p32441297 | 108.21 |
| A09 | Bn-A09-p32669666 | 108.949 |
| A09 | Bn-A09-p32959480 | 110.483 |
| A09 | Bn-A09-p33061472 | 110.807 |
| A09 | Bn-A09-p33348598 | 111.648 |
| A09 | Bn-A09-p33358168 | 111.664 |
| A09 | Bn-A09-p33358356 | 111.684 |
| A09 | Bn-A09-p33428597 | 112.754 |
| A09 | Bn-A09-p33427256 | 113.014 |
| A09 | Bn-A09-p33542334 | 113.885 |
| A09 | Bn-A09-p33971771 | 114.908 |
| A09 | Bn-A09-p33972258 | 115.075 |
| A09 | Bn-A09-p34995922 | 123.972 |
| A09 | Bn-A09-p35162641 | 125.005 |
| A09 | Bn-A09-p35484843 | 126.995 |
| A09 | Bn-A09-p35508955 | 128.622 |
| A09 | Bn-scaff_21269_1-p285510 | 128.729 |
| A09 | Bn-A09-p35483201 | 129.203 |
| A09 | Bn-A09-p36462450 | 135.579 |
| A09 | Bn-A09-p36290340 | 136.978 |
| A09 | Bn-A09-p36558536 | 139.028 |
| A10 | Bn-A10-p6344414 | 0 |
| A10 | Bn-A10-p6278596 | 0.361 |
| A10 | Bn-A10-p6299839 | 0.494 |
| A10 | Bn-A10-p6271776 | 0.715 |
| A10 | Bn-A10-p6166631 | 0.792 |
| A10 | Bn-A10-p6148523 | 1.212 |
| A10 | Bn-A10-p5662796 | 1.788 |
| A10 | Bn-A10-p5342414 | 2.152 |
| A10 | Bn-A10-p5224680 | 2.257 |
| A10 | Bn-A10-p5679914 | 2.265 |
| A10 | Bn-A10-p5143614 | 2.33 |
| A10 | Bn-A10-p5239454 | 2.449 |
| A10 | Bn-A10-p5242221 | 2.47 |
| A10 | Bn-A10-p5762378 | 2.957 |
| A10 | Bn-A10-p6176593 | 3.159 |
| A10 | Bn-scaff_20619_1-p316049 | 3.475 |
| A10 | Bn-A10-p5721147 | 3.524 |
| A10 | Bn-A10-p5781113 | 3.943 |
| A10 | Bn-A10-p592763 | 4.723 |
| A10 | Bn-A10-p9973634 | 16.449 |
| A10 | Bn-A10-p9933632 | 16.555 |
| A10 | Bn-A10-p10005671 | 16.589 |
| A10 | Bn-A10-p10070473 | 17.696 |
| A10 | Bn-A10-p10020060 | 17.899 |
| A10 | Bn-A10-p10298013 | 19.014 |
| A10 | Bn-A10-p10464159 | 19.778 |
| A10 | Bn-A10-p10508795 | 20.226 |
| A10 | Bn-A10-p10509825 | 20.678 |
| A10 | Bn-A10-p10613361 | 21.665 |
| A10 | Bn-A10-p10562649 | 21.847 |
| A10 | Bn-A10-p10651432 | 22.515 |
| A10 | Bn-A10-p10703469 | 23.051 |
| A10 | Bn-A10-p10672457 | 23.194 |
| A10 | Bn-A10-p10672359 | 23.454 |
| A10 | Bn-A10-p10725779 | 25.022 |
| A10 | Bn-A10-p10891069 | 25.752 |
| A10 | Bn-A10-p10830979 | 26.388 |
| A10 | Bn-A10-p10954176 | 27.403 |
| A10 | Bn-A10-p10982591 | 27.86 |
| A10 | Bn-A10-p11131283 | 28.835 |
| A10 | Bn-A10-p11075240 | 29.694 |
| A10 | Bn-A10-p11358033 | 31.101 |
| A10 | Bn-A10-p11321778 | 31.783 |
| A10 | Bn-A10-p11376164 | 32.371 |
| A10 | Bn-A10-p13038966 | 36.515 |
| A10 | Bn-A10-p13164379 | 37.416 |
| A10 | Bn-A10-p13130002 | 37.703 |
| A10 | Bn-A10-p13169213 | 37.865 |
| A10 | Bn-A10-p13243690 | 38.674 |
| A10 | Bn-A10-p13606837 | 39.153 |
| A10 | Bn-A10-p13387522 | 39.412 |
| A10 | Bn-A10-p13410287 | 39.69 |
| A10 | Bn-A10-p13396422 | 39.747 |
| A10 | Bn-A10-p13393956 | 40.07 |
| A10 | Bn-A10-p13318061 | 40.226 |
| A10 | Bn-A10-p13788629 | 41.14 |
| A10 | Bn-A10-p13817274 | 41.501 |
| A10 | Bn-A10-p13330145 | 42.441 |
| A10 | Bn-A10-p13882091 | 43.437 |
| C01 | Bn-scaff_15838_1-p747634 | 0 |
| C01 | Bn-scaff_15838_1-p793596 | 0.713 |
| C01 | Bn-scaff_15838_1-p1218165 | 4.089 |
| C01 | Bn-scaff_15838_1-p1295927 | 4.424 |
| C01 | Bn-scaff_15838_1-p1199011 | 4.506 |
| C01 | Bn-scaff_15838_1-p1293912 | 4.586 |
| C01 | Bn-scaff_15838_1-p1403775 | 6.298 |
| C01 | Bn-scaff_15838_1-p1466722 | 6.721 |
| C01 | Bn-scaff_15838_1-p1551711 | 6.753 |
| C01 | Bn-scaff_15838_1-p1917196 | 7.951 |
| C01 | Bn-scaff_15838_1-p1881763 | 8.703 |
| C01 | Bn-scaff_15838_1-p2039607 | 8.968 |
| C01 | Bn-scaff_15838_1-p2253503 | 13.118 |
| C01 | Bn-scaff_15838_5-p171444 | 15.615 |
| C01 | Bn-scaff_15838_5-p171926 | 15.621 |
| C01 | Bn-scaff_15838_5-p160091 | 15.707 |
| C01 | Bn-scaff_15838_5-p151738 | 15.733 |
| C01 | Bn-scaff_15838_5-p146329 | 15.735 |
| C01 | Bn-scaff_15838_5-p165094 | 15.968 |
| C01 | Bn-scaff_15838_5-p202432 | 16.767 |
| C01 | Bn-scaff_19193_1-p399060 | 32.257 |
| C01 | Bn-scaff_19193_1-p229038 | 33.171 |
| C01 | Bn-scaff_19193_1-p470629 | 33.516 |
| C01 | Bn-scaff_19193_1-p408105 | 33.887 |
| C01 | Bn-scaff_27215_1-p909 | 34.313 |
| C01 | Bn-scaff_19193_1-p198467 | 34.963 |
| C01 | Bn-scaff_17731_1-p231809 | 36.316 |
| C01 | Bn-scaff_17731_1-p166950 | 36.502 |
| C01 | Bn-scaff_17731_1-p285840 | 36.508 |
| C01 | Bn-scaff_17731_1-p240944 | 37.412 |
| C01 | Bn-scaff_17731_1-p256496 | 37.424 |
| C01 | Bn-scaff_17731_1-p328403 | 37.64 |
| C01 | Bn-scaff_17731_1-p253178 | 37.947 |
| C01 | Bn-scaff_17827_1-p111920 | 41.593 |
| C01 | Bn-scaff_17731_1-p721469 | 42.054 |
| C01 | Bn-scaff_17731_1-p757660 | 42.319 |
| C01 | Bn-scaff_17731_1-p812656 | 42.351 |
| C01 | Bn-scaff_17827_1-p772345 | 44.393 |
| C01 | Bn-scaff_20210_1-p448678 | 46.422 |
| C01 | Bn-scaff_20210_1-p310895 | 48.712 |
| C01 | Bn-scaff_20210_1-p267408 | 49.35 |
| C01 | Bn-scaff_20210_1-p171615 | 49.732 |
| C01 | Bn-scaff_20210_1-p161976 | 50.092 |
| C01 | Bn-scaff_16128_2-p33988 | 52.521 |
| C01 | Bn-scaff_16128_2-p124279 | 53.379 |
| C01 | Bn-scaff_16128_2-p123105 | 53.386 |
| C01 | Bn-scaff_17592_1-p543445 | 57.148 |
| C01 | Bn-scaff_17592_1-p775877 | 57.539 |
| C01 | Bn-scaff_17592_1-p640754 | 58.277 |
| C01 | Bn-scaff_17592_1-p642148 | 58.298 |
| C01 | Bn-scaff_17592_1-p640689 | 58.317 |
| C01 | Bn-scaff_17592_1-p799061 | 58.633 |
| C01 | Bn-scaff_17369_1-p1207575 | 60.345 |
| C01 | Bn-scaff_17369_1-p1130686 | 60.838 |
| C01 | Bn-scaff_17369_1-p1088627 | 61.184 |
| C01 | Bn-scaff_17369_1-p1052933 | 61.635 |
| C01 | Bn-scaff_17369_1-p1060758 | 62.101 |
| C01 | Bn-scaff_17369_1-p941296 | 63.047 |
| C01 | Bn-scaff_17369_1-p829445 | 63.585 |
| C01 | Bn-scaff_17369_1-p804282 | 63.643 |
| C01 | Bn-scaff_17369_1-p761323 | 63.851 |
| C01 | Bn-scaff_17369_1-p837839 | 64.809 |
| C01 | Bn-scaff_17369_1-p847643 | 64.994 |
| C01 | Bn-scaff_17369_1-p271166 | 65.692 |
| C01 | Bn-scaff_17369_1-p740558 | 66.409 |
| C01 | Bn-scaff_17369_1-p627357 | 66.428 |
| C01 | Bn-scaff_17369_1-p386575 | 66.941 |
| C01 | Bn-scaff_24947_1-p89738 | 70.946 |
| C01 | Bn-scaff_24947_1-p112023 | 71.066 |
| C01 | Bn-scaff_18636_1-p51263 | 72.235 |
| C01 | Bn-scaff_19614_1-p293274 | 72.946 |
| C01 | Bn-scaff_19614_1-p325704 | 73.065 |
| C01 | Bn-scaff_20250_1-p310422 | 87.382 |
| C01 | Bn-scaff_20250_1-p387005 | 87.849 |
| C01 | Bn-scaff_16691_1-p785747 | 89.734 |
| C01 | Bn-scaff_15879_1-p75940 | 93.27 |
| C01 | Bn-scaff_15879_1-p79732 | 93.272 |
| C01 | Bn-scaff_15879_1-p386825 | 93.281 |
| C01 | Bn-scaff_21225_1-p110619 | 93.618 |
| C01 | Bn-scaff_17517_1-p284998 | 94.367 |
| C01 | Bn-scaff_17517_1-p298351 | 94.421 |
| C01 | Bn-scaff_17517_1-p322771 | 94.434 |
| C01 | Bn-scaff_16055_1-p1269543 | 95.397 |
| C01 | Bn-scaff_16055_1-p1222440 | 95.414 |
| C01 | Bn-scaff_17517_1-p355834 | 95.793 |
| C01 | Bn-scaff_15713_1-p2658 | 95.8 |
| C01 | Bn-scaff_17517_1-p496055 | 95.811 |
| C01 | Bn-scaff_17517_1-p536489 | 95.856 |
| C01 | Bn-scaff_17517_1-p355771 | 95.857 |
| C01 | Bn-scaff_17517_1-p536271 | 95.877 |
| C01 | Bn-scaff_17517_1-p681084 | 95.923 |
| C01 | Bn-scaff_17517_1-p683786 | 95.938 |
| C01 | Bn-scaff_17517_1-p681915 | 95.946 |
| C01 | Bn-scaff_17517_1-p681864 | 95.951 |
| C01 | Bn-scaff_17517_1-p360914 | 95.999 |
| C01 | Bn-scaff_17517_1-p600876 | 96.011 |
| C01 | Bn-scaff_21163_1-p3558 | 96.029 |
| C01 | Bn-scaff_17517_1-p536322 | 96.039 |
| C01 | Bn-scaff_16055_1-p1218688 | 96.26 |
| C01 | Bn-scaff_23795_1-p471265 | 96.262 |
| C01 | Bn-scaff_16055_1-p1268643 | 96.273 |
| C01 | Bn-scaff_17517_1-p332460 | 96.881 |
| C01 | Bn-scaff_17517_1-p330218 | 96.947 |
| C01 | Bn-scaff_17517_1-p286607 | 97.007 |
| C01 | Bn-scaff_21225_1-p74961 | 97.828 |
| C01 | Bn-scaff_15879_1-p29379 | 98.257 |
| C01 | Bn-scaff_15879_1-p690531 | 98.725 |
| C01 | Bn-scaff_17515_1-p396838 | 108.673 |
| C01 | Bn-scaff_17515_1-p390772 | 108.813 |
| C01 | Bn-scaff_17515_1-p390603 | 108.969 |
| C01 | Bn-scaff_17515_1-p388362 | 109.145 |
| C01 | Bn-scaff_18672_1-p168747 | 110.826 |
| C01 | Bn-scaff_18672_1-p169758 | 110.845 |
| C01 | Bn-scaff_18672_1-p140782 | 111.279 |
| C01 | Bn-scaff_23799_1-p51418 | 111.897 |
| C01 | Bn-scaff_23347_1-p83009 | 111.922 |
| C01 | Bn-scaff_15712_3-p804205 | 125.837 |
| C01 | Bn-scaff_15712_3-p818174 | 125.847 |
| C02 | Bn-scaff_22970_1-p491664 | 0 |
| C02 | Bn-scaff_22970_1-p372961 | 1.194 |
| C02 | Bn-scaff_15714_1-p3087640 | 2.91 |
| C02 | Bn-scaff_15714_1-p3065920 | 3.004 |
| C02 | Bn-scaff_15714_1-p3001398 | 3.666 |
| C02 | Bn-scaff_15714_1-p2113009 | 10.39 |
| C02 | Bn-scaff_15714_1-p2051174 | 11.654 |
| C02 | Bn-scaff_15714_1-p1900599 | 14.403 |
| C02 | Bn-scaff_15714_1-p1014099 | 23.008 |
| C02 | Bn-scaff_15714_1-p966029 | 23.564 |
| C02 | Bn-scaff_15714_1-p833531 | 24.062 |
| C02 | Bn-scaff_15714_1-p835459 | 24.094 |
| C02 | Bn-scaff_15714_1-p832902 | 24.097 |
| C02 | Bn-scaff_15714_1-p801408 | 25.219 |
| C02 | Bn-scaff_15714_1-p790349 | 25.234 |
| C02 | Bn-scaff_15714_1-p780553 | 25.274 |
| C02 | Bn-scaff_15714_1-p692582 | 25.286 |
| C02 | Bn-scaff_15714_1-p773455 | 25.426 |
| C02 | Bn-scaff_15714_1-p774503 | 25.645 |
| C02 | Bn-scaff_15714_1-p633772 | 25.655 |
| C02 | Bn-scaff_16825_1-p56159 | 28.637 |
| C02 | Bn-scaff_22527_1-p156123 | 31.669 |
| C02 | Bn-scaff_17522_1-p776775 | 37.134 |
| C02 | Bn-scaff_17522_1-p681005 | 37.993 |
| C02 | Bn-scaff_17522_1-p651309 | 38.313 |
| C02 | Bn-scaff_17522_1-p462969 | 38.325 |
| C02 | Bn-scaff_17522_1-p479182 | 38.336 |
| C02 | Bn-scaff_17522_1-p505332 | 38.339 |
| C02 | Bn-scaff_17522_1-p397676 | 38.9 |
| C02 | Bn-scaff_17522_1-p130963 | 41.302 |
| C02 | Bn-scaff_16139_1-p720716 | 48.826 |
| C02 | Bn-scaff_16139_1-p721705 | 49.103 |
| C02 | Bn-scaff_16139_1-p653514 | 49.331 |
| C02 | Bn-scaff_16139_1-p643296 | 49.374 |
| C02 | Bn-scaff_16139_1-p643335 | 49.418 |
| C02 | Bn-scaff_16139_1-p434160 | 51.009 |
| C02 | Bn-scaff_16139_1-p259289 | 51.574 |
| C02 | Bn-scaff_16139_1-p198539 | 52.14 |
| C02 | Bn-scaff_16139_1-p127959 | 53.259 |
| C02 | Bn-scaff_22093_1-p102888 | 67.862 |
| C02 | Bn-scaff_22093_1-p33169 | 67.94 |
| C02 | Bn-scaff_18675_1-p812043 | 71.175 |
| C02 | Bn-scaff_18675_1-p919422 | 71.574 |
| C02 | Bn-scaff_17289_1-p458549 | 78.179 |
| C02 | Bn-scaff_17289_1-p655703 | 78.778 |
| C02 | Bn-scaff_18360_1-p164180 | 80.517 |
| C02 | Bn-scaff_18360_1-p358559 | 83.836 |
| C02 | Bn-scaff_15712_2-p771303 | 93.432 |
| C02 | Bn-scaff_15712_9-p411591 | 93.981 |
| C02 | Bn-scaff_15712_2-p800968 | 93.986 |
| C03 | Bn-scaff_16614_1-p1161603 | 0 |
| C03 | Bn-scaff_16614_1-p226437 | 4.939 |
| C03 | Bn-scaff_27198_1-p254278 | 5.275 |
| C03 | Bn-scaff_18936_1-p297756 | 11.512 |
| C03 | Bn-scaff_18936_1-p297918 | 11.567 |
| C03 | Bn-scaff_18936_1-p472353 | 12.638 |
| C03 | Bn-scaff_18936_1-p384730 | 12.765 |
| C03 | Bn-scaff_18936_1-p855050 | 15.612 |
| C03 | Bn-scaff_15877_1-p468499 | 16.977 |
| C03 | Bn-scaff_15877_1-p416968 | 17.315 |
| C03 | Bn-scaff_15877_1-p178765 | 17.572 |
| C03 | Bn-scaff_15877_1-p420181 | 17.588 |
| C03 | Bn-scaff_15877_1-p454328 | 17.826 |
| C03 | Bn-scaff_15877_1-p147053 | 17.974 |
| C03 | Bn-scaff_15877_1-p423112 | 18.079 |
| C03 | Bn-scaff_15877_1-p19866 | 18.481 |
| C03 | Bn-scaff_15877_1-p476034 | 19.5 |
| C03 | Bn-scaff_21778_1-p356034 | 29.471 |
| C03 | Bn-scaff_22728_1-p1343876 | 30.609 |
| C03 | Bn-scaff_21778_1-p388076 | 31.096 |
| C03 | Bn-scaff_22728_1-p1309552 | 32.595 |
| C03 | Bn-scaff_22728_1-p1310107 | 32.826 |
| C03 | Bn-scaff_22728_1-p1256248 | 33.473 |
| C03 | Bn-scaff_22728_1-p1197524 | 34.715 |
| C03 | Bn-scaff_22728_1-p1197316 | 34.739 |
| C03 | Bn-scaff_22728_1-p1181721 | 34.776 |
| C03 | Bn-scaff_22728_1-p1018084 | 36.572 |
| C03 | Bn-scaff_22728_1-p967531 | 36.575 |
| C03 | Bn-scaff_22728_1-p600933 | 40.845 |
| C03 | Bn-scaff_22728_1-p646719 | 41.343 |
| C03 | Bn-scaff_22728_1-p577795 | 41.801 |
| C03 | Bn-scaff_22728_1-p400151 | 44.229 |
| C03 | Bn-scaff_22728_1-p359656 | 45.106 |
| C03 | Bn-scaff_22728_1-p313976 | 45.869 |
| C03 | Bn-scaff_22728_1-p283916 | 46.155 |
| C03 | Bn-scaff_22728_1-p305373 | 46.542 |
| C03 | Bn-scaff_22728_1-p304845 | 46.546 |
| C03 | Bn-scaff_22728_1-p263173 | 46.975 |
| C03 | Bn-scaff_18322_1-p2583107 | 49.994 |
| C03 | Bn-A03-p5800566 | 50.293 |
| C03 | Bn-scaff_18322_1-p2266003 | 50.317 |
| C03 | Bn-scaff_18322_1-p2514719 | 50.353 |
| C03 | Bn-scaff_18322_1-p2560823 | 50.494 |
| C03 | Bn-scaff_18322_1-p2509158 | 50.522 |
| C03 | Bn-scaff_18322_1-p2518158 | 50.564 |
| C03 | Bn-scaff_18322_1-p2121265 | 52.108 |
| C03 | Bn-scaff_18322_1-p2084715 | 52.315 |
| C03 | Bn-scaff_18322_1-p1751672 | 52.921 |
| C03 | Bn-scaff_18322_1-p1854764 | 53.789 |
| C03 | Bn-scaff_18322_1-p1622028 | 55.578 |
| C03 | Bn-scaff_18322_1-p1566336 | 56.454 |
| C03 | Bn-scaff_18322_1-p1540260 | 56.793 |
| C03 | Bn-scaff_18322_1-p1490180 | 57.385 |
| C03 | Bn-scaff_18322_1-p1490938 | 57.386 |
| C03 | Bn-scaff_18322_1-p1473638 | 57.978 |
| C03 | Bn-scaff_18322_1-p1468468 | 57.979 |
| C03 | Bn-scaff_18322_1-p1486915 | 57.983 |
| C03 | Bn-scaff_18322_1-p1361529 | 58.365 |
| C03 | Bn-scaff_18322_1-p908592 | 60.987 |
| C03 | Bn-scaff_18322_1-p881842 | 61.339 |
| C03 | Bn-scaff_18322_1-p805531 | 61.539 |
| C03 | Bn-scaff_18322_1-p821577 | 61.658 |
| C03 | Bn-scaff_18322_1-p811742 | 61.844 |
| C03 | Bn-scaff_18322_1-p758030 | 62.998 |
| C03 | Bn-scaff_18322_1-p649152 | 65.9 |
| C03 | Bn-scaff_18322_1-p361468 | 68.075 |
| C03 | Bn-scaff_21312_1-p1332032 | 72.638 |
| C03 | Bn-scaff_21312_1-p1309658 | 72.657 |
| C03 | Bn-scaff_21312_1-p1305411 | 73.787 |
| C03 | Bn-scaff_21312_1-p681690 | 76.13 |
| C03 | Bn-scaff_21312_1-p546514 | 76.725 |
| C03 | Bn-scaff_23954_1-p1127781 | 76.962 |
| C03 | Bn-scaff_19111_1-p177679 | 77.019 |
| C03 | Bn-scaff_19111_1-p161862 | 77.033 |
| C03 | Bn-scaff_19111_1-p163155 | 77.049 |
| C03 | Bn-scaff_19111_1-p198604 | 77.073 |
| C03 | Bn-scaff_21312_1-p102482 | 77.203 |
| C03 | Bn-scaff_23954_1-p1076994 | 77.297 |
| C03 | Bn-scaff_23954_1-p661198 | 79.629 |
| C03 | Bn-scaff_16002_1-p2292068 | 83.223 |
| C03 | Bn-scaff_16002_1-p2321291 | 83.234 |
| C03 | Bn-scaff_16002_1-p2299056 | 83.501 |
| C03 | Bn-scaff_16002_1-p2217361 | 84.074 |
| C03 | Bn-scaff_16002_1-p2135081 | 84.474 |
| C03 | Bn-scaff_16002_1-p1717929 | 87.331 |
| C03 | Bn-scaff_16002_1-p1762565 | 87.334 |
| C03 | Bn-scaff_16002_1-p1688212 | 87.388 |
| C03 | Bn-scaff_16002_1-p1627403 | 87.404 |
| C03 | Bn-scaff_18936_1-p1265461 | 91.525 |
| C03 | Bn-scaff_16002_1-p511265 | 91.975 |
| C03 | Bn-scaff_16002_1-p420333 | 92.334 |
| C03 | Bn-scaff_16002_1-p397940 | 92.534 |
| C03 | Bn-scaff_16002_1-p298783 | 92.817 |
| C03 | Bn-scaff_16002_1-p216389 | 93.507 |
| C03 | Bn-scaff_16352_1-p287559 | 101.755 |
| C03 | Bn-scaff_16352_1-p338900 | 102.13 |
| C03 | Bn-scaff_16352_1-p361447 | 102.697 |
| C03 | Bn-scaff_20646_1-p677296 | 109.765 |
| C03 | Bn-scaff_20646_1-p678479 | 109.786 |
| C03 | Bn-scaff_20646_1-p586593 | 110.714 |
| C03 | Bn-scaff_20646_1-p252893 | 111.512 |
| C03 | Bn-scaff_20646_1-p226621 | 111.56 |
| C03 | Bn-scaff_20646_1-p246974 | 111.563 |
| C03 | Bn-scaff_20646_1-p290576 | 112.083 |
| C03 | Bn-scaff_20646_1-p472495 | 112.544 |
| C03 | Bn-scaff_20646_1-p463899 | 112.597 |
| C03 | Bn-scaff_20646_1-p293576 | 112.613 |
| C03 | Bn-scaff_20646_1-p564345 | 112.847 |
| C03 | Bn-A03-p12737336 | 112.862 |
| C03 | Bn-scaff_20646_1-p644913 | 113.332 |
| C03 | Bn-scaff_19523_1-p188656 | 115.813 |
| C03 | Bn-scaff_19523_1-p149 | 115.822 |
| C03 | Bn-scaff_20741_1-p63208 | 115.827 |
| C03 | Bn-scaff_27313_1-p451 | 117.049 |
| C03 | Bn-scaff_28509_1-p33640 | 117.05 |
| C03 | Bn-scaff_27313_1-p653 | 117.127 |
| C03 | Bn-scaff_18482_1-p739133 | 121.052 |
| C03 | Bn-scaff_26320_1-p220425 | 143.636 |
| C03 | Bn-scaff_18855_1-p185593 | 146.969 |
| C03 | Bn-scaff_18855_1-p184900 | 146.996 |
| C03 | Bn-scaff_25094_1-p413818 | 148.158 |
| C03 | Bn-scaff_25094_1-p126581 | 149.957 |
| C03 | Bn-scaff_25094_1-p131956 | 149.994 |
| C03 | Bn-scaff_19109_1-p33401 | 150.054 |
| C03 | Bn-scaff_25094_1-p154241 | 150.393 |
| C03 | Bn-scaff_25094_1-p316855 | 150.604 |
| C03 | Bn-scaff_23407_1-p36212 | 166.388 |
| C03 | Bn-scaff_23407_1-p27434 | 166.986 |
| C03 | Bn-scaff_19740_1-p36568 | 167.437 |
| C03 | Bn-scaff_23407_1-p23075 | 167.454 |
| C03 | Bn-scaff_16135_1-p168458 | 167.998 |
| C03 | Bn-scaff_16135_1-p290412 | 169.691 |
| C03 | Bn-scaff_16135_1-p311707 | 170.927 |
| C03 | Bn-scaff_27747_1-p27050 | 174.425 |
| C03 | Bn-scaff_18849_1-p30714 | 174.999 |
| C03 | Bn-scaff_17440_1-p938541 | 175.577 |
| C03 | Bn-scaff_17440_1-p364477 | 176.996 |
| C03 | Bn-scaff_17440_1-p107030 | 179.142 |
| C03 | Bn-scaff_16372_1-p89003 | 179.274 |
| C03 | Bn-scaff_17440_1-p68056 | 179.39 |
| C03 | Bn-scaff_24726_1-p39937 | 179.405 |
| C03 | Bn-scaff_24726_1-p90002 | 179.53 |
| C03 | Bn-scaff_24726_1-p16408 | 181.014 |
| C03 | Bn-scaff_16665_1-p188604 | 181.587 |
| C03 | Bn-scaff_24631_1-p299275 | 184.781 |
| C03 | Bn-scaff_24631_1-p348568 | 184.783 |
| C03 | Bn-scaff_24631_1-p316671 | 184.825 |
| C03 | Bn-scaff_24631_1-p373952 | 185.417 |
| C03 | Bn-scaff_24631_1-p424973 | 186.783 |
| C03 | Bn-scaff_17042_1-p507777 | 202.657 |
| C03 | Bn-scaff_17042_1-p500227 | 202.673 |
| C03 | Bn-scaff_17042_1-p478613 | 202.838 |
| C03 | Bn-scaff_17042_1-p511184 | 204.023 |
| C03 | Bn-scaff_17457_1-p147858 | 209.813 |
| C03 | Bn-scaff_19101_1-p13013 | 209.82 |
| C03 | Bn-scaff_17457_1-p245329 | 209.869 |
| C03 | Bn-scaff_18559_1-p166394 | 226.109 |
| C03 | Bn-scaff_23761_1-p27239 | 226.765 |
| C03 | Bn-scaff_23761_1-p738582 | 227.448 |
| C03 | Bn-scaff_23761_1-p28016 | 227.589 |
| C03 | Bn-scaff_23761_1-p281604 | 227.739 |
| C03 | Bn-scaff_17119_1-p417871 | 228.217 |
| C03 | Bn-scaff_23761_1-p386442 | 229.546 |
| C03 | Bn-scaff_23761_1-p392078 | 229.621 |
| C03 | Bn-scaff_23761_1-p738585 | 230.143 |
| C03 | Bn-scaff_23761_1-p581335 | 230.146 |
| C04 | Bn-scaff_22933_1-p76584 | 0 |
| C04 | Bn-scaff_15838_1-p92309 | 3.913 |
| C04 | Bn-scaff_15838_1-p96143 | 3.974 |
| C04 | Bn-scaff_15832_1-p52737 | 5.402 |
| C04 | Bn-scaff_23534_1-p36688 | 6.405 |
| C04 | Bn-scaff_16027_1-p70704 | 11.098 |
| C04 | Bn-scaff_28277_1-p5229 | 23.207 |
| C04 | Bn-scaff_16534_1-p524861 | 26.527 |
| C04 | Bn-scaff_16534_1-p504933 | 27.057 |
| C04 | Bn-scaff_16534_1-p729809 | 28.694 |
| C04 | Bn-scaff_16534_1-p692624 | 29.066 |
| C04 | Bn-scaff_16534_1-p679107 | 29.077 |
| C04 | Bn-scaff_16534_1-p801233 | 29.953 |
| C04 | Bn-scaff_16534_1-p1112687 | 32.171 |
| C04 | Bn-scaff_16534_1-p1263272 | 34.008 |
| C04 | Bn-scaff_16534_1-p1421906 | 34.289 |
| C04 | Bn-scaff_16534_1-p1429525 | 34.542 |
| C04 | Bn-scaff_16534_1-p1267478 | 35.366 |
| C04 | Bn-scaff_16935_1-p69744 | 39.597 |
| C04 | Bn-scaff_16534_1-p1862345 | 40.055 |
| C04 | Bn-scaff_17869_1-p813891 | 58.083 |
| C04 | Bn-scaff_17869_1-p815096 | 58.933 |
| C04 | Bn-scaff_17869_1-p820117 | 59.419 |
| C04 | Bn-A05-p6535233 | 59.8 |
| C04 | Bn-scaff_16095_1-p24601 | 61.332 |
| C04 | Bn-scaff_18754_1-p207838 | 62.505 |
| C04 | Bn-scaff_16576_1-p166604 | 63.612 |
| C04 | Bn-scaff_16576_1-p579161 | 64.414 |
| C04 | Bn-scaff_17112_1-p3928 | 64.788 |
| C04 | Bn-scaff_18712_1-p326442 | 68.68 |
| C04 | Bn-scaff_18712_1-p482601 | 69.114 |
| C04 | Bn-scaff_18712_1-p627372 | 69.368 |
| C04 | Bn-scaff_27513_1-p55735 | 69.457 |
| C04 | Bn-scaff_18656_1-p22362 | 69.927 |
| C04 | Bn-scaff_16309_1-p215682 | 70.242 |
| C04 | Bn-scaff_16312_1-p189576 | 70.514 |
| C04 | Bn-scaff_16217_1-p398497 | 70.747 |
| C04 | Bn-scaff_16217_1-p395934 | 70.802 |
| C04 | Bn-scaff_18505_1-p216449 | 70.999 |
| C04 | Bn-scaff_18505_1-p288042 | 71.018 |
| C04 | Bn-scaff_15911_1-p358353 | 71.298 |
| C04 | Bn-scaff_19170_1-p891168 | 71.303 |
| C04 | Bn-scaff_15911_1-p316804 | 71.354 |
| C04 | Bn-scaff_15911_1-p336033 | 71.368 |
| C04 | Bn-scaff_15911_1-p753773 | 71.405 |
| C04 | Bn-scaff_22530_1-p56354 | 71.413 |
| C04 | Bn-scaff_15911_1-p253367 | 71.419 |
| C04 | Bn-scaff_19170_1-p1087763 | 71.539 |
| C04 | Bn-scaff_20042_1-p1582 | 71.69 |
| C04 | Bn-scaff_16312_1-p33849 | 71.935 |
| C04 | Bn-scaff_16312_1-p117605 | 72.033 |
| C04 | Bn-scaff_19575_1-p613320 | 72.315 |
| C04 | Bn-scaff_19170_1-p399579 | 72.39 |
| C04 | Bn-scaff_19170_1-p990940 | 72.425 |
| C04 | Bn-scaff_19170_1-p441956 | 72.442 |
| C04 | Bn-scaff_16511_1-p1231824 | 72.574 |
| C04 | Bn-scaff_16511_1-p854981 | 72.575 |
| C04 | Bn-scaff_19253_1-p614151 | 72.584 |
| C04 | Bn-scaff_16511_1-p1127864 | 72.615 |
| C04 | Bn-scaff_16511_1-p986462 | 72.626 |
| C04 | Bn-scaff_16511_1-p744766 | 72.638 |
| C04 | Bn-scaffold18039-p397 | 72.643 |
| C04 | Bn-scaff_16511_1-p745248 | 72.652 |
| C04 | Bn-scaff_19253_1-p543342 | 72.653 |
| C04 | Bn-scaff_19253_1-p490594 | 72.654 |
| C04 | Bn-scaff_27634_1-p13119 | 72.658 |
| C04 | Bn-scaff_15809_1-p88785 | 72.68 |
| C04 | Bn-scaff_28498_1-p299166 | 72.681 |
| C04 | Bn-scaff_16511_1-p718956 | 72.691 |
| C04 | Bn-scaff_16503_1-p197277 | 72.707 |
| C04 | Bn-scaff_16511_1-p854762 | 72.709 |
| C04 | Bn-scaff_19170_1-p40115 | 72.818 |
| C04 | Bn-scaff_19170_1-p518890 | 72.901 |
| C04 | Bn-scaff_16312_1-p120198 | 73.226 |
| C04 | Bn-scaff_16312_1-p150941 | 73.422 |
| C04 | Bn-scaff_16217_1-p112302 | 74.187 |
| C04 | Bn-scaff_16217_1-p114749 | 74.274 |
| C04 | Bn-scaff_16714_1-p220536 | 74.355 |
| C04 | Bn-scaff_25432_1-p16404 | 74.359 |
| C04 | Bn-scaff_16714_1-p196141 | 74.721 |
| C04 | Bn-scaff_16714_1-p171706 | 74.722 |
| C04 | Bn-scaff_16714_1-p406026 | 74.729 |
| C04 | Bn-scaff_16714_1-p104848 | 74.795 |
| C04 | Bn-scaff_16714_1-p225057 | 74.904 |
| C04 | Bn-scaff_18656_1-p36077 | 75.591 |
| C04 | Bn-scaff_16309_1-p137984 | 76.026 |
| C04 | Bn-scaff_17335_1-p119237 | 77.241 |
| C04 | Bn-scaff_15712_8-p110489 | 82.733 |
| C04 | Bn-scaff_18776_1-p246454 | 85.929 |
| C04 | Bn-scaff_18776_1-p269180 | 86.364 |
| C04 | Bn-scaff_15779_1-p318634 | 88.779 |
| C04 | Bn-scaff_26139_1-p209686 | 88.943 |
| C04 | Bn-scaff_20191_1-p63120 | 89.259 |
| C04 | Bn-scaff_15779_1-p400004 | 89.339 |
| C04 | Bn-scaff_27765_1-p403263 | 89.343 |
| C04 | Bn-scaff_26139_1-p270329 | 89.445 |
| C04 | Bn-scaff_26139_1-p294830 | 89.449 |
| C04 | Bn-scaff_26139_1-p312653 | 89.46 |
| C04 | Bn-scaff_18062_1-p219780 | 90.995 |
| C04 | Bn-scaff_18062_1-p148513 | 91.009 |
| C04 | Bn-scaff_18062_1-p150109 | 91.037 |
| C04 | Bn-scaff_18062_1-p219340 | 91.076 |
| C04 | Bn-scaff_25740_1-p10342 | 91.122 |
| C04 | Bn-scaff_18062_1-p45782 | 91.423 |
| C04 | Bn-scaff_16876_1-p1178318 | 94.814 |
| C04 | Bn-scaff_16876_1-p1116580 | 95.118 |
| C04 | Bn-scaff_16876_1-p1293441 | 95.202 |
| C04 | Bn-scaff_16876_1-p1080820 | 95.75 |
| C04 | Bn-scaff_16876_1-p603840 | 98.117 |
| C04 | Bn-scaff_16876_1-p171510 | 98.632 |
| C04 | Bn-scaff_16876_1-p176872 | 98.663 |
| C04 | Bn-scaff_15712_6-p764905 | 102.176 |
| C04 | Bn-scaff_19043_1-p440830 | 105.026 |
| C04 | Bn-scaff_19043_1-p53629 | 105.476 |
| C04 | Bn-scaff_19043_1-p18178 | 105.605 |
| C04 | Bn-scaff_19043_1-p51461 | 105.725 |
| C04 | Bn-scaff_19043_1-p18086 | 105.749 |
| C04 | Bn-scaff_23472_1-p52830 | 106.187 |
| C04 | Bn-scaff_15798_1-p29358 | 106.239 |
| C04 | Bn-scaff_23472_1-p21637 | 106.25 |
| C04 | Bn-scaff_15798_1-p13261 | 106.26 |
| C04 | Bn-scaff_23472_1-p56099 | 106.29 |
| C04 | Bn-scaff_15798_1-p15346 | 106.427 |
| C04 | Bn-scaff_15798_1-p227450 | 106.786 |
| C04 | Bn-scaff_15798_1-p131623 | 106.812 |
| C04 | Bn-scaff_15798_1-p106090 | 106.857 |
| C04 | Bn-scaff_15798_1-p81345 | 106.97 |
| C04 | Bn-scaff_16888_1-p1416280 | 128.474 |
| C04 | Bn-scaff_16888_1-p1450603 | 128.479 |
| C04 | Bn-scaff_16888_1-p1419203 | 128.482 |
| C04 | Bn-scaff_16888_1-p1419612 | 128.501 |
| C04 | Bn-scaff_16888_1-p1382480 | 129.164 |
| C04 | Bn-scaff_16888_1-p1538459 | 129.549 |
| C04 | Bn-scaff_16888_1-p1554563 | 129.609 |
| C04 | Bn-scaff_26787_1-p30400 | 136.184 |
| C04 | Bn-scaff_20270_1-p1013122 | 140.288 |
| C04 | Bn-scaff_18903_1-p47239 | 141.638 |
| C04 | Bn-scaff_18903_1-p38399 | 141.726 |
| C04 | Bn-scaff_18903_1-p735820 | 144.105 |
| C04 | Bn-scaff_18903_1-p137109 | 145.01 |
| C04 | Bn-scaff_18903_1-p893924 | 146.02 |
| C04 | Bn-scaff_27676_1-p110403 | 147.773 |
| C04 | Bn-scaff_27676_1-p111217 | 147.774 |
| C04 | Bn-scaff_20817_1-p15523 | 148.852 |
| C04 | Bn-scaff_20817_1-p50242 | 149.135 |
| C04 | Bn-scaff_20817_1-p60579 | 149.73 |
| C04 | Bn-scaff_20817_1-p56685 | 149.741 |
| C05 | Bn-scaff_20901_1-p1681305 | 0 |
| C05 | Bn-scaff_20901_1-p1510260 | 2.857 |
| C05 | Bn-scaff_20901_1-p1311264 | 4.905 |
| C05 | Bn-scaff_28179_1-p15387 | 8.814 |
| C05 | Bn-scaff_20901_1-p1110262 | 9.427 |
| C05 | Bn-scaff_20901_1-p901752 | 12.618 |
| C05 | Bn-scaff_20901_1-p948057 | 13.02 |
| C05 | Bn-scaff_20901_1-p890950 | 13.025 |
| C05 | Bn-scaff_20901_1-p879369 | 14.225 |
| C05 | Bn-scaff_20901_1-p854673 | 15.34 |
| C05 | Bn-scaff_15838_2-p97896 | 24.158 |
| C05 | Bn-scaff_15838_2-p126220 | 24.165 |
| C05 | Bn-scaff_19641_1-p188036 | 25.875 |
| C05 | Bn-scaff_15712_10-p380937 | 29.916 |
| C05 | Bn-scaff_21496_1-p440186 | 31.666 |
| C05 | Bn-scaff_21496_1-p191960 | 31.903 |
| C05 | Bn-scaff_21496_1-p433453 | 32.558 |
| C05 | Bn-scaff_18181_1-p1745427 | 37.653 |
| C05 | Bn-scaff_18181_1-p1707481 | 38.098 |
| C05 | Bn-scaff_18181_1-p962029 | 45.339 |
| C05 | Bn-scaff_18181_1-p954167 | 45.626 |
| C05 | Bn-scaff_18181_1-p176182 | 47.936 |
| C05 | Bn-scaff_16093_1-p5778 | 59.444 |
| C05 | Bn-scaff_20125_1-p294686 | 63.035 |
| C05 | Bn-scaff_20125_1-p378742 | 63.048 |
| C05 | Bn-scaff_21821_1-p112456 | 63.505 |
| C05 | Bn-scaff_21338_1-p832648 | 65.84 |
| C05 | Bn-scaff_16268_1-p573752 | 68.496 |
| C05 | Bn-scaff_16268_1-p608221 | 68.526 |
| C05 | Bn-scaff_16268_1-p641008 | 68.552 |
| C05 | Bn-scaff_21338_1-p1197474 | 68.862 |
| C05 | Bn-scaff_21338_1-p1197350 | 68.882 |
| C05 | Bn-scaff_21338_1-p1199395 | 68.89 |
| C05 | Bn-scaff_21338_1-p1193513 | 69.003 |
| C05 | Bn-scaff_17869_1-p843753 | 72.442 |
| C05 | Bn-scaff_18338_1-p322932 | 73.175 |
| C05 | Bn-scaff_15818_2-p1027337 | 75.106 |
| C05 | Bn-scaff_25595_1-p423448 | 79.376 |
| C05 | Bn-scaff_20219_1-p193578 | 105.006 |
| C05 | Bn-scaff_20219_1-p350516 | 105.85 |
| C05 | Bn-scaff_23186_1-p340682 | 108.583 |
| C05 | Bn-scaff_20270_1-p1054999 | 113.312 |
| C05 | Bn-scaff_20270_1-p1066725 | 113.424 |
| C05 | Bn-scaff_20270_1-p1070285 | 114.085 |
| C05 | Bn-scaff_20270_1-p1097990 | 114.459 |
| C05 | Bn-scaff_20270_1-p1106536 | 114.481 |
| C05 | Bn-scaff_23186_1-p8774 | 114.924 |
| C05 | Bn-scaff_16526_1-p42180 | 115.198 |
| C05 | Bn-scaff_20270_1-p1247236 | 115.999 |
| C05 | Bn-scaff_20270_1-p1333525 | 117.236 |
| C05 | Bn-scaff_20270_1-p1440999 | 118.508 |
| C06 | Bn-scaff_17799_1-p2079459 | 0 |
| C06 | Bn-scaff_17799_1-p2065835 | 0.767 |
| C06 | Bn-scaff_17799_1-p2111711 | 0.779 |
| C06 | Bn-scaff_17799_1-p2280106 | 1.347 |
| C06 | Bn-scaff_17799_1-p2278846 | 1.605 |
| C06 | Bn-scaff_16547_1-p85743 | 19.827 |
| C06 | Bn-scaff_23821_1-p65006 | 20.06 |
| C06 | Bn-scaff_18807_1-p498993 | 20.082 |
| C06 | Bn-scaff_18807_1-p616022 | 20.152 |
| C06 | Bn-scaff_18807_1-p513632 | 20.178 |
| C06 | Bn-scaff_23957_1-p123234 | 20.183 |
| C06 | Bn-scaff_23957_1-p448548 | 20.197 |
| C06 | Bn-scaff_23821_1-p122397 | 20.201 |
| C06 | Bn-scaff_18807_1-p139558 | 20.205 |
| C06 | Bn-scaff_18807_1-p747031 | 20.208 |
| C06 | Bn-scaff_23957_1-p123191 | 20.212 |
| C06 | Bn-scaff_23957_1-p270579 | 20.235 |
| C06 | Bn-scaff_23957_1-p484997 | 20.269 |
| C06 | Bn-scaff_23957_1-p658269 | 20.544 |
| C06 | Bn-scaff_16547_1-p290890 | 20.785 |
| C06 | Bn-scaff_16547_1-p34318 | 20.814 |
| C06 | Bn-scaff_16547_1-p33100 | 20.877 |
| C06 | Bn-scaff_16547_1-p158886 | 20.898 |
| C06 | Bn-scaff_16874_1-p173544 | 23.263 |
| C06 | Bn-scaff_16874_1-p285169 | 23.311 |
| C06 | Bn-scaff_16874_1-p325632 | 23.387 |
| C06 | Bn-scaff_16865_1-p95084 | 29.449 |
| C06 | Bn-scaff_16064_1-p151843 | 29.868 |
| C06 | Bn-scaff_16064_1-p131677 | 29.877 |
| C06 | Bn-scaff_16064_1-p360023 | 31.076 |
| C06 | Bn-scaff_16064_1-p1073720 | 31.551 |
| C06 | Bn-scaff_16064_1-p1056112 | 32.334 |
| C06 | Bn-scaff_18439_1-p238960 | 35.625 |
| C06 | Bn-scaff_18439_1-p237947 | 35.995 |
| C06 | Bn-scaff_16064_1-p1418053 | 36.61 |
| C06 | Bn-scaff_18439_1-p208544 | 37.01 |
| C06 | Bn-scaff_18439_1-p262054 | 37.051 |
| C06 | Bn-scaff_18439_1-p219822 | 37.228 |
| C06 | Bn-scaff_25878_1-p8784 | 37.247 |
| C06 | Bn-scaff_15818_1-p3229379 | 38.076 |
| C06 | Bn-scaff_15818_1-p3172268 | 39.176 |
| C06 | Bn-scaff_15818_1-p3109988 | 39.789 |
| C06 | Bn-scaff_15818_1-p3034056 | 39.848 |
| C06 | Bn-scaff_15818_1-p3075274 | 39.879 |
| C06 | Bn-scaff_15818_1-p2891900 | 39.998 |
| C06 | Bn-scaff_15818_1-p2270500 | 43.229 |
| C06 | Bn-scaff_15818_1-p2029150 | 44.698 |
| C06 | Bn-scaff_15818_1-p2022143 | 44.884 |
| C06 | Bn-scaff_15763_1-p1401447 | 48.313 |
| C06 | Bn-scaff_15763_1-p1411892 | 48.478 |
| C06 | Bn-scaff_15763_1-p1492117 | 48.799 |
| C06 | Bn-scaff_15763_1-p1492349 | 48.897 |
| C06 | Bn-scaff_15763_1-p1501510 | 48.91 |
| C06 | Bn-scaff_15763_1-p1440307 | 49.277 |
| C06 | Bn-scaff_15763_1-p1460541 | 49.412 |
| C06 | Bn-A07-p12935190 | 52.829 |
| C06 | Bn-scaff_15763_1-p588323 | 53.26 |
| C06 | Bn-scaff_15763_1-p262418 | 53.819 |
| C06 | Bn-scaff_15763_1-p287565 | 53.836 |
| C06 | Bn-scaff_15763_1-p255875 | 53.841 |
| C06 | Bn-scaff_15763_1-p342497 | 53.846 |
| C06 | Bn-scaff_15763_1-p313075 | 54.366 |
| C06 | Bn-scaff_15763_1-p573751 | 54.545 |
| C06 | Bn-scaff_18206_3-p62755 | 57.429 |
| C06 | Bn-scaff_18206_2-p114119 | 57.589 |
| C06 | Bn-scaff_18206_3-p49133 | 58.296 |
| C06 | Bn-scaff_18206_1-p454050 | 59.387 |
| C06 | Bn-scaff_18206_1-p453711 | 60.23 |
| C06 | Bn-scaff_15818_2-p1637962 | 60.495 |
| C06 | Bn-scaff_15818_2-p1780340 | 60.511 |
| C06 | Bn-scaff_15818_2-p1555114 | 60.952 |
| C06 | Bn-scaff_15818_2-p1555112 | 61.32 |
| C06 | Bn-scaff_15818_2-p1385621 | 62.103 |
| C06 | Bn-scaff_15818_1-p345564 | 68.062 |
| C06 | Bn-scaff_15818_1-p363620 | 68.555 |
| C06 | Bn-scaff_15818_1-p390572 | 68.583 |
| C06 | Bn-scaff_15818_1-p297955 | 70.245 |
| C06 | Bn-scaff_15818_1-p237481 | 71.012 |
| C06 | Bn-scaff_17867_1-p27331 | 72.145 |
| C06 | Bn-scaff_28522_1-p34164 | 72.69 |
| C06 | Bn-scaff_18439_1-p798756 | 76.125 |
| C06 | Bn-scaff_20773_1-p481244 | 89.502 |
| C06 | Bn-scaff_20773_1-p463095 | 90.291 |
| C06 | Bn-scaff_16485_1-p668116 | 99.46 |
| C06 | Bn-scaff_16485_1-p127229 | 101.263 |
| C06 | Bn-scaff_16984_1-p188161 | 101.66 |
| C06 | Bn-scaff_20866_1-p58663 | 102.031 |
| C06 | Bn-scaff_16485_1-p53130 | 102.21 |
| C06 | Bn-scaff_16984_1-p204543 | 102.999 |
| C06 | Bn-scaff_16984_1-p243678 | 103.032 |
| C06 | Bn-scaff_16984_1-p324673 | 104.38 |
| C06 | Bn-scaff_16984_1-p332325 | 104.699 |
| C06 | Bn-scaff_18140_1-p39519 | 105.229 |
| C06 | Bn-A06-p2405022 | 108.26 |
| C06 | Bn-scaff_18140_1-p816795 | 109.69 |
| C06 | Bn-scaff_18140_1-p816500 | 109.719 |
| C06 | Bn-scaff_18140_1-p821898 | 109.724 |
| C06 | Bn-scaff_18140_1-p827515 | 109.776 |
| C06 | Bn-scaff_18140_1-p596899 | 111.248 |
| C06 | Bn-scaff_18549_1-p451733 | 112.481 |
| C07 | Bn-scaff_23400_1-p57185 | 0 |
| C07 | Bn-scaff_16100_1-p617160 | 2.055 |
| C07 | Bn-scaff_18501_1-p470034 | 4.316 |
| C07 | Bn-scaff_18202_1-p1536412 | 9.018 |
| C07 | Bn-scaff_18202_1-p379677 | 16.022 |
| C07 | Bn-scaff_18202_1-p377855 | 16.038 |
| C07 | Bn-scaff_18202_1-p205980 | 16.039 |
| C07 | Bn-scaff_18202_1-p719656 | 16.206 |
| C07 | Bn-scaff_18202_1-p138304 | 18.57 |
| C07 | Bn-scaff_18202_1-p136363 | 18.573 |
| C07 | Bn-scaff_22821_1-p13976 | 18.875 |
| C07 | Bn-scaff_15712_1-p204886 | 19.404 |
| C07 | Bn-scaff_19724_1-p314829 | 21.941 |
| C07 | Bn-scaff_15754_1-p243099 | 23.688 |
| C07 | Bn-scaff_15754_1-p240930 | 23.699 |
| C07 | Bn-scaff_15754_1-p243179 | 23.706 |
| C07 | Bn-scaff_15754_1-p1456373 | 29.112 |
| C07 | Bn-scaff_16130_1-p237103 | 31.813 |
| C07 | Bn-scaff_16130_1-p224821 | 31.988 |
| C07 | Bn-scaff_16130_1-p280914 | 32.445 |
| C07 | Bn-scaff_16130_1-p297843 | 32.888 |
| C07 | Bn-scaff_16130_1-p804094 | 33.746 |
| C07 | Bn-scaff_16130_1-p659049 | 34.335 |
| C07 | Bn-scaff_16130_1-p916846 | 34.911 |
| C07 | Bn-scaff_16130_1-p910136 | 34.993 |
| C07 | Bn-scaff_16130_1-p1028240 | 35.835 |
| C07 | Bn-scaff_16130_1-p1036271 | 36.436 |
| C07 | Bn-scaff_16130_1-p1042526 | 36.462 |
| C08 | Bn-scaff_17257_1-p98652 | 0 |
| C08 | Bn-scaff_18275_1-p1379444 | 0.497 |
| C08 | Bn-scaff_21250_1-p270107 | 0.917 |
| C08 | Bn-scaff_16545_1-p198332 | 1.143 |
| C08 | Bn-scaff_16545_1-p256454 | 1.583 |
| C08 | Bn-scaff_16545_1-p238397 | 1.584 |
| C08 | Bn-scaff_16545_1-p77503 | 1.64 |
| C08 | Bn-scaff_16545_1-p90406 | 1.679 |
| C08 | Bn-scaff_19104_1-p549430 | 2.154 |
| C08 | Bn-scaff_26506_1-p292189 | 2.207 |
| C08 | Bn-scaff_17257_1-p154169 | 2.497 |
| C08 | Bn-scaff_21250_1-p448924 | 2.696 |
| C08 | Bn-scaff_26506_1-p42166 | 3.179 |
| C08 | Bn-scaff_16361_1-p727512 | 44.6 |
| C08 | Bn-scaff_16361_1-p1282832 | 46.159 |
| C08 | Bn-scaff_16361_1-p889103 | 46.294 |
| C08 | Bn-scaff_16361_1-p930064 | 47.197 |
| C08 | Bn-scaff_16361_1-p900511 | 47.273 |
| C08 | Bn-scaff_16361_1-p686911 | 47.559 |
| C08 | Bn-scaff_16361_1-p1343410 | 48.874 |
| C08 | Bn-scaff_16361_1-p1331105 | 49.1 |
| C08 | Bn-scaff_16361_1-p1749058 | 49.655 |
| C08 | Bn-scaff_16361_1-p1466598 | 50.369 |
| C08 | Bn-scaff_16361_1-p1467945 | 50.404 |
| C08 | Bn-scaff_16361_1-p2529346 | 56.069 |
| C08 | Bn-scaff_16361_1-p2353581 | 56.232 |
| C08 | Bn-scaff_16361_1-p2541607 | 56.277 |
| C08 | Bn-scaff_16361_1-p2730705 | 60.6 |
| C08 | Bn-scaff_16361_1-p2750581 | 61.181 |
| C08 | Bn-scaff_16197_1-p3259829 | 67.114 |
| C08 | Bn-scaff_16197_1-p3061092 | 68.002 |
| C08 | Bn-scaff_16197_1-p3061966 | 68.267 |
| C08 | Bn-A09-p30358198 | 69.279 |
| C08 | Bn-scaff_16197_1-p3025482 | 69.59 |
| C08 | Bn-scaff_16197_1-p2891250 | 70.965 |
| C08 | Bn-scaff_16197_1-p2881744 | 71.175 |
| C08 | Bn-scaff_16197_1-p2872723 | 71.412 |
| C08 | Bn-scaff_16197_1-p3155696 | 71.458 |
| C08 | Bn-scaff_16197_1-p3070176 | 71.497 |
| C08 | Bn-scaff_16197_1-p841813 | 82.034 |
| C08 | Bn-scaff_16197_1-p784797 | 82.665 |
| C08 | Bn-scaff_16197_1-p787401 | 83.213 |
| C08 | Bn-scaff_16197_1-p732813 | 83.215 |
| C08 | Bn-scaff_16197_1-p561660 | 84.596 |
| C08 | Bn-scaff_16197_1-p689617 | 84.89 |
| C08 | Bn-scaff_16197_1-p567002 | 85.682 |
| C08 | Bn-scaff_16197_1-p135491 | 88.586 |
| C08 | Bn-scaff_16445_1-p2528516 | 90.785 |
| C08 | Bn-scaff_16445_1-p2413979 | 92.375 |
| C08 | Bn-scaff_16445_1-p1674687 | 99.112 |
| C08 | Bn-scaff_16445_1-p1639969 | 99.599 |
| C08 | Bn-scaff_16445_1-p1531740 | 101.361 |
| C08 | Bn-scaff_16445_1-p1409861 | 102.322 |
| C08 | Bn-scaff_16445_1-p1443287 | 102.374 |
| C08 | Bn-scaff_16445_1-p1460133 | 102.596 |
| C08 | Bn-scaff_16445_1-p1460618 | 102.625 |
| C08 | Bn-scaff_16445_1-p1391219 | 103.29 |
| C08 | Bn-scaff_16445_1-p1035526 | 107.625 |
| C08 | Bn-scaff_16445_1-p898556 | 107.847 |
| C08 | Bn-scaff_16445_1-p895858 | 107.995 |
| C08 | Bn-scaff_16445_1-p699436 | 109.371 |
| C08 | Bn-scaff_16445_1-p588681 | 109.395 |
| C08 | Bn-scaff_16445_1-p611520 | 109.415 |
| C08 | Bn-scaff_16445_1-p552942 | 109.451 |
| C08 | Bn-scaff_16445_1-p437285 | 110.852 |
| C08 | Bn-scaff_16445_1-p363187 | 111.128 |
| C08 | Bn-scaff_16445_1-p414675 | 111.136 |
| C08 | Bn-scaff_16445_1-p351646 | 111.512 |
| C08 | Bn-scaff_16445_1-p796374 | 111.727 |
| C08 | Bn-scaff_16445_1-p800267 | 111.89 |
| C08 | Bn-scaff_16389_1-p771131 | 123.03 |
| C08 | Bn-scaff_16389_1-p740326 | 123.303 |
| C08 | Bn-scaff_16389_1-p688405 | 123.495 |
| C08 | Bn-scaff_16389_1-p781261 | 123.988 |
| C08 | Bn-scaff_16389_1-p842667 | 124.993 |
| C08 | Bn-scaff_16021_1-p232190 | 127.417 |
| C09 | Bn-scaff_20168_1-p12226 | 0 |
| C09 | Bn-scaff_20168_1-p11649 | 0.224 |
| C09 | Bn-scaff_17888_1-p378622 | 1.942 |
| C09 | Bn-scaff_17888_1-p397760 | 1.949 |
| C09 | Bn-scaff_17801_1-p203055 | 2.448 |
| C09 | Bn-scaff_17801_1-p314822 | 2.477 |
| C09 | Bn-scaff_17801_1-p233578 | 2.485 |
| C09 | Bn-scaff_17801_1-p177923 | 2.655 |
| C09 | Bn-scaff_17801_1-p178058 | 2.725 |
| C09 | Bn-scaff_15650_1-p924603 | 4.353 |
| C09 | Bn-scaff_17996_1-p16482 | 4.415 |
| C09 | Bn-scaff_20903_1-p453556 | 4.42 |
| C09 | Bn-scaff_20903_1-p455537 | 4.683 |
| C09 | Bn-scaff_20903_1-p275471 | 4.82 |
| C09 | Bn-scaff_20903_1-p470430 | 5.369 |
| C09 | Bn-scaff_17996_1-p206180 | 6.148 |
| C09 | Bn-scaff_15650_1-p750450 | 6.475 |
| C09 | Bn-scaff_15650_1-p666957 | 6.907 |
| C09 | Bn-scaff_15650_1-p683404 | 6.941 |
| C09 | Bn-scaff_23293_1-p197240 | 10.279 |
| C09 | Bn-scaff_23293_1-p188258 | 10.393 |
| C09 | Bn-scaff_23293_1-p145877 | 11.229 |
| C09 | Bn-scaff_17109_1-p373778 | 12.678 |
| C09 | Bn-scaff_20354_1-p179456 | 13.388 |
| C09 | Bn-scaff_18424_1-p509715 | 14.452 |
| C09 | Bn-scaff_15743_1-p26515 | 19.855 |
| C09 | Bn-scaff_15808_1-p525268 | 20.359 |
| C09 | Bn-scaff_25457_1-p277536 | 20.466 |
| C09 | Bn-scaff_25457_1-p306251 | 20.496 |
| C09 | Bn-scaff_25457_1-p297013 | 20.509 |
| C09 | Bn-scaff_15808_1-p647937 | 20.537 |
| C09 | Bn-scaff_15808_1-p583082 | 20.553 |
| C09 | Bn-scaff_15808_1-p433195 | 20.762 |
| C09 | Bn-scaff_15808_1-p425512 | 20.768 |
| C09 | Bn-scaff_15808_1-p442300 | 20.811 |
| C09 | Bn-scaff_17367_1-p378846 | 22.282 |
| C09 | Bn-scaff_17367_1-p359355 | 22.312 |
| C09 | Bn-scaff_17367_1-p696709 | 22.313 |
| C09 | Bn-scaff_17367_1-p690899 | 22.344 |
| C09 | Bn-scaff_17367_1-p590827 | 22.36 |
| C09 | Bn-scaff_17367_1-p558439 | 22.391 |
| C09 | Bn-scaff_17367_1-p42800 | 22.687 |
| C09 | Bn-scaff_17367_1-p204838 | 22.702 |
| C09 | Bn-scaff_17367_1-p67748 | 22.717 |
| C09 | Bn-scaff_20619_1-p134778 | 23.156 |
| C09 | Bn-scaff_20619_1-p212447 | 23.168 |
| C09 | Bn-scaff_20619_1-p112877 | 23.317 |
| C09 | Bn-scaff_19661_1-p165828 | 23.947 |
| C09 | Bn-scaff_20619_1-p167887 | 24.106 |
| C09 | Bn-scaff_16297_1-p308218 | 24.511 |
| C09 | Bn-scaff_15808_1-p404842 | 26.203 |
| C09 | Bn-scaff_15808_1-p433123 | 26.246 |
| C09 | Bn-scaff_15808_1-p430070 | 26.269 |
| C09 | Bn-scaff_15808_1-p647845 | 26.542 |
| C09 | Bn-scaff_15808_1-p584599 | 26.592 |
| C09 | Bn-scaff_25457_1-p279509 | 26.96 |
| C09 | Bn-scaff_25457_1-p276638 | 27.008 |
| C09 | Bn-scaff_25457_1-p282237 | 27.092 |
| C09 | Bn-scaff_23208_1-p9844 | 27.258 |
| C09 | Bn-scaff_25457_1-p282637 | 27.597 |
| C09 | Bn-scaff_25457_1-p307463 | 27.686 |
| C09 | Bn-scaff_25457_1-p283396 | 27.776 |
| C09 | Bn-scaff_15743_1-p26884 | 28.255 |
| C09 | Bn-scaff_15743_1-p26527 | 28.341 |
| C09 | Bn-scaff_23096_1-p364514 | 29.988 |
| C09 | Bn-scaff_23622_1-p3989 | 30.343 |
| C09 | Bn-scaff_19490_1-p35288 | 31.099 |
| C09 | Bn-scaff_19899_1-p623253 | 35.719 |
| C09 | Bn-scaff_19899_1-p652713 | 36.19 |
| C09 | Bn-scaff_19899_1-p356624 | 36.656 |
| C09 | Bn-scaff_19899_1-p719873 | 36.806 |
| C09 | Bn-scaff_15576_1-p89263 | 40.37 |
| C09 | Bn-scaff_15576_1-p69107 | 40.862 |
| C09 | Bn-scaff_15576_1-p539638 | 42.411 |
| C09 | Bn-scaff_15576_1-p540624 | 42.731 |
| C09 | Bn-scaff_15576_1-p837802 | 42.966 |
| C09 | Bn-scaff_15576_1-p663482 | 43.048 |
| C09 | Bn-scaff_15576_1-p660538 | 43.191 |
| C09 | Bn-scaff_24057_1-p246 | 43.227 |
| C09 | Bn-scaff_27705_1-p179294 | 43.757 |
| C09 | Bn-scaff_17028_1-p277663 | 47.833 |
| C09 | Bn-A10-p12810049 | 48.721 |
| C09 | Bn-scaff_17028_1-p432707 | 49.027 |
| C09 | Bn-scaff_21841_1-p26836 | 49.094 |
| C09 | Bn-scaff_21841_1-p32983 | 49.126 |
| C09 | Bn-A10-p12901822 | 49.655 |
| C09 | Bn-scaff_21841_1-p188116 | 49.69 |

# Table S2A. List of all detected QTLs at both significant and suggestive level for seed number per pod.

| **Identified QTL** | **Experiments** | **Linkage groups** | **Position** | **Interval** | **Additive effect** | **LOD value** | ***R*^2^ (%)** | **Level** |
| --- | --- | --- | --- | --- | --- | --- | --- | --- |
| qSN01 | Z11RIL | A02 | 4.21 | 3.0-5.0 | 2.16 | 16.68 | 24.75 | Significant |
| Delelte | Z12RIL | A02 | 0.61 | 0.0-0.9 | 0.83 | 2.85 | 4.30 | Suggestive |
| qSN03 | W11RIL | A06 | 122.61 | 112.4-133.3 | 1.50 | 13.99 | 22.42 | Significant |
| qSN04 | Z11RIL | A06 | 122.61 | 113.0-136.9 | 1.54 | 10.78 | 16.28 | Significant |
| qSN05 | W12RIL | A06 | 122.61 | 118.9-134.1 | 1.80 | 10.88 | 22.90 | Suggestive |
| qSN06 | Z12RIL | A06 | 122.61 | 118.5-133.7 | 1.50 | 10.95 | 18.66 | Significant |
| qSN07 | W11RIL | C01 | 0.01 | 0.0-3.4 | -0.77 | 4.31 | 6.24 | Significant |
| qSN08 | Z11RIL | C04 | 129.61 | 129.2-129.6 | -0.87 | 4.11 | 5.30 | Significant |
| qSN09 | W11RIL | C06 | 54.51 | 53.8-56.4 | 0.97 | 6.50 | 9.86 | Significant |
| qSN10 | Z11RIL | C06 | 54.51 | 50.7-58.2 | 0.95 | 4.98 | 6.51 | Significant |
| Delelte | W12RIL | C7 | 9.01 | 5.5-13.0 | 1.07 | 3.74 | 7.90 | Suggestive |

# Table S2B. List of consensus-QTL after the integration of reproducible identified-QTLs for seed number per pod

| **Consensus QTL** | **Identified QTL** | **Experiment code** | **Linkage group** | **Peak Position** | **Confidence Interval** | **Additive effect** | **LOD value** | ***R*^2^ (%)** |
| --- | --- | --- | --- | --- | --- | --- | --- | --- |
|  |  |  |  |  |  |  |  |  |
| *qSN.A02* | qSN01 | Z11RIL | A02 | 4.21 | 3.0-5.0 | 2.16 | 16.68 | 24.75 |
|  | qSN03 | W11RIL | A06 | 122.61 | 113.5-133.3 | 1.50 | 13.99 | 22.42 |
|  | qSN04 | Z11RIL | A06 | 122.61 | 113.7-132.9 | 1.54 | 10.78 | 16.28 |
|  | qSN05 | W12RIL | A06 | 122.61 | 118.9-134.1 | 1.80 | 10.88 | 22.90 |
|  | qSN06 | Z12RIL | A06 | 122.61 | 118.5-133.7 | 1.50 | 10.95 | 18.66 |
| *qSN.A06* |  | W11RIL/Z11RIL/W12RIL/Z12RIL | A06 | 122.61 | 113.5-133.7 | 1.58 | 11.65 | 20.07 |
| *qSN.C01* | qSN07 | W11RIL | C01 | 0.01 | 0.0-3.4 | -0.77 | 4.31 | 6.24 |
| *qSN.C04* | qSN08 | Z11RIL | C04 | 129.61 | 129.2-129.6 | -0.87 | 4.11 | 5.30 |
|  | qSN09 | W11RIL | C06 | 54.51 | 53.8-56.4 | 0.97 | 6.50 | 9.86 |
|  | qSN10 | Z11RIL | C06 | 54.51 | 50.7-58.2 | 0.95 | 4.98 | 6.51 |
| *qSN.C06* |  | W11RIL/Z11RIL | C06 | 54.51 | 50.7-58.2 | 0.96 | 5.74 | 8.18 |

# Table S3. Main information of QTLs identified in the current and previous studies for SNPP in rapeseed

| Referecne | Mapping strategy | Trait | QTL name | Linkage group | Marker interval | Genomic regions (Mb) | LOD value | ***R*^2^** | Additive effect | Experiments | |
| --- | --- | --- | --- | --- | --- | --- | --- | --- | --- | --- | --- |
| Shi et al. 2015 | Linkage mapping | SNPP | qSN.A01-1 | A01 | BoSF2770 / niab106 | 0.5-1.5 | 4.21 | 8.79 | -1.01 | 1 |  |
| Shi et al. 2009 | Linkage mapping | SNPPw | qSN.A1-1 | A01 | niab71 / CNU142 | 0.7-1.5 | 2.4-3.9 | 4-6.1 | ± | 2 |  |
| Shi et al. 2009 | Linkage mapping | SNPPw | qSN.A1-2 | A01 | niab096 / EST156a | 2.0-2.1 | 2.7-8.1 | 4.7-12.2 | + | 2 |  |
| Shi et al. 2009 | Linkage mapping | SNPPw | qSN.A1-3 | A01 | sR11990a / RA2G09 | 4.0-5.5 | 2.5-2.8 | 5.5-5.8 | + | 2 |  |
| Shi et al. 2009 | Linkage mapping | SNPPw | qSN.A1-4 | A01 | E5HM31-505 / S10M16-2-130 | /-/ | 4.9-6.7 | 8.8-11.4 | + | 2 |  |
| Shi et al. 2009 | Linkage mapping | SNPPw | qSN.A1-5 | A01 | CNU235 / Ol12F11 | 6.6-7.0 | 5.5-7.1 | 9.5-10 | + | 2 |  |
| Shi et al. 2009 | Linkage mapping | SNPPw | qSN.A1-7 | A01 | E10HM34-230 / EST165 | /-8.7-/ | 4.3-5.5 | 4.2-9.7 | + | 2 |  |
| Qi et al. 2014 | Linkage mapping | SNPPm | qSNA1 | A01 | Na14G06 / CB10189 | 8.2-8.9 | / | 23.54 | 1.93 | 3 | Major QTL |
| Shi et al. 2009 | Linkage mapping | SNPPw | qSN.A1-6 | A01 | IGF2071e / EST98b | /-17.5 | 3-9.1 | 4.3-12.4 | + | 5 |  |
| Shi et al. 2015 | Linkage mapping | SNPP | qSN.A01-2 | A01 | TFZIP23 / BrSF0982 | 18.3-19.6 | 3.71 | 2.73 | -0.89 | 2 |  |
| Wang et al. 2010 | Linkage mapping | SNPPw | qSS1 | A01 | ME6EM8d / ME3EM8a | /-/ | 2.93 | 8.41 | −3.6 | 1 |  |
| Current study-BnaZNRIL | Linkage mapping | SNPPm | qSN.A02 | A02 | Bn-A02-p2788144/Bn-A02-p2851231 | 0.3-0.4 | 16.70 | 24.80 | 2.16 | 1 |  |
| Shi et al. 2015 | Linkage mapping | SNPP | qSN.A02-1 | A02 | niab025 / MR052 | 0.1-1.8 | 4.70 | 5.31 | 0.36 | 1 |  |
| Shi et al. 2013 | Linkage mapping | SNPPw | qSN-OP1-A2b/qSN-OP2-A2 | A02 | BnSIZ1-A2 / BnMGD2-A2b | 2.0-3.0 | 7.90 | 14.45 | - | 2 |  |
| Shi et al. 2013 | Linkage mapping | SNPPw | qSN-OP3-A2/qSN-OP1-A2a/qSN-LP3-A2 | A02 | sR6293a / BnSIZ1-A2 | 3.0-4.7 | 4.47 | 9.37 | - | 3 |  |
| Ding et al. 2012 | Linkage mapping | SNPPw | SNLP-A2 | A02 | Na14H11 / B049H14 | 4.2-4.5 | / | 11.20 | - | 1 |  |
| Shi et al. 2015 | Linkage mapping | SNPP | qSN.A02-2 | A02 | BrSF0755 / BoSF2100 | 6.0-8.3 | 5.84 | 7.04 | 1.11 | 3 |  |
| Shi et al. 2009 | Linkage mapping | SNPPw | qSN.A2-1 | A02 | pX154 / pX155 | 10.2-10.3 | 10.20 | 15.50 | -1.81 | 1 |  |
| Shi et al. 2013 | Linkage mapping | SNPPw | qSN-LP1-A2a/qSN-LP1-A2b/qSN-LP2-A2 | A02 | ZAAS113 / B084M08a | 10.3-13.1 | 3.74 | 6.79 | - | 3 |  |
| Shi et al. 2015 | Linkage mapping | SNPP | qSN.A02-3 | A02 | BoSF2214 / Na14B05 | 22.4-22.7 | 4.23 | 4.27 | 0.32 | 2 |  |
| Shi et al. 2009 | Linkage mapping | SNPPw | qSN.A2-2 | A02 | S10M16-2-210 / S10M03-1-280 | /-/ | 3.00 | 5.50 | -0.98 | 1 |  |
| Shi et al. 2015 | Linkage mapping | SNPP | qSN.A03-1 | A03 | BrSF0427 / BoSF2392a | 1.5-4.1 | 4.30 | 16.32 | -1.73 | 1 |  |
| Shi et al. 2013 | Linkage mapping | SNPPw | qSN-LP3-A3b | A03 | HBr049 / CNU098 | 12.8-13.5 | 4.20 | 8.00 | + | 1 |  |
| Shi et al. 2009 | Linkage mapping | SNPPw | qSN.A3-1 | A03 | CNU210 / S13M08-1-157 | 15.3-15.7 | 2.3-3.9 | 4.5-5.6 | ± | 2 |  |
| Ding et al. 2012 | Linkage mapping | SNPPw | SNLP-A3 | A03 | CNU270 / HBr131 | 15.4-16.2 | / | 11.40 | - | 1 |  |
| Shi et al. 2015 | Linkage mapping | SNPP | qSN.A03-2 | A03 | BrSF0708 / CB10425 | 23.3-24.6 | 3.13 | 4.30 | -0.82 | 1 |  |
| Ding et al. 2012 | Linkage mapping | SNPPw | SNNP-A3 | A03 | BoGMS1539 / BoGMS0408 | 25.5-27.3 | / | 16.10 | + | 1 |  |
| Shi et al. 2009 | Linkage mapping | SNPPw | qSN.A4-2 | A04 | IGF3395d / IGF3391a | 0.0-0.5 | 6.7-7.5 | 8.5-12.1 | - | 2 |  |
| Shi et al. 2009 | Linkage mapping | SNPPw | qSN.A4-3 | A04 | HUA39 / CNU360 | 15.9-16.2 | 2.7-3 | 5.6-6.1 | + | 2 |  |
| Shi et al. 2009 | Linkage mapping | SNPPw | qSN.A4-4 | A04 | CNU256 / E9HM31-80 | 16.2-17.2 | 4.5-8.1 | 7.7-13.2 | + | 2 |  |
| Shi et al. 2009 | Linkage mapping | SNPPw | qSN.A4-5 | A04 | E10HM34-180 / S14M08-2-270 | /-/ | 3-3.3 | 3.7-4.1 | ± | 2 |  |
| Shi et al. 2009 | Linkage mapping | SNPPw | qSN.A4-1 | A04 | E6HM31-400 / P7M1-275 | /-/ | 3.3-7 | 6.1-14 | + | 2 |  |
| Shi et al. 2009 | Linkage mapping | SNPPw | qSN.A5-1 | A05 | CNU257 / BRAS063 | 3.2-3.3 | 3.90 | 7.60 | -0.54 | 1 |  |
| Shi et al. 2015 | Linkage mapping | SNPP | qSN.A05-1 | A05 | BrSF0712 / BoSF2141 | 5.8-7.8 | 4.13 | 4.75 | -1.03 | 3 |  |
| Radoev et al. 2008 | Linkage mapping | SNPPm | S/Sil_N5 | A05 | MD21 / MR113 | 8.0-12.4 | 14.10 | 11.80 | -1.55 | 1 |  |
| Shi et al. 2009 | Linkage mapping | SNPPw | qSN.A5-2 | A05 | CNU206 / pW161-2b | 11.2-12.8 | 3.90 | 6.00 | -0.78 | 1 |  |
| Wang et al. 2010 | Linkage mapping | SNPPw | qSS5 | A05 | ME1EM6b / ME7EM6c | /-/ | 2.41 | 7.87 | −5.38 | 1 |  |
| Current study-BnaZNRIL | Linkage mapping | SNPPm | qSN.A06 | A06 | Bn-A06-p22608192/Bn-A06-p24274213 | 21.6-23.3 | 11.60 | 20.10 | 1.58 | 4 | Major QTL |
| Shi et al. 2015 | Linkage mapping | SNPP | qSN.A06-1 | A06 | BrSF0819 / BrSF0144 | 22.6-23.7 | 10.99 | 32.05 | 1.97 | 13 | Major QTL |
| Cai et al. 2014 | Association mapping | SNPPm | UN | A06 | BnGMS583 | /-16.8-/ | 3.63 | 4.78 | 0.73 | 1 |  |
| Ding et al. 2012 | Linkage mapping | SNPPw | SNLP-A6 | A06 | BnGS1 / em13me5 | /-/ | / | 13.00 | + | 2 |  |
| Shi et al. 2009 | Linkage mapping | SNPPw | qSN.A7-1 | A07 | CNU063 / RA2G08 | 0.8-5.2 | 3.30 | 5.60 | 0.62 | 1 |  |
| Shi et al. 2015 | Linkage mapping | SNPP | qSN.A07-1 | A07 | BoSF2344 / AT1G27050 | 5.0-8.9 | 5.86 | 6.94 | 0.75 | 2 |  |
| Cai et al. 2014 | Association mapping | SNPPm | UN | A07 | BrGMS4507 | /-10.3-/ | 3.14 | 5.22 | 1.38 | 1 |  |
| Shi et al. 2015 | Linkage mapping | SNPP | qSN.A07-2 | A07 | CN64 / CNU339 | 15.0-20.1 | 6.00 | 10.77 | 0.86 | 4 |  |
| Shi et al. 2015 | Linkage mapping | SNPP | qSN.A07-3 | A07 | BoGMS2110 / CNU053 | 20.8-22.5 | 3.89 | 3.84 | 0.48 | 3 |  |
| Qi et al. 2014 | Linkage mapping | SNPPm | qSNA7 | A07 | T10 / EA2MG2a | /-/ | / | 3.75 | 0.77 | 3 |  |
| Zhang et al. 2011 | Linkage mapping | SNPPm | qSS.N8-1 | A08 | EA02MG05-210 / CB10026 | 7.2-9.2 | 4.38 | 12.23 | -3.10 | 3 |  |
| Ding et al. 2012 | Linkage mapping | SNPPw | SNLP-A8 | A08 | H012K23 / BnPAP25 | 12.0-12.7 | / | 9.40 | + | 1 |  |
| Qi et al. 2014 | Linkage mapping | SNPPm | qSNA8 | A08 | niab050 / Lat21-2 | 16.5-17.6 | / | 5.51 | -0.93 | 3 |  |
| Shi et al. 2009 | Linkage mapping | SNPPw | qSN.A8-1 | A08 | CNU489 / P7M5-170 | /-/ | 2.6-3.3 | 4.4-5.2 | - | 2 |  |
| Zhang et al. 2011 | Linkage mapping | SNPPm | qSS.N8-3 | A08 | SA06TC14-270 / S77096 | /-/ | 2.90 | 9.63 | 2.73 | 1 |  |
| Zhang et al. 2011 | Linkage mapping | SNPPm | qSS.N8-4 | A08 | SA06TC14-270 / S77096 | /-/ | 2.52 | 8.35 | 2.83 | 2 |  |
| Zhang et al. 2011 | Linkage mapping | SNPPm | qSS.N8-2 | A08 | SA10TC11-100 / SA03TC09-370 | /-/ | 2.92 | 10.60 | 4.07 | 3 |  |
| Shi et al. 2009 | Linkage mapping | SNPPw | qSN.A9-1 | A09 | P04M21-130 / IGF5222b | 1.0-1.8 | 4.10 | 6.50 | 0.77 | 1 |  |
| Ding et al. 2012 | Linkage mapping | SNPPw | SNNP-A9 | A09 | HBr199c / HBr097c | 1.2-1.8 | / | 10.50 | - | 2 |  |
| Shi et al. 2013 | Linkage mapping | SNPPw | qSN-LP1-A9b | A09 | IGF5385f / IGF5222b | 1.8-2.0 | 4.70 | 8.40 | + | 1 |  |
| Shi et al. 2009 | Linkage mapping | SNPPw | qSN.A9-2 | A09 | CB10311 / pW203b | 7.2-8.6 | 4.10 | 5.40 | 0.76 | 1 |  |
| Shi et al. 2013 | Linkage mapping | SNPPw | qSN-LP1-A9a/qSN-OP2-A9 | A09 | H055O17-4 / HBr096 | 25.1-29.8 | 3.90 | 6.85 | + | 2 |  |
| Shi et al. 2015 | Linkage mapping | SNPP | qSN.A09-1 | A09 | BnSF2342-39 / BnID082 | 28.1-29.1 | 3.55 | 4.93 | 0.59 | 2 |  |
| Shi et al. 2009 | Linkage mapping | SNPPw | qSN.A9-3 | A09 | niab33 / pW123aH | 30.0-31.2 | 3.00 | 5.60 | -0.47 | 1 |  |
| Qi et al. 2014 | Linkage mapping | SNPPm | qSNA9 | A09 | CNU034 / niab038 | /-/ | / | 2.73 | -0.66 | 3 |  |
| Current study-BnaAP | Association mapping | SNPPm |  | A10 | Bn-A10-p13038966 | 13.1 |  | 8.30 |  | 2 |  |
| Ding et al. 2012 | Linkage mapping | SNPPw | SNLP-A10 | A10 | Na10D07 / S002B15-1 | 10.5-11.5 | / | 12.80 | + | 1 |  |
| Current study-BnaZNRIL | Linkage mapping | SNPPm | qSN.C01 | C01 | Bn-scaff_15838_1-p747634/Bn-scaff_15838_1-p1218165 | 1.2-1.6 | 4.30 | 6.20 | -0.77 | 1 |  |
| Ding et al. 2012 | Linkage mapping | SNPPw | SNLP-C1 | C01 | CB10369 / BoGMS0806 | 6.3-8.3 | / | 11.90 | + | 1 |  |
| Wang et al. 2010 | Linkage mapping | SNPPw | qSS11 | C01 | Ol12F11a / ME5EM9c | 10.1-12.1 | 2.98 | 24.37 | −5.51 | 1 | Major QTL |
| Shi et al. 2013 | Linkage mapping | SNPPw | qSN-LP2-C1 | C01 | ZAAS156b / znS08M15-80 | 19.1-22.0 | 3.60 | 7.10 | - | 1 |  |
| Shi et al. 2013 | Linkage mapping | SNPPw | qSN-OP2-C1 | C01 | BnGPT2-C1 / pW190b | 28.3-29.3 | 3.50 | 7.50 | - | 1 |  |
| Shi et al. 2013 | Linkage mapping | SNPPw | qSN-OP1-C1 | C01 | HR-Sp2-170 / BRMS175 | 36.5-38.8 | 3.70 | 6.30 | - | 1 |  |
| Radoev et al. 2008 | Linkage mapping | SNPPm | S/Sil_N11 | C01 | CB10536 / CB10357b | 35.3-37.7 | 15.60 | 8.20 | -1.49 | 1 |  |
| Current study-BnaAP | Association mapping | SNPPm |  | C02 | Bn-scaff_17725_1-p430981 | 10.1 |  | 13.80 |  | 2 |  |
| Shi et al. 2015 | Linkage mapping | SNPP | qSN.C02-1 | C02 | BoSF1425 / CNU461 | 0.8-2.2 | 3.82 | 6.67 | -0.71 | 1 |  |
| Shi et al. 2009 | Linkage mapping | SNPPw | qSN.C2-1 | C02 | pW119 / sR12095 | 1.4-1.7 | 2.6-3.4 | 3.3-4.7 | + | 2 |  |
| Ding et al. 2012 | Linkage mapping | SNPPw | SNNP-C3 | C03 | BoGMS0576 / MR123 | 9.1-10.0 | / | 17.30 | + | 1 |  |
| Qi et al. 2014 | Linkage mapping | SNPPm | qSNC3 | C03 | t8_at_3 / CNU270 | 23.2-/ | / | 5.12 | 0.90 | 3 |  |
| Shi et al. 2009 | Linkage mapping | SNPPw | qSN.C3-1 | C03 | IGF1141f / E7HM31-450 | 30.3-/ | 8.60 | 10.80 | -1.43 | 1 |  |
| Zhang et al. 2011 | Linkage mapping | SNPPm | qSS.N13-2 | C03 | EA02MC05-160 / EA13MC01-160 | /-/ | 3.42 | 9.70 | -2.31 | 2 |  |
| Zhang et al. 2011 | Linkage mapping | SNPPm | qSS.N13-1 | C03 | EA02MC05-160 / EA13MC01-160 | /-/ | 5.79 | 15.21 | -3.21 | 2 |  |
| Current study-BnaZNRIL | Linkage mapping | SNPPm | qSN.C04 | C04 | Bn-scaff_16888_1-p1382480/Bn-scaff_16888_1-p1554563 | 45.5-45.7 | 4.10 | 5.30 | -0.87 | 1 |  |
| Ding et al. 2012 | Linkage mapping | SNPPw | SNNP-C5 | C05 | BnGMS352 / BnGMS309 | 15.3-16.0 | / | 11.50 | - | 1 |  |
| Ding et al. 2012 | Linkage mapping | SNPPw | SNLP-C5 | C05 | Ol10A10 / sS2129 | 24.5-25.7 | / | 14.70 | + | 1 |  |
| Current study-BnaZNRIL | Linkage mapping | SNPPm | qSN.C06 | C06 | Bn-scaff_18206_2-p114119/Bn-scaff_15763_1-p1460541 | 18.9-20.4 | 5.74 | 8.18 | 0.96 | 2 |  |
| Shi et al. 2009 | Linkage mapping | SNPPw | qSN.C6-4 | C06 | HUA64-2 / Na12E01a | 16.9-18.1 | 4.30 | 6.90 | 0.88 | 1 |  |
| Shi et al. 2015 | Linkage mapping | SNPP | qSN.C06-1 | C06 | BoSF1254 / BoSF1126 | 17.8-19.0 | 3.72 | 4.72 | 0.72 | 2 |  |
| Shi et al. 2009 | Linkage mapping | SNPPw | qSN.C6-3 | C06 | CB10010 / IGF3380b | 20.2-20.5 | 4.1-4.2 | 3.6-7.7 | + | 2 |  |
| Shi et al. 2009 | Linkage mapping | SNPPw | qSN.C6-2 | C06 | E5HM40-205 / BRMS015 | 20.6-21.1 | 3.90 | 6.80 | 0.62 | 1 |  |
| Shi et al. 2009 | Linkage mapping | SNPPw | qSN.C6-1 | C06 | pW134 / AP1a | 29.6-32.0 | 3.90 | 6.80 | 0.71 | 1 |  |
| Shi et al. 2015 | Linkage mapping | SNPP | qSN.C06-2 | C06 | BoSF1951 / CNU331a | 30.6-34.2 | 4.59 | 8.59 | 1.03 | 2 |  |
| Shi et al. 2013 | Linkage mapping | SNPPw | qSN-OP3-C6b/qSN-LP1-C6 | C06 | BnPHT1-C6 / PA28 | 34.4-36.0 | 4.15 | 8.60 | + | 2 |  |
| Qi et al. 2014 | Linkage mapping | SNPPm | qSNC6 | C06 | CNU182 / CNU052 | 34.8-35.9 | / | 0.78 | 0.35 | 3 |  |
| Shi et al. 2013 | Linkage mapping | SNPPw | qSN-LP3-C6/qSN-OP3-C6a | C06 | IGF1226b / BnPHT1-C6 | 36.1-36.9 | 3.95 | 9.20 | + | 2 |  |
| Wang et al. 2010 | Linkage mapping | SNPPw | qSS16a | C06 | ME6EM4d / ME3EM4b | /-/ | 2.60 | 8.57 | −5.85 | 1 |  |
| Wang et al. 2010 | Linkage mapping | SNPPw | qSS16b | C06 | ME6EM4d / ME3EM4b | /-/ | 3.65 | 14.31 | −6.15 | 1 |  |
| Shi et al. 2009 | Linkage mapping | SNPPw | qSN.C8-2 | C08 | IGF3369a / MR64 | 28.8-31.4 | 5.50 | 5.10 | 0.73 | 1 |  |
| Shi et al. 2009 | Linkage mapping | SNPPw | qSN.C8-3 | C08 | sN11670a / IGF2529z | 31.8-33.6 | 5.3-7.4 | 5.4-7.8 | + | 2 |  |
| Shi et al. 2009 | Linkage mapping | SNPPw | qSN.C8-4 | C08 | IGF2529z / pW123cE | 33.6-35.3 | 4.60 | 4.30 | 0.39 | 1 |  |
| Shi et al. 2009 | Linkage mapping | SNPPw | qSN.C8-1 | C08 | S15M04-2-100 / Ol12G04 | /-/ | 3.4-5.4 | 5.1-5.2 | - | 2 |  |
| Shi et al. 2009 | Linkage mapping | SNPPw | qSN.C9-2 | C09 | E2HM34-260 / Na12G04 | 33.2-34.0 | 2.6-6.4 | 4.3-8.6 | - | 6 |  |
| Zhang et al. 2011 | Linkage mapping | SNPPm | qSS.N19 | C09 | EA13MC03-150 / Na10D07 | 39.4-/ | 2.47 | 6.11 | 2.07 | 1 |  |
| Shi et al. 2009 | Linkage mapping | SNPPw | qSN.C9-3 | C09 | IGF5193b / CB10288 | 39.0-40.0 | 2.3-5.9 | 3.2-8.1 | - | 6 |  |
| Zhang et al. 2012 | Linkage mapping | SNPPm | qSS.C9 | C09 | SRC9-022 / SA09MC04 | 44.6-46.0 | 22.50 | 57.00 | 5.40 | 3 | Major QTL |
| Radoev et al. 2008 | Linkage mapping | SNPPm | S/Sil_N19 | C09 | CB10345 / Na10B11b | 11.1-12.4 | 6.10 | 3.60 | 0.99 | 1 |  |
| Shi et al. 2009 | Linkage mapping | SNPPw | qSN.C9-1 | C09 | SA30 / S15M04-2-92 | /-/ | 2.7-5.8 | 3.4-7.9 | - | 4 |  |
| Cai et al. 2014 | Association mapping | SNPPm | / | / | EA05MC08_8 | /-/ | 3.07 | 3.96 | 0.78 | 1 |  |
| Cai et al. 2014 | Association mapping | SNPPm | / | / | EA06MC09_10 | /-/ | 3.45 | 4.44 | 1.35 | 1 |  |
| Cai et al. 2014 | Association mapping | SNPPm | / | / | EA06MG08_9 | /-/ | 3.02 | 3.85 | 2.11 | 1 |  |
| Cai et al. 2014 | Association mapping | SNPPm | / | / | EA06MG10_5 | /-/ | 3.63 | 4.70 | 1.45 | 1 |  |
| Cai et al. 2014 | Association mapping | SNPPm | / | / | EA08MC10_5 | /-/ | 3.45 | 4.44 | 1.10 | 1 |  |

# Table S4. The alignment of candidate genes from model species using the silico mapping approach

| No. | Linkage group | Position-Start (bp) | Position-End (bp) | Gene name | Gene model | Traits related |
| --- | --- | --- | --- | --- | --- | --- |
| 1 | chrA01 | 754862 | 756492 | *EDA2* | AT2G18080 | Embryo Sac Development |
| 2 | chrA01 | 1431594 | 1434149 | *EDA9* | AT4G34200 | Embryo Sac Development |
| 3 | chrA01 | 2077388 | 2085923 | *LUG* | AT4G32551 | Ovule Development |
| 4 | chrA01 | 2527747 | 2528126 | *EC1.4* | AT4G39340 | Double Fertilization |
| 5 | chrA01 | 2527798 | 2528021 | *EC1.3* | AT2G21750 | Double Fertilization |
| 6 | chrA01 | 2886712 | 2888010 | *NFD1* | AT4G30930 | Embryo Sac Development |
| 7 | chrA01 | 3353600 | 3356085 | *ALT2* | AT4G29860 | ovlue development |
| 8 | chrA01 | 4692265 | 4693993 | *ATMYB98* | AT4G18770 | Embryo Sac Development |
| 9 | chrA01 | 4799295 | 4803248 | *AG* | AT4G18960 | Ovule Development |
| 10 | chrA01 | 5205480 | 5209381 | *EDA23* | AT5G44700 | Embryo Sac Development |
| 11 | chrA01 | 5785931 | 5789004 | *ATML1* | AT4G21750 | embryo development ending in seed dormancy |
| 12 | chrA01 | 6639192 | 6639532 | *GIG1* | AT3G57860 | Embryo Sac Development |
| 13 | chrA01 | 6995979 | 7000906 | *EMB140* | AT4G24270 | embryo development ending in seed dormancy |
| 14 | chrA01 | 7218203 | 7219578 | *TTL* | AT4G24900 | embryo development ending in seed dormancy |
| 15 | chrA01 | 7248347 | 7248663 | *TPD1* | AT4G24972 | pollen development |
| 16 | chrA01 | 10387570 | 10391012 | *EDA15* | AT4G14790 | Embryo Sac Development |
| 17 | chrA01 | 13698220 | 13702078 | *CUV* | AT5G13010 | embryo development ending in seed dormancy |
| 18 | chrA01 | 15684131 | 15686523 | *UNE16* | AT4G13640 | Double Fertilization |
| 19 | chrA01 | 16779144 | 16779595 | *MIR167A* | AT3G22886 | ovlue development |
| 20 | chrA01 | 17252597 | 17253566 | *YAO* | AT4G05410 | Embryo Sac Development |
| 21 | chrA01 | 19955098 | 19955609 | *ANAC018* | AT1G52880 | ovlue development |
| 22 | chrA01 | 20660128 | 20663172 | *ABCG26* | AT3G13220 | pollen development |
| 23 | chrA01 | 21161027 | 21164861 | *SAP130A* | AT3G55200 | pollen development |
| 24 | chrA01 | 22528535 | 22531869 | *CLI1* | AT3G02130 | embryo development ending in seed dormancy |
| 25 | chrA01 | 22949878 | 22953041 | *EDA30* | AT3G03810 | Embryo Sac Development |
| 26 | chrA01 | 23004992 | 23006895 | *UNE7* | AT3G03690 | Double Fertilization |
| 27 | chrA02 | 623591 | 628622 | *CUV* | AT5G13010 | embryo development ending in seed dormancy |
| 28 | chrA02 | 742632 | 746543 | *NEF1* | AT5G13390 | pollen development |
| 29 | chrA02 | 773810 | 777717 | *FY* | AT5G13480 | embryo development ending in seed dormancy |
| 30 | chrA02 | 1089673 | 1092294 | *PIN8* | AT5G15100 | pollen development |
| 31 | chrA02 | 1369241 | 1372969 | *KAN* | AT5G16560 | ovlue development |
| 32 | chrA02 | 1441976 | 1446626 | *TOZ* | AT5G16750 | embryo development ending in seed dormancy |
| 33 | chrA02 | 1493841 | 1501268 | *CRM1B* | AT3G03110 | Embryo Sac Development |
| 34 | chrA02 | 1595320 | 1596278 | *UNE6* | AT3G03340 | Double Fertilization |
| 35 | chrA02 | 1772040 | 1773085 | *VDD* | AT5G18000 | Embryo Sac Development |
| 36 | chrA02 | 2196795 | 2197430 | *CER3* | AT5G57800 | Double Fertilization |
| 37 | chrA02 | 2306873 | 2311486 | *TSL* | AT5G20930 | Ovule Development |
| 38 | chrA02 | 2480888 | 2482970 | *RHF2A* | AT5G22000 | Embryo Sac Development |
| 39 | chrA02 | 2732315 | 2735842 | *EMB1030* | AT5G22800 | embryo development ending in seed dormancy |
| 40 | chrA02 | 3193399 | 3195490 | *ATRPP30* | AT5G59980 | Embryo Sac Development |
| 41 | chrA02 | 4664486 | 4666208 | *ATGPT1* | AT5G54800 | Embryo Sac Development |
| 42 | chrA02 | 5013634 | 5015335 | *ANAC098* | AT5G53950 | embryo development ending in seed dormancy |
| 43 | chrA02 | 5516865 | 5519398 | *ATNLE* | AT5G52820 | Embryo Sac Development |
| 44 | chrA02 | 5581374 | 5584228 | *OVA6* | AT5G52520 | ovlue development |
| 45 | chrA02 | 6356172 | 6358817 | *GLC* | AT1G65450 | Double Fertilization |
| 46 | chrA02 | 6980314 | 7002451 | *ATMDN1* | AT1G67120 | Embryo Sac Development |
| 47 | chrA02 | 7447549 | 7449067 | *ANN5* | AT1G68090 | pollen development |
| 48 | chrA02 | 8587644 | 8588045 | *EDA24* | AT1G70540 | Embryo Sac Development |
| 49 | chrA02 | 9112799 | 9114580 | *HISN6A* | AT5G10330 | embryo development ending in seed dormancy |
| 50 | chrA02 | 9441041 | 9443919 | *EDA25* | AT1G72440 | Embryo Sac Development |
| 51 | chrA02 | 9675683 | 9678735 | *EDA17* | AT1G72970 | Embryo Sac Development |
| 52 | chrA02 | 10632879 | 10633303 | *EC1.1* | AT1G76750 | Double Fertilization |
| 53 | chrA02 | 13034049 | 13035793 | *UNE12* | AT4G02590 | Double Fertilization |
| 54 | chrA02 | 15348484 | 15352220 | *EDA23* | AT5G44700 | Embryo Sac Development |
| 55 | chrA02 | 16040738 | 16041869 | *ATS* | AT5G42630 | ovlue development |
| 56 | chrA02 | 18425389 | 18427031 | *ATRAB1A* | AT5G47200 | pollen development |
| 57 | chrA02 | 18962130 | 18965006 | *EDA26* | AT2G01730 | Embryo Sac Development |
| 58 | chrA02 | 19687723 | 19689952 | *ATSPP* | AT2G03120 | pollen development |
| 59 | chrA02 | 19881235 | 19882907 | *PIN7* | AT1G23080 | embryo development ending in seed dormancy |
| 60 | chrA02 | 21810085 | 21812475 | *AACT2* | AT5G48230 | embryo development ending in seed dormancy |
| 61 | chrA02 | 22085376 | 22091694 | *OVA2* | AT5G49030 | ovlue development |
| 62 | chrA02 | 22584485 | 22586977 | *EMB161* | AT5G27740 | embryo development ending in seed dormancy |
| 63 | chrA02 | 24127938 | 24130122 | *BT1* | AT5G63160 | Embryo Sac Development |
| 64 | chrA02 | 24128459 | 24129451 | *ATBT2* | AT3G48360 | Embryo Sac Development |
| 65 | chrA03 | 90845 | 95820 | *EDA20* | AT4G00020 | Embryo Sac Development |
| 66 | chrA03 | 778889 | 789620 | *APC1* | AT5G05560 | Embryo Sac Development |
| 67 | chrA03 | 872621 | 874161 | *EDA13* | AT2G47990 | Embryo Sac Development |
| 68 | chrA03 | 874694 | 877658 | *ATO* | AT5G06160 | Embryo Sac Development |
| 69 | chrA03 | 2037095 | 2041427 | *FY* | AT5G13480 | embryo development ending in seed dormancy |
| 70 | chrA03 | 2562214 | 2564440 | *AGL2* | AT5G15800 | ovlue development |
| 71 | chrA03 | 2751783 | 2755331 | *KAN* | AT5G16560 | ovlue development |
| 72 | chrA03 | 2829328 | 2836436 | *CRM1B* | AT3G03110 | Embryo Sac Development |
| 73 | chrA03 | 2958246 | 2959247 | *UNE6* | AT3G03340 | Double Fertilization |
| 74 | chrA03 | 3762379 | 3764273 | *RHF2A* | AT5G22000 | Embryo Sac Development |
| 75 | chrA03 | 4451258 | 4452956 | *ATMSI1* | AT5G58230 | embryo development ending in seed dormancy |
| 76 | chrA03 | 4937542 | 4939591 | *ATWRKY2* | AT5G56270 | embryo development ending in seed dormancy |
| 77 | chrA03 | 5030551 | 5037104 | *WYR* | AT5G55820 | Embryo Sac Development |
| 78 | chrA03 | 5257696 | 5259390 | *ATGPT1* | AT5G54800 | Embryo Sac Development |
| 79 | chrA03 | 6633323 | 6635856 | *ATNLE* | AT5G52820 | Embryo Sac Development |
| 80 | chrA03 | 7129300 | 7130432 | *BDL* | AT1G04550 | Embryo Development |
| 81 | chrA03 | 7545217 | 7556984 | *GEM1* | AT2G35630 | pollen development |
| 82 | chrA03 | 7679403 | 7682225 | *BLH1* | AT2G35940 | Embryo Sac Development |
| 83 | chrA03 | 8296309 | 8297176 | *APD1* | AT2G38185 | pollen development |
| 84 | chrA03 | 8296465 | 8296895 | *APD3* | AT2G38220 | pollen development |
| 85 | chrA03 | 9514886 | 9517314 | *NPG1* | AT2G43040 | pollen development |
| 86 | chrA03 | 9731967 | 9738129 | *ATMAPK6* | AT2G43790 | ovlue development |
| 87 | chrA03 | 10297509 | 10299748 | *ATPDI11* | AT2G47470 | Embryo Sac Development |
| 88 | chrA03 | 10914233 | 10916380 | *UNE10* | AT4G00050 | Double Fertilization |
| 89 | chrA03 | 10929097 | 10929639 | *UNE11* | AT4G00080 | Double Fertilization |
| 90 | chrA03 | 11185509 | 11187133 | *CEP1* | AT5G50260 | pollen development |
| 91 | chrA03 | 11642522 | 11644338 | *AGL11* | AT4G09960 | ovlue development |
| 92 | chrA03 | 12774542 | 12776490 | *UNE12* | AT4G02590 | Double Fertilization |
| 93 | chrA03 | 12780382 | 12784401 | *ATCUL1* | AT4G02570 | embryo development ending in seed dormancy |
| 94 | chrA03 | 12854765 | 12855790 | *EMB2386* | AT1G02780 | Embryo Development |
| 95 | chrA03 | 12912729 | 12916693 | *PR1* | AT2G14610 | Embryo Sac Development |
| 96 | chrA03 | 13435734 | 13436367 | *EDA33* | AT4G00120 | Embryo Sac Development |
| 97 | chrA03 | 13440457 | 13443673 | *UNE10* | AT4G00050 | Double Fertilization |
| 98 | chrA03 | 13878394 | 13881651 | *EDA30* | AT3G03810 | Embryo Sac Development |
| 99 | chrA03 | 14127760 | 14129948 | *ATS6A.2* | AT3G05530 | Embryo Sac Development |
| 100 | chrA03 | 14128161 | 14129939 | *RPT5B* | AT1G09100 | Embryo Sac Development |
| 101 | chrA03 | 14170514 | 14171811 | *ATHAP2B* | AT3G05690 | Double Fertilization |
| 102 | chrA03 | 17512406 | 17517664 | *ATUBP14* | AT3G20630 | embryo development ending in seed dormancy |
| 103 | chrA03 | 17814987 | 17815855 | *SGP2* | AT3G21700 | pollen development |
| 104 | chrA03 | 18285870 | 18286229 | *EDA6* | AT3G23440 | Embryo Sac Development |
| 105 | chrA03 | 18367280 | 18368018 | *UNE16* | AT4G13640 | Double Fertilization |
| 106 | chrA03 | 19707171 | 19709160 | *ABS* | AT5G23260 | ovlue development |
| 107 | chrA03 | 20003117 | 20003614 | *ANK6* | AT5G61230 | Embryo Sac Development |
| 108 | chrA03 | 21626356 | 21627712 | *ATRAB1C* | AT4G17530 | pollen development |
| 109 | chrA03 | 21626356 | 21627712 | *ATRAB1A* | AT5G47200 | pollen development |
| 110 | chrA03 | 22806956 | 22810375 | *ATML1* | AT4G21750 | embryo development ending in seed dormancy |
| 111 | chrA03 | 24667190 | 24671749 | *UNE17* | AT4G26330 | Double Fertilization |
| 112 | chrA03 | 25479037 | 25480454 | *ATMND1* | AT4G29170 | Embryo Sac Development |
| 113 | chrA03 | 25795831 | 25796224 | *BEL1* | AT5G41410 | ovlue development |
| 114 | chrA03 | 26126267 | 26127820 | *ATS2* | AT4G30580 | embryo development ending in seed dormancy |
| 115 | chrA03 | 26458036 | 26460593 | *EDA9* | AT4G34200 | Embryo Sac Development |
| 116 | chrA03 | 26511390 | 26512610 | *NFD1* | AT4G30930 | Embryo Sac Development |
| 117 | chrA03 | 28665636 | 28670353 | *ACA7* | AT2G22950 | pollen development |
| 118 | chrA03 | 28896124 | 28898271 | *EDA40* | AT4G37890 | Embryo Sac Development |
| 119 | chrA03 | 28896365 | 28898190 | *EDA40* | AT4G37890 | Embryo Sac Development |
| 120 | chrA04 | 434080 | 435345 | *RMF* | AT3G61730 | pollen development |
| 121 | chrA04 | 615878 | 617304 | *AGL13* | AT3G61120 | ovlue development |
| 122 | chrA04 | 676944 | 680231 | *BIG3* | AT1G01960 | Embryo Sac Development |
| 123 | chrA04 | 990417 | 992428 | *ATCS-C* | AT3G59760 | Double Fertilization |
| 124 | chrA04 | 2468202 | 2471014 | *OVA1* | AT3G55400 | ovlue development |
| 125 | chrA04 | 4873489 | 4875423 | *EDA38* | AT4G14040 | Embryo Sac Development |
| 126 | chrA04 | 5074434 | 5076303 | *EDA36* | AT4G13890 | Embryo Sac Development |
| 127 | chrA04 | 5075707 | 5076288 | *EDA36* | AT4G13890 | Embryo Sac Development |
| 128 | chrA04 | 5860773 | 5861419 | *ATMGT5* | AT4G28580 | pollen development |
| 129 | chrA04 | 5940521 | 5940972 | *UNE14* | AT4G12860 | Double Fertilization |
| 130 | chrA04 | 6552270 | 6553966 | *SAP* | AT5G35770 | Embryo Sac Development |
| 131 | chrA04 | 8892737 | 8894110 | *EMB139* | AT5G40160 | embryo development ending in seed dormancy |
| 132 | chrA04 | 9540046 | 9542111 | *ATNOB1* | AT5G41190 | Embryo Sac Development |
| 133 | chrA04 | 11075110 | 11075384 | *EC1.3* | AT2G21750 | Double Fertilization |
| 134 | chrA04 | 11075123 | 11075405 | *EC1.2* | AT2G21740 | Double Fertilization |
| 135 | chrA04 | 11589148 | 11590869 | *EDA40* | AT4G37890 | Embryo Sac Development |
| 136 | chrA04 | 12923041 | 12923719 | *AGL61* | AT2G24840 | Embryo Sac Development |
| 137 | chrA04 | 13282658 | 13283280 | *HLL* | AT1G17560 | ovlue development |
| 138 | chrA04 | 16107851 | 16110074 | *WDR55* | AT2G34260 | Embryo Sac Development |
| 139 | chrA04 | 16225392 | 16229497 | *ATHB14* | AT2G34710 | ovlue development |
| 140 | chrA04 | 16225720 | 16229449 | *ATHB9* | AT1G30490 | ovlue development |
| 141 | chrA04 | 16263918 | 16265387 | *EDA28* | AT2G34790 | Embryo Sac Development |
| 142 | chrA04 | 16294869 | 16295531 | *EDA3* | AT2G34860 | Embryo Sac Development |
| 143 | chrA04 | 16589001 | 16591656 | *BLH1* | AT2G35940 | Embryo Sac Development |
| 144 | chrA04 | 17180732 | 17185316 | *ATAMPD* | AT2G38280 | embryo development ending in seed dormancy |
| 145 | chrA04 | 17556534 | 17557803 | *PSF3* | AT1G19080 | ovlue development |
| 146 | chrA04 | 18213104 | 18215362 | *EMB2776* | AT2G41500 | Embryo Sac Development |
| 147 | chrA04 | 18830488 | 18832893 | *ACS1* | AT2G43750 | Double Fertilization |
| 148 | chrA04 | 19402355 | 19403834 | *ARID1* | AT2G46040 | pollen development |
| 149 | chrA05 | 230069 | 232252 | *ATPDI11* | AT2G47470 | Embryo Sac Development |
| 150 | chrA05 | 1251198 | 1252948 | *EMB2776* | AT2G41500 | Embryo Sac Development |
| 151 | chrA05 | 1619872 | 1622639 | *AGL5* | AT2G42830 | ovlue development |
| 152 | chrA05 | 1620139 | 1625081 | *AGL5* | AT2G42830 | ovlue development |
| 153 | chrA05 | 1929742 | 1931665 | *ATCS-C* | AT3G59760 | Double Fertilization |
| 154 | chrA05 | 1929789 | 1932015 | *ACS1* | AT2G43750 | Double Fertilization |
| 155 | chrA05 | 1937140 | 1939060 | *ATMAPK6* | AT2G43790 | ovlue development |
| 156 | chrA05 | 2622486 | 2624304 | *AGL13* | AT3G61120 | ovlue development |
| 157 | chrA05 | 2751653 | 2752928 | *ARID1* | AT2G46040 | pollen development |
| 158 | chrA05 | 2908009 | 2908678 | *PSF3* | AT1G19080 | ovlue development |
| 159 | chrA05 | 3483773 | 3488455 | *ATAMPD* | AT2G38280 | embryo development ending in seed dormancy |
| 160 | chrA05 | 4588134 | 4590819 | *BLH1* | AT2G35940 | Embryo Sac Development |
| 161 | chrA05 | 4649658 | 4662528 | *GEM1* | AT2G35630 | pollen development |
| 162 | chrA05 | 4817465 | 4818319 | *AHL21* | AT2G35270 | ovlue development |
| 163 | chrA05 | 4937866 | 4940617 | *EDA18* | AT2G34920 | Embryo Sac Development |
| 164 | chrA05 | 4955645 | 4956798 | *EDA3* | AT2G34860 | Embryo Sac Development |
| 165 | chrA05 | 4976937 | 4979484 | *EDA28* | AT2G34790 | Embryo Sac Development |
| 166 | chrA05 | 5027057 | 5031439 | *ATHB14* | AT2G34710 | ovlue development |
| 167 | chrA05 | 5027095 | 5031207 | *ATHB9* | AT1G30490 | ovlue development |
| 168 | chrA05 | 5266467 | 5268732 | *WDR55* | AT2G34260 | Embryo Sac Development |
| 169 | chrA05 | 5389268 | 5390803 | *HB-3* | AT2G33880 | embryo development ending in seed dormancy |
| 170 | chrA05 | 6430114 | 6432858 | *FIONA* | AT2G31170 | Embryo Sac Development |
| 171 | chrA05 | 8085051 | 8086604 | *UNE17* | AT4G26330 | Double Fertilization |
| 172 | chrA05 | 10644284 | 10646115 | *RGE1* | AT1G49770 | ovlue development |
| 173 | chrA05 | 10714318 | 10715010 | *EMB1273* | AT1G49510 | ovlue development |
| 174 | chrA05 | 10938471 | 10942178 | *KAN2* | AT1G32240 | ovlue development |
| 175 | chrA05 | 11557471 | 11557669 | *MIR167A* | AT3G22886 | ovlue development |
| 176 | chrA05 | 14904824 | 14906201 | *SGP2* | AT3G21700 | pollen development |
| 177 | chrA05 | 15548973 | 15549975 | *AGC2-3* | AT1G51170 | ovlue development |
| 178 | chrA05 | 15708904 | 15714018 | *ATUBP14* | AT3G20630 | embryo development ending in seed dormancy |
| 179 | chrA05 | 16385441 | 16386804 | *ATMGT5* | AT4G28580 | pollen development |
| 180 | chrA05 | 17980875 | 17981601 | *LEA7* | AT1G52690 | ovlue development |
| 181 | chrA05 | 18086566 | 18087472 | *ANAC018* | AT1G52880 | ovlue development |
| 182 | chrA05 | 19019655 | 19022772 | *OVA5* | AT3G13490 | ovlue development |
| 183 | chrA05 | 19605144 | 19617185 | *RRP5* | AT3G11964 | Embryo Sac Development |
| 184 | chrA05 | 19859078 | 19860781 | *ATMYB65* | AT3G11440 | pollen development |
| 185 | chrA05 | 20503659 | 20506803 | *DEX1* | AT3G09090 | pollen development |
| 186 | chrA05 | 20628491 | 20631267 | *IPGAM2* | AT3G08590 | pollen development |
| 187 | chrA05 | 20628996 | 20631303 | *IPGAM1* | AT1G09780 | pollen development |
| 188 | chrA05 | 21419317 | 21420873 | *ATPPR2* | AT3G06430 | embryo development ending in seed dormancy |
| 189 | chrA05 | 21429771 | 21435085 | *CHR11* | AT3G06400 | Embryo Sac Development |
| 190 | chrA05 | 21868247 | 21870426 | *ATS6A.2* | AT3G05530 | Embryo Sac Development |
| 191 | chrA05 | 22142111 | 22148267 | *CRM1B* | AT3G03110 | Embryo Sac Development |
| 192 | chrA05 | 22422230 | 22423972 | *EDA5* | AT3G03650 | Embryo Sac Development |
| 193 | chrA06 | 175900 | 188521 | *ATDEK1* | AT1G55350 | embryo development ending in seed dormancy |
| 194 | chrA06 | 1052166 | 1053225 | *ANAC018* | AT1G52880 | ovlue development |
| 195 | chrA06 | 1130392 | 1134468 | *ATHB15* | AT1G52150 | ovlue development |
| 196 | chrA06 | 1352549 | 1354560 | *ASH2R* | AT1G51450 | embryo development ending in seed dormancy |
| 197 | chrA06 | 1419763 | 1420857 | *AGC2-3* | AT1G51170 | ovlue development |
| 198 | chrA06 | 1869077 | 1871025 | *RGE1* | AT1G49770 | ovlue development |
| 199 | chrA06 | 2446854 | 2448960 | *ATCUL1* | AT4G02570 | embryo development ending in seed dormancy |
| 200 | chrA06 | 2616656 | 2618010 | *APX1* | AT1G07890 | Embryo Development |
| 201 | chrA06 | 2616680 | 2617770 | *APX1* | AT1G07890 | Embryo Development |
| 202 | chrA06 | 2741453 | 2756354 | *ABO4* | AT1G08260 | Embryo Development |
| 203 | chrA06 | 3326362 | 3328772 | *GRP23* | AT1G10270 | Embryo Development |
| 204 | chrA06 | 3405103 | 3406221 | *ARR4* | AT1G10470 | Embryo Development |
| 205 | chrA06 | 3460039 | 3463293 | *EMB2004* | AT1G10510 | Embryo Development |
| 206 | chrA06 | 3637060 | 3641259 | *ATWAPL1* | AT1G11060 | embryo development ending in seed dormancy |
| 207 | chrA06 | 3637455 | 3641080 | *ATWAPL2* | AT1G61030 | embryo development ending in seed dormancy |
| 208 | chrA06 | 3675571 | 3679605 | *SCM* | AT1G11130 | ovlue development |
| 209 | chrA06 | 3874293 | 3876062 | *ATCYP5* | AT1G11680 | Embryo Development |
| 210 | chrA06 | 4127930 | 4128701 | *DRN* | AT1G12980 | ovlue development |
| 211 | chrA06 | 4433104 | 4434489 | *ANAC007* | AT1G12260 | Embryo Development |
| 212 | chrA06 | 4787852 | 4789861 | *FAC19* | AT1G13800 | ovlue development |
| 213 | chrA06 | 4963916 | 4966165 | *ATMYB124* | AT1G14350 | Embryo Sac Development |
| 214 | chrA06 | 5114305 | 5116894 | *TTA1* | AT1G14740 | ovlue development |
| 215 | chrA06 | 5368716 | 5371972 | *ATPWP2* | AT1G15440 | Embryo Sac Development |
| 216 | chrA06 | 5488857 | 5494085 | *TPL* | AT1G15750 | ovlue development |
| 217 | chrA06 | 6116402 | 6117390 | *HLL* | AT1G17560 | ovlue development |
| 218 | chrA06 | 6478289 | 6482240 | *ATNACK1* | AT1G18370 | Embryo Sac Development |
| 219 | chrA06 | 6650773 | 6652181 | *PSP* | AT1G18640 | ovlue development |
| 220 | chrA06 | 6758619 | 6779546 | *AGL65* | AT1G18750 | pollen development |
| 221 | chrA06 | 7020012 | 7022283 | *DA1* | AT1G19270 | ovlue development |
| 222 | chrA06 | 7210690 | 7211758 | *BZR1* | AT1G75080 | ovlue development |
| 223 | chrA06 | 7541946 | 7545554 | *ARF5* | AT1G19850 | ovlue development |
| 224 | chrA06 | 8290749 | 8291385 | *EMB2170* | AT1G21390 | ovlue development |
| 225 | chrA06 | 8418395 | 8421331 | *SECA2* | AT1G21650 | ovlue development |
| 226 | chrA06 | 8444561 | 8447327 | *EMB1968* | AT1G21690 | ovlue development |
| 227 | chrA06 | 9358011 | 9359921 | *ATBT2* | AT3G48360 | Embryo Sac Development |
| 228 | chrA06 | 9419631 | 9429032 | *ATHDA7* | AT5G35600 | Embryo Sac Development |
| 229 | chrA06 | 9652069 | 9652556 | *ASL29* | AT3G47870 | pollen development |
| 230 | chrA06 | 10591795 | 10593342 | *ATMAPK3* | AT3G45640 | ovlue development |
| 231 | chrA06 | 12082422 | 12086425 | *ATNACK2* | AT3G43210 | Embryo Sac Development |
| 232 | chrA06 | 15781826 | 15783362 | *BT1* | AT5G63160 | Embryo Sac Development |
| 233 | chrA06 | 15782731 | 15783254 | *ATBT2* | AT3G48360 | Embryo Sac Development |
| 234 | chrA06 | 16017834 | 16018791 | *ARI14* | AT5G63730 | Double Fertilization |
| 235 | chrA06 | 16425268 | 16425492 | *EC1.5* | AT5G64720 | Double Fertilization |
| 236 | chrA06 | 17457095 | 17458084 | *ARL2* | AT2G18390 | Embryo Sac Development |
| 237 | chrA06 | 19527758 | 19528886 | *DPD1* | AT5G26940 | pollen development |
| 238 | chrA06 | 19631258 | 19634422 | *EMB161* | AT5G27740 | embryo development ending in seed dormancy |
| 239 | chrA06 | 20243317 | 20250768 | *OVA2* | AT5G49030 | ovlue development |
| 240 | chrA06 | 20366617 | 20368399 | *XRI* | AT5G48720 | pollen development |
| 241 | chrA06 | 20526544 | 20528893 | *AACT2* | AT5G48230 | embryo development ending in seed dormancy |
| 242 | chrA06 | 20569321 | 20572446 | *GFA2* | AT5G48030 | Embryo Sac Development |
| 243 | chrA06 | 22422150 | 22423888 | *ATSPP* | AT2G03120 | pollen development |
| 244 | chrA06 | 22513737 | 22516004 | *ATMYB124* | AT1G14350 | Embryo Sac Development |
| 245 | chrA06 | 23256508 | 23258911 | *ATPRD3* | AT1G01690 | Embryo Sac Development |
| 246 | chrA06 | 23325872 | 23327185 | *ATRAB1A* | AT5G47200 | pollen development |
| 247 | chrA06 | 23729031 | 23729609 | *HLL* | AT1G17560 | ovlue development |
| 248 | chrA07 | 752652 | 753762 | *PLA2-BETA* | AT2G19690 | pollen development |
| 249 | chrA07 | 2120417 | 2123506 | *EMB2762* | AT2G17250 | embryo development ending in seed dormancy |
| 250 | chrA07 | 3623865 | 3625836 | *LIP1* | AT2G15230 | Double Fertilization |
| 251 | chrA07 | 6838333 | 6838633 | *EDA6* | AT3G23440 | Embryo Sac Development |
| 252 | chrA07 | 7133078 | 7134826 | *ENP1* | AT1G31660 | Embryo Sac Development |
| 253 | chrA07 | 7400158 | 7404013 | *EDA28* | AT2G34790 | Embryo Sac Development |
| 254 | chrA07 | 8606940 | 8609102 | *ATLEC2* | AT1G28300 | ovlue development |
| 255 | chrA07 | 8934102 | 8936092 | *SEPALLATA3* | AT1G24260 | ovlue development |
| 256 | chrA07 | 8972077 | 8975002 | *EMB2421* | AT1G24340 | embryo development ending in seed dormancy |
| 257 | chrA07 | 9576382 | 9578016 | *INO* | AT1G23420 | ovlue development |
| 258 | chrA07 | 9678746 | 9681207 | *PIN7* | AT1G23080 | embryo development ending in seed dormancy |
| 259 | chrA07 | 10069559 | 10071302 | *AGL104* | AT1G22130 | pollen development |
| 260 | chrA07 | 10069559 | 10070198 | *AGL66* | AT1G77980 | pollen development |
| 261 | chrA07 | 10129272 | 10131140 | *ATLEC1* | AT1G21970 | embryo development ending in seed dormancy |
| 262 | chrA07 | 10476451 | 10484380 | *EMB1507* | AT1G20960 | ovlue development |
| 263 | chrA07 | 10689511 | 10693108 | *EMB2719* | AT1G20200 | ovlue development |
| 264 | chrA07 | 10780376 | 10784036 | *ARF5* | AT1G19850 | ovlue development |
| 265 | chrA07 | 12973419 | 12975423 | *ATCUL1* | AT4G02570 | embryo development ending in seed dormancy |
| 266 | chrA07 | 13267811 | 13269905 | *ATNOB1* | AT5G41190 | Embryo Sac Development |
| 267 | chrA07 | 14388688 | 14392023 | *EDA7* | AT3G56990 | Embryo Sac Development |
| 268 | chrA07 | 14877693 | 14881213 | *AGL5* | AT2G42830 | ovlue development |
| 269 | chrA07 | 15363277 | 15364642 | *RMF* | AT3G61730 | pollen development |
| 270 | chrA07 | 15778121 | 15783392 | *TPL* | AT1G15750 | ovlue development |
| 271 | chrA07 | 15943226 | 15948055 | *EMB1047* | AT1G79560 | embryo development ending in seed dormancy |
| 272 | chrA07 | 15943762 | 15946507 | *EMB1047* | AT1G79560 | embryo development ending in seed dormancy |
| 273 | chrA07 | 16413095 | 16413501 | *EMB2170* | AT1G21390 | ovlue development |
| 274 | chrA07 | 16510634 | 16511041 | *EC1.1* | AT1G76750 | Double Fertilization |
| 275 | chrA07 | 16876973 | 16878554 | *BZR1* | AT1G75080 | ovlue development |
| 276 | chrA07 | 17335863 | 17339209 | *EDA17* | AT1G72970 | Embryo Sac Development |
| 277 | chrA07 | 17608676 | 17611424 | *ATSERK1* | AT1G71830 | embryo development ending in seed dormancy |
| 278 | chrA07 | 17762877 | 17765594 | *PIN7* | AT1G23080 | embryo development ending in seed dormancy |
| 279 | chrA07 | 18836761 | 18842782 | *GLC* | AT1G65450 | Double Fertilization |
| 280 | chrA07 | 20065702 | 20071609 | *MGP3* | AT1G68990 | embryo development ending in seed dormancy |
| 281 | chrA07 | 20860996 | 20862769 | *PIN7* | AT1G23080 | embryo development ending in seed dormancy |
| 282 | chrA07 | 21145459 | 21148580 | *ATSERK1* | AT1G71830 | embryo development ending in seed dormancy |
| 283 | chrA07 | 21213343 | 21214956 | *HISN6A* | AT5G10330 | embryo development ending in seed dormancy |
| 284 | chrA07 | 21426575 | 21431150 | *EDA25* | AT1G72440 | Embryo Sac Development |
| 285 | chrA07 | 22377500 | 22378603 | *EMB2719* | AT1G20200 | ovlue development |
| 286 | chrA07 | 22947636 | 22954147 | *ASHH2* | AT1G77300 | Embryo Sac Development |
| 287 | chrA07 | 23119859 | 23121926 | *AGL104* | AT1G22130 | pollen development |
| 288 | chrA07 | 23119859 | 23121056 | *AGL66* | AT1G77980 | pollen development |
| 289 | chrA08 | 1082593 | 1084740 | *ATCKS* | AT1G53000 | pollen development |
| 290 | chrA08 | 1181951 | 1182516 | *LEA7* | AT1G52690 | ovlue development |
| 291 | chrA08 | 1331436 | 1335137 | *ATHB15* | AT1G52150 | ovlue development |
| 292 | chrA08 | 1809559 | 1810527 | *YAO* | AT4G05410 | Embryo Sac Development |
| 293 | chrA08 | 2430173 | 2432741 | *EMB1144* | AT1G48850 | ovlue development |
| 294 | chrA08 | 2809287 | 2840682 | *ATTAD2* | AT1G48175 | ovlue development |
| 295 | chrA08 | 3715093 | 3720041 | *ATMCM2* | AT1G44900 | ovlue development |
| 296 | chrA08 | 5089129 | 5090603 | *UNE16* | AT4G13640 | Double Fertilization |
| 297 | chrA08 | 6058773 | 6059289 | *EDA36* | AT4G13890 | Embryo Sac Development |
| 298 | chrA08 | 6227230 | 6228098 | *RPL21C* | AT1G35680 | ovlue development |
| 299 | chrA08 | 6345253 | 6347837 | *EMB2756* | AT1G34550 | ovlue development |
| 300 | chrA08 | 6985395 | 6987521 | *ATP8* | AT1G32230 | ovlue development |
| 301 | chrA08 | 7544646 | 7546739 | *EDA38* | AT4G14040 | Embryo Sac Development |
| 302 | chrA08 | 8198226 | 8199601 | *ATRAB1C* | AT4G17530 | pollen development |
| 303 | chrA08 | 9394311 | 9398093 | *ATML1* | AT4G21750 | embryo development ending in seed dormancy |
| 304 | chrA08 | 9688860 | 9696324 | *EMB1507* | AT1G20960 | ovlue development |
| 305 | chrA08 | 10296526 | 10298379 | *EDA9* | AT4G34200 | Embryo Sac Development |
| 306 | chrA08 | 11301885 | 11303188 | *NFD1* | AT4G30930 | Embryo Sac Development |
| 307 | chrA08 | 13059678 | 13067722 | *ACA7* | AT2G22950 | pollen development |
| 308 | chrA08 | 13736249 | 13737693 | *RHF1A* | AT4G14220 | Embryo Sac Development |
| 309 | chrA08 | 15509684 | 15512194 | *PIN7* | AT1G23080 | embryo development ending in seed dormancy |
| 310 | chrA08 | 15628487 | 15630471 | *AGL104* | AT1G22130 | pollen development |
| 311 | chrA08 | 15628487 | 15629923 | *AGL66* | AT1G77980 | pollen development |
| 312 | chrA08 | 15698061 | 15698586 | *ATLEC1* | AT1G21970 | embryo development ending in seed dormancy |
| 313 | chrA08 | 15775786 | 15778484 | *EMB1968* | AT1G21690 | ovlue development |
| 314 | chrA08 | 15781476 | 15788209 | *SECA2* | AT1G21650 | ovlue development |
| 315 | chrA08 | 16043265 | 16044172 | *EMB2719* | AT1G20200 | ovlue development |
| 316 | chrA08 | 16162602 | 16163694 | *BZR1* | AT1G75080 | ovlue development |
| 317 | chrA08 | 16184128 | 16186030 | *DA1* | AT1G19270 | ovlue development |
| 318 | chrA08 | 16305270 | 16311262 | *AGL65* | AT1G18750 | pollen development |
| 319 | chrA08 | 16341881 | 16343452 | *PSP* | AT1G18640 | ovlue development |
| 320 | chrA08 | 16830784 | 16833923 | *ATPWP2* | AT1G15440 | Embryo Sac Development |
| 321 | chrA08 | 17154631 | 17156263 | *EMB1586* | AT1G12770 | Embryo Development |
| 322 | chrA08 | 17253545 | 17254899 | *ANAC007* | AT1G12260 | Embryo Development |
| 323 | chrA08 | 17349225 | 17351865 | *OVA7* | AT1G11870 | ovlue development |
| 324 | chrA08 | 17576420 | 17578761 | *ACLA-1* | AT1G10670 | Embryo Development |
| 325 | chrA08 | 17621341 | 17624360 | *EMB2004* | AT1G10510 | Embryo Development |
| 326 | chrA08 | 17651941 | 17653282 | *ARR4* | AT1G10470 | Embryo Development |
| 327 | chrA08 | 17691898 | 17694291 | *GRP23* | AT1G10270 | Embryo Development |
| 328 | chrA08 | 17797107 | 17799624 | *IPGAM1* | AT1G09780 | pollen development |
| 329 | chrA08 | 17797807 | 17799593 | *IPGAM2* | AT3G08590 | pollen development |
| 330 | chrA08 | 18006799 | 18010397 | *PIAL1* | AT1G08910 | Embryo Development |
| 331 | chrA08 | 18069363 | 18072425 | *ATPXL1* | AT1G08590 | Embryo Development |
| 332 | chrA08 | 18134121 | 18136950 | *DIG6* | AT1G08410 | Embryo Development |
| 333 | chrA08 | 18211348 | 18212169 | *TAF13* | AT1G02680 | Embryo Development |
| 334 | chrA08 | 18214422 | 18215458 | *EMB2386* | AT1G02780 | Embryo Development |
| 335 | chrA08 | 18480518 | 18481544 | *MEE4* | AT1G04630 | Embryo Development |
| 336 | chrA08 | 18615516 | 18619987 | *CVP2* | AT1G05470 | Embryo Development |
| 337 | chrA08 | 18676858 | 18678014 | *BT3* | AT1G05690 | Embryo Sac Development |
| 338 | chrA08 | 18725073 | 18728912 | *CLO* | AT1G06220 | Embryo Sac Development |
| 339 | chrA09 | 379205 | 380154 | *EMB2386* | AT1G02780 | Embryo Development |
| 340 | chrA09 | 533272 | 537159 | *ATCUL1* | AT4G02570 | embryo development ending in seed dormancy |
| 341 | chrA09 | 540645 | 542578 | *UNE12* | AT4G02590 | Double Fertilization |
| 342 | chrA09 | 921722 | 922692 | *EDA35* | AT4G05440 | Embryo Sac Development |
| 343 | chrA09 | 1448016 | 1450286 | *OBE2* | AT5G48160 | embryo development ending in seed dormancy |
| 344 | chrA09 | 1460105 | 1462425 | *AACT2* | AT5G48230 | embryo development ending in seed dormancy |
| 345 | chrA09 | 1584365 | 1585692 | *XRI* | AT5G48720 | pollen development |
| 346 | chrA09 | 2642207 | 2644181 | *ABS* | AT5G23260 | ovlue development |
| 347 | chrA09 | 2766980 | 2767478 | *ANK6* | AT5G61230 | Embryo Sac Development |
| 348 | chrA09 | 2814879 | 2817087 | *SAP130A* | AT3G55200 | pollen development |
| 349 | chrA09 | 3393124 | 3393320 | *EC1.5* | AT5G64720 | Double Fertilization |
| 350 | chrA09 | 3906809 | 3908459 | *AKRP* | AT5G66055 | embryo development ending in seed dormancy |
| 351 | chrA09 | 4487912 | 4490816 | *EMB2762* | AT2G17250 | embryo development ending in seed dormancy |
| 352 | chrA09 | 4737790 | 4740031 | *EDA2* | AT2G18080 | Embryo Sac Development |
| 353 | chrA09 | 5055402 | 5055800 | *PLA2-BETA* | AT2G19690 | pollen development |
| 354 | chrA09 | 5568806 | 5570713 | *UGE3* | AT1G63180 | pollen development |
| 355 | chrA09 | 5858928 | 5860844 | *SPO11-2SPO11-2* | AT1G63990 | Embryo Sac Development |
| 356 | chrA09 | 6505902 | 6509412 | *EMB71* | AT1G63700 | embryo development ending in seed dormancy |
| 357 | chrA09 | 8219192 | 8220115 | *ARR4* | AT1G10470 | Embryo Development |
| 358 | chrA09 | 9516063 | 9523237 | *SPP* | AT5G42390 | embryo development ending in seed dormancy |
| 359 | chrA09 | 10847703 | 10849085 | *MOB1-LIKE* | AT5G45550 | Embryo Sac Development |
| 360 | chrA09 | 10996949 | 10997768 | *HLL* | AT1G17560 | ovlue development |
| 361 | chrA09 | 11586967 | 11588142 | *HOS9* | AT2G01500 | Embryo Sac Development |
| 362 | chrA09 | 12734575 | 12735264 | *YAO* | AT4G05410 | Embryo Sac Development |
| 363 | chrA09 | 13534078 | 13536252 | *UNE13* | AT4G12620 | Double Fertilization |
| 364 | chrA09 | 14153605 | 14166063 | *GCS1* | AT4G11720 | Double Fertilization |
| 365 | chrA09 | 17176366 | 17180160 | *KAN2* | AT1G32240 | ovlue development |
| 366 | chrA09 | 18194146 | 18195826 | *ENP1* | AT1G31660 | Embryo Sac Development |
| 367 | chrA09 | 18573979 | 18576456 | *EDA18* | AT2G34920 | Embryo Sac Development |
| 368 | chrA09 | 18834950 | 18837150 | *EDA28* | AT2G34790 | Embryo Sac Development |
| 369 | chrA09 | 19081363 | 19084688 | *EMB2279* | AT1G30610 | embryo development ending in seed dormancy |
| 370 | chrA09 | 20528487 | 20530609 | *ATLEC2* | AT1G28300 | ovlue development |
| 371 | chrA09 | 20976037 | 20978177 | *SEPALLATA3* | AT1G24260 | ovlue development |
| 372 | chrA09 | 21341433 | 21346338 | *OVA9* | AT1G25350 | ovlue development |
| 373 | chrA09 | 23076921 | 23078633 | *INO* | AT1G23420 | ovlue development |
| 374 | chrA09 | 23231391 | 23233092 | *TAR1* | AT1G23320 | ovlue development |
| 375 | chrA09 | 25258637 | 25261502 | *FBL17* | AT3G54650 | pollen development |
| 376 | chrA09 | 25553115 | 25556169 | *OVA1* | AT3G55400 | ovlue development |
| 377 | chrA09 | 26324688 | 26328567 | *EDA7* | AT3G56990 | Embryo Sac Development |
| 378 | chrA09 | 26751868 | 26752264 | *GIG1* | AT3G57860 | Embryo Sac Development |
| 379 | chrA09 | 27198522 | 27201724 | *ATSYN3* | AT3G59550 | pollen development |
| 380 | chrA09 | 27453803 | 27455190 | *EDA14* | AT3G60360 | Embryo Sac Development |
| 381 | chrA09 | 28828430 | 28829152 | *AGL61* | AT2G24840 | Embryo Sac Development |
| 382 | chrA09 | 29022862 | 29025212 | *LAP1* | AT2G24200 | Double Fertilization |
| 383 | chrA09 | 29373681 | 29378395 | *ACA7* | AT2G22950 | pollen development |
| 384 | chrA09 | 29865954 | 29866303 | *EC1.2* | AT2G21740 | Double Fertilization |
| 385 | chrA09 | 29865954 | 29866288 | *EC1.3* | AT2G21750 | Double Fertilization |
| 386 | chrA09 | 30634193 | 30638001 | *ATNACK1* | AT1G18370 | Embryo Sac Development |
| 387 | chrA09 | 31039277 | 31042554 | *ADL1C* | AT1G14830 | pollen development |
| 388 | chrA09 | 31609597 | 31612698 | *ATGLE1* | AT1G13120 | embryo development ending in seed dormancy |
| 389 | chrA09 | 31659546 | 31660213 | *DRN* | AT1G12980 | ovlue development |
| 390 | chrA09 | 31868950 | 31870311 | *ANAC007* | AT1G12260 | Embryo Development |
| 391 | chrA09 | 32001104 | 32003370 | *OVA7* | AT1G11870 | ovlue development |
| 392 | chrA09 | 32126086 | 32129855 | *SCM* | AT1G11130 | ovlue development |
| 393 | chrA09 | 32216411 | 32220660 | *ATWAPL1* | AT1G11060 | embryo development ending in seed dormancy |
| 394 | chrA09 | 32217333 | 32220462 | *ATWAPL2* | AT1G61030 | embryo development ending in seed dormancy |
| 395 | chrA09 | 32313289 | 32314486 | *ARR4* | AT1G10470 | Embryo Development |
| 396 | chrA09 | 32359388 | 32361548 | *GRP23* | AT1G10270 | Embryo Development |
| 397 | chrA09 | 32550518 | 32553532 | *PIAL1* | AT1G08910 | Embryo Development |
| 398 | chrA09 | 32565525 | 32574408 | *EMB2411* | AT1G08840 | Embryo Development |
| 399 | chrA09 | 32785666 | 32787331 | *APX1* | AT1G07890 | Embryo Development |
| 400 | chrA09 | 32785918 | 32787331 | *APX1* | AT1G07890 | Embryo Development |
| 401 | chrA09 | 33295768 | 33297209 | *CVP2* | AT1G05470 | Embryo Development |
| 402 | chrA09 | 33342861 | 33344082 | *EMB2394* | AT1G05190 | Embryo Development |
| 403 | chrA09 | 33508141 | 33511073 | *CACTIN* | AT1G03910 | Embryo Development |
| 404 | chrA09 | 33508141 | 33509902 | *CACTIN* | AT1G03910 | Embryo Development |
| 405 | chrA09 | 33695370 | 33696447 | *EMB2386* | AT1G02780 | Embryo Development |
| 406 | chrA10 | 32705 | 33523 | *HAP1* | AT1G02140 | Embryo Development |
| 407 | chrA10 | 50457 | 52063 | *SPL8* | AT1G02065 | Embryo Sac Development |
| 408 | chrA10 | 159915 | 161793 | *ATPRD3* | AT1G01690 | Embryo Sac Development |
| 409 | chrA10 | 213761 | 214813 | *BPS1* | AT1G01550 | Embryo Development |
| 410 | chrA10 | 298987 | 300660 | *CYP703* | AT1G01280 | pollen development |
| 411 | chrA10 | 390571 | 397572 | *ASU1* | AT1G01040 | Embryo Development |
| 412 | chrA10 | 708253 | 709086 | *TAF13* | AT1G02680 | Embryo Development |
| 413 | chrA10 | 735776 | 737063 | *EMB2386* | AT1G02780 | Embryo Development |
| 414 | chrA10 | 749577 | 749805 | *ATLEA3* | AT1G02820 | Embryo Development |
| 415 | chrA10 | 1177533 | 1181400 | *CACTIN* | AT1G03910 | Embryo Development |
| 416 | chrA10 | 1412801 | 1413845 | *BDL* | AT1G04550 | Embryo Development |
| 417 | chrA10 | 1452232 | 1453386 | *MEE4* | AT1G04630 | Embryo Development |
| 418 | chrA10 | 1453710 | 1454195 | *ATPOP5* | AT1G04635 | Embryo Development |
| 419 | chrA10 | 1633979 | 1635135 | *EMB2394* | AT1G05190 | Embryo Development |
| 420 | chrA10 | 1790795 | 1792201 | *BT3* | AT1G05690 | Embryo Sac Development |
| 421 | chrA10 | 1927669 | 1931451 | *CVP2* | AT1G05470 | Embryo Development |
| 422 | chrA10 | 1970048 | 1971005 | *OBAP1A* | AT1G05510 | Embryo Development |
| 423 | chrA10 | 5542602 | 5545101 | *EMB2737* | AT5G53860 | embryo development ending in seed dormancy |
| 424 | chrA10 | 5618465 | 5619913 | *ANAC098* | AT5G53950 | embryo development ending in seed dormancy |
| 425 | chrA10 | 7837097 | 7839805 | *SEU* | AT1G43850 | ovlue development |
| 426 | chrA10 | 8117650 | 8119288 | *ATGPT1* | AT5G54800 | Embryo Sac Development |
| 427 | chrA10 | 8951788 | 8954048 | *ATWRKY2* | AT5G56270 | embryo development ending in seed dormancy |
| 428 | chrA10 | 9932904 | 9933286 | *EDA21* | AT4G13235 | Embryo Sac Development |
| 429 | chrA10 | 11273046 | 11275748 | *MS1* | AT5G22260 | pollen development |
| 430 | chrA10 | 11364173 | 11366074 | *RHF2A* | AT5G22000 | Embryo Sac Development |
| 431 | chrA10 | 11481495 | 11485833 | *AGO9* | AT5G21150 | Embryo Sac Development |
| 432 | chrA10 | 12023049 | 12023442 | *BEL1* | AT5G41410 | ovlue development |
| 433 | chrA10 | 12034877 | 12040876 | *ATOEP80* | AT5G19620 | embryo development ending in seed dormancy |
| 434 | chrA10 | 12448593 | 12453077 | *CHR11* | AT3G06400 | Embryo Sac Development |
| 435 | chrA10 | 12839833 | 12843114 | *UNE6* | AT3G03340 | Double Fertilization |
| 436 | chrA10 | 12950254 | 12956333 | *CRM1B* | AT3G03110 | Embryo Sac Development |
| 437 | chrA10 | 13003563 | 13007393 | *DOT2* | AT5G16780 | embryo development ending in seed dormancy |
| 438 | chrA10 | 13003563 | 13007393 | *DOT2* | AT5G16780 | embryo development ending in seed dormancy |
| 439 | chrA10 | 13015611 | 13020089 | *TOZ* | AT5G16750 | embryo development ending in seed dormancy |
| 440 | chrA10 | 13130963 | 13135751 | *KAN* | AT5G16560 | ovlue development |
| 441 | chrA10 | 13382712 | 13384577 | *AGL2* | AT5G15800 | ovlue development |
| 442 | chrA10 | 13643080 | 13644280 | *PIN8* | AT5G15100 | pollen development |
| 443 | chrA10 | 14005249 | 14009438 | *NEF1* | AT5G13390 | pollen development |
| 444 | chrA10 | 16250585 | 16254596 | *MAA3* | AT4G15570 | Embryo Sac Development |
| 445 | chrA10 | 16428225 | 16429403 | *CYP77A7* | AT3G10560 | Double Fertilization |
| 446 | chrA10 | 16840172 | 16842224 | *EDA31* | AT3G10000 | Embryo Sac Development |
| 447 | chrC01 | 84868 | 85247 | *EC1.4* | AT4G39340 | Double Fertilization |
| 448 | chrC01 | 2187176 | 2189761 | *EDA9* | AT4G34200 | Embryo Sac Development |
| 449 | chrC01 | 3932134 | 3933484 | *NFD1* | AT4G30930 | Embryo Sac Development |
| 450 | chrC01 | 4239708 | 4243874 | *UNE13* | AT4G12620 | Double Fertilization |
| 451 | chrC01 | 4516852 | 4520169 | *EMB2411* | AT1G08840 | Embryo Development |
| 452 | chrC01 | 4722624 | 4725032 | *ALT2* | AT4G29860 | ovlue development |
| 453 | chrC01 | 5952785 | 5954331 | *ATRAB1C* | AT4G17530 | pollen development |
| 454 | chrC01 | 6962037 | 6963804 | *ATMYB98* | AT4G18770 | Embryo Sac Development |
| 455 | chrC01 | 7096023 | 7102254 | *AG* | AT4G18960 | Ovule Development |
| 456 | chrC01 | 11127930 | 11145105 | *EMB140* | AT4G24270 | embryo development ending in seed dormancy |
| 457 | chrC01 | 11458903 | 11460350 | *TTL* | AT4G24900 | embryo development ending in seed dormancy |
| 458 | chrC01 | 11544381 | 11544697 | *TPD1* | AT4G24972 | pollen development |
| 459 | chrC01 | 15815679 | 15820219 | *MAA3* | AT4G15570 | Embryo Sac Development |
| 460 | chrC01 | 16293337 | 16296314 | *EDA15* | AT4G14790 | Embryo Sac Development |
| 461 | chrC01 | 18582710 | 18583050 | *GIG1* | AT3G57860 | Embryo Sac Development |
| 462 | chrC01 | 27250126 | 27255041 | *EDA16* | AT1G61140 | Embryo Sac Development |
| 463 | chrC01 | 27925573 | 27925966 | *BEL1* | AT5G41410 | ovlue development |
| 464 | chrC01 | 28760710 | 28763387 | *UNE16* | AT4G13640 | Double Fertilization |
| 465 | chrC01 | 30215037 | 30215546 | *MIR167A* | AT3G22886 | ovlue development |
| 466 | chrC01 | 35228420 | 35229086 | *LEA7* | AT1G52690 | ovlue development |
| 467 | chrC01 | 37061455 | 37064595 | *ABCG26* | AT3G13220 | pollen development |
| 468 | chrC01 | 37928047 | 37932301 | *DEX1* | AT3G09090 | Double Fertilization |
| 469 | chrC01 | 37928097 | 37932264 | *DEX1* | AT3G09090 | pollen development |
| 470 | chrC01 | 38263675 | 38265412 | *OBE2* | AT5G48160 | embryo development ending in seed dormancy |
| 471 | chrC01 | 38722315 | 38725516 | *EDA30* | AT3G03810 | Embryo Sac Development |
| 472 | chrC01 | 38772100 | 38774022 | *UNE7* | AT3G03690 | Double Fertilization |
| 473 | chrC02 | 925467 | 928329 | *ATO* | AT5G06160 | Embryo Sac Development |
| 474 | chrC02 | 1008933 | 1021354 | *APC1* | AT5G05560 | Embryo Sac Development |
| 475 | chrC02 | 1329803 | 1332395 | *EMB166* | AT5G03800 | embryo development ending in seed dormancy |
| 476 | chrC02 | 1377586 | 1380135 | *EDA31* | AT3G10000 | Embryo Sac Development |
| 477 | chrC02 | 1720931 | 1722241 | *ATASP38* | AT5G02190 | embryo development ending in seed dormancy |
| 478 | chrC02 | 2336920 | 2341898 | *CUV* | AT5G13010 | embryo development ending in seed dormancy |
| 479 | chrC02 | 2488690 | 2492676 | *NEF1* | AT5G13390 | pollen development |
| 480 | chrC02 | 2526726 | 2530712 | *FY* | AT5G13480 | embryo development ending in seed dormancy |
| 481 | chrC02 | 3553943 | 3560937 | *KAN* | AT5G16560 | ovlue development |
| 482 | chrC02 | 3665989 | 3670637 | *TOZ* | AT5G16750 | embryo development ending in seed dormancy |
| 483 | chrC02 | 3718449 | 3725438 | *CRM1B* | AT3G03110 | Embryo Sac Development |
| 484 | chrC02 | 4216282 | 4217244 | *VDD* | AT5G18000 | Embryo Sac Development |
| 485 | chrC02 | 4449293 | 4452432 | *EMB269* | AT5G18570 | embryo development ending in seed dormancy |
| 486 | chrC02 | 4493504 | 4498560 | *CHR11* | AT3G06400 | Embryo Sac Development |
| 487 | chrC02 | 6275166 | 6277143 | *ATMSI1* | AT5G58230 | embryo development ending in seed dormancy |
| 488 | chrC02 | 7761449 | 7762077 | *EMB2394* | AT1G05190 | Embryo Development |
| 489 | chrC02 | 8750455 | 8752229 | *ATGPT1* | AT5G54800 | Embryo Sac Development |
| 490 | chrC02 | 9015761 | 9023793 | *GLC* | AT1G65450 | Double Fertilization |
| 491 | chrC02 | 9218965 | 9220604 | *ANAC098* | AT5G53950 | embryo development ending in seed dormancy |
| 492 | chrC02 | 10461229 | 10463761 | *ATNLE* | AT5G52820 | Embryo Sac Development |
| 493 | chrC02 | 10529122 | 10531844 | *OVA6* | AT5G52520 | ovlue development |
| 494 | chrC02 | 12684986 | 12730879 | *ATMDN1* | AT1G67120 | Embryo Sac Development |
| 495 | chrC02 | 16307862 | 16308263 | *EDA24* | AT1G70540 | Embryo Sac Development |
| 496 | chrC02 | 16880684 | 16883444 | *PIN7* | AT1G23080 | embryo development ending in seed dormancy |
| 497 | chrC02 | 17498469 | 17500311 | *HISN6A* | AT5G10330 | embryo development ending in seed dormancy |
| 498 | chrC02 | 18248866 | 18249803 | *EDA25* | AT1G72440 | Embryo Sac Development |
| 499 | chrC02 | 18609955 | 18612807 | *EDA17* | AT1G72970 | Embryo Sac Development |
| 500 | chrC02 | 21522152 | 21522525 | *EC1.1* | AT1G76750 | Double Fertilization |
| 501 | chrC02 | 23082625 | 23085392 | *EMB1507* | AT1G20960 | ovlue development |
| 502 | chrC02 | 24987805 | 24989566 | *UNE12* | AT4G02590 | Double Fertilization |
| 503 | chrC02 | 26311348 | 26313891 | *EMB2386* | AT1G02780 | Embryo Development |
| 504 | chrC02 | 30345476 | 30346607 | *ATS* | AT5G42630 | ovlue development |
| 505 | chrC02 | 33260286 | 33264023 | *EDA23* | AT5G44700 | Embryo Sac Development |
| 506 | chrC02 | 35728733 | 35730361 | *ATRAB1A* | AT5G47200 | pollen development |
| 507 | chrC02 | 37419373 | 37421189 | *ATMYB124* | AT1G14350 | Embryo Sac Development |
| 508 | chrC02 | 41313412 | 41316510 | *GFA2* | AT5G48030 | Embryo Sac Development |
| 509 | chrC02 | 41445208 | 41453124 | *AACT2* | AT5G48230 | embryo development ending in seed dormancy |
| 510 | chrC02 | 41859856 | 41867103 | *OVA2* | AT5G49030 | ovlue development |
| 511 | chrC02 | 42749923 | 42752577 | *EMB161* | AT5G27740 | embryo development ending in seed dormancy |
| 512 | chrC02 | 44915786 | 44917790 | *ABS* | AT5G23260 | ovlue development |
| 513 | chrC02 | 45058900 | 45061132 | *BT1* | AT5G63160 | Embryo Sac Development |
| 514 | chrC02 | 45059419 | 45060847 | *ATBT2* | AT3G48360 | Embryo Sac Development |
| 515 | chrC03 | 86950 | 91825 | *EDA20* | AT4G00020 | Embryo Sac Development |
| 516 | chrC03 | 1021563 | 1032735 | *APC1* | AT5G05560 | Embryo Sac Development |
| 517 | chrC03 | 1159651 | 1161194 | *EDA13* | AT2G47990 | Embryo Sac Development |
| 518 | chrC03 | 2863436 | 2867660 | *FY* | AT5G13480 | embryo development ending in seed dormancy |
| 519 | chrC03 | 3485743 | 3487895 | *AGL2* | AT5G15800 | ovlue development |
| 520 | chrC03 | 3685932 | 3689356 | *KAN* | AT5G16560 | ovlue development |
| 521 | chrC03 | 3829388 | 3860339 | *CRM1B* | AT3G03110 | Embryo Sac Development |
| 522 | chrC03 | 4001776 | 4002371 | *UNE6* | AT3G03340 | Double Fertilization |
| 523 | chrC03 | 5084874 | 5086493 | *RHF2A* | AT5G22000 | Embryo Sac Development |
| 524 | chrC03 | 6033624 | 6035341 | *ATMSI1* | AT5G58230 | embryo development ending in seed dormancy |
| 525 | chrC03 | 6621322 | 6623368 | *ATWRKY2* | AT5G56270 | embryo development ending in seed dormancy |
| 526 | chrC03 | 6736744 | 6743320 | *WYR* | AT5G55820 | Embryo Sac Development |
| 527 | chrC03 | 9497744 | 9498720 | *MEE4* | AT1G04630 | Embryo Development |
| 528 | chrC03 | 9513136 | 9514278 | *BDL* | AT1G04550 | Embryo Development |
| 529 | chrC03 | 9676730 | 9678215 | *HB-3* | AT2G33880 | embryo development ending in seed dormancy |
| 530 | chrC03 | 10054972 | 10055793 | *AHL21* | AT2G35270 | ovlue development |
| 531 | chrC03 | 10141280 | 10153951 | *GEM1* | AT2G35630 | pollen development |
| 532 | chrC03 | 10358726 | 10361673 | *BLH1* | AT2G35940 | Embryo Sac Development |
| 533 | chrC03 | 11355202 | 11356521 | *APD1* | AT2G38185 | pollen development |
| 534 | chrC03 | 11355691 | 11356240 | *APD3* | AT2G38220 | pollen development |
| 535 | chrC03 | 13194114 | 13199823 | *EMB1507* | AT1G20960 | ovlue development |
| 536 | chrC03 | 13440514 | 13442934 | *NPG1* | AT2G43040 | pollen development |
| 537 | chrC03 | 13735500 | 13737663 | *ATMAPK6* | AT2G43790 | ovlue development |
| 538 | chrC03 | 14736923 | 14739205 | *ATPDI11* | AT2G47470 | Embryo Sac Development |
| 539 | chrC03 | 15534972 | 15537104 | *UNE10* | AT4G00050 | Double Fertilization |
| 540 | chrC03 | 15555676 | 15556218 | *UNE11* | AT4G00080 | Double Fertilization |
| 541 | chrC03 | 16154131 | 16155870 | *CEP1* | AT5G50260 | pollen development |
| 542 | chrC03 | 18241236 | 18242783 | *YAO* | AT4G05410 | Embryo Sac Development |
| 543 | chrC03 | 18780355 | 18782301 | *UNE12* | AT4G02590 | Double Fertilization |
| 544 | chrC03 | 18794188 | 18798198 | *ATCUL1* | AT4G02570 | embryo development ending in seed dormancy |
| 545 | chrC03 | 18958990 | 18959866 | *EMB2386* | AT1G02780 | Embryo Development |
| 546 | chrC03 | 19802879 | 19803511 | *EDA33* | AT4G00120 | Embryo Sac Development |
| 547 | chrC03 | 19819199 | 19821330 | *UNE10* | AT4G00050 | Double Fertilization |
| 548 | chrC03 | 20433215 | 20436789 | *EDA30* | AT3G03810 | Embryo Sac Development |
| 549 | chrC03 | 20821915 | 20824112 | *ATS6A.2* | AT3G05530 | Embryo Sac Development |
| 550 | chrC03 | 20822317 | 20824112 | *RPT5B* | AT1G09100 | Embryo Sac Development |
| 551 | chrC03 | 24204920 | 24205514 | *LEA7* | AT1G52690 | ovlue development |
| 552 | chrC03 | 26587500 | 26592690 | *ATUBP14* | AT3G20630 | embryo development ending in seed dormancy |
| 553 | chrC03 | 27048803 | 27050041 | *SGP2* | AT3G21700 | pollen development |
| 554 | chrC03 | 28225423 | 28225782 | *EDA6* | AT3G23440 | Embryo Sac Development |
| 555 | chrC03 | 28448297 | 28449768 | *UNE16* | AT4G13640 | Double Fertilization |
| 556 | chrC03 | 33184590 | 33185803 | *ARR4* | AT1G10470 | Embryo Development |
| 557 | chrC03 | 33306724 | 33307719 | *ARL2* | AT2G18390 | Embryo Sac Development |
| 558 | chrC03 | 33306724 | 33307212 | *ARL2* | AT2G18390 | Embryo Sac Development |
| 559 | chrC03 | 34456616 | 34456848 | *EC1.5* | AT5G64720 | Double Fertilization |
| 560 | chrC03 | 35070724 | 35074263 | *ARI14* | AT5G63730 | Double Fertilization |
| 561 | chrC03 | 35607432 | 35609064 | *BT1* | AT5G63160 | Embryo Sac Development |
| 562 | chrC03 | 44389463 | 44390989 | *ATMAPK3* | AT3G45640 | ovlue development |
| 563 | chrC03 | 46857967 | 46860058 | *SEPALLATA3* | AT1G24260 | ovlue development |
| 564 | chrC03 | 52140318 | 52142271 | *ATMYB98* | AT4G18770 | Embryo Sac Development |
| 565 | chrC03 | 53907918 | 53911926 | *ATML1* | AT4G21750 | embryo development ending in seed dormancy |
| 566 | chrC03 | 57470158 | 57471455 | *NFD1* | AT4G30930 | Embryo Sac Development |
| 567 | chrC03 | 58725883 | 58728991 | *ATHB15* | AT1G52150 | ovlue development |
| 568 | chrC03 | 58765484 | 58767606 | *ATCKS* | AT1G53000 | pollen development |
| 569 | chrC03 | 60124647 | 60129962 | *ATDEK1* | AT1G55350 | embryo development ending in seed dormancy |
| 570 | chrC04 | 1490047 | 1491803 | *EMB2776* | AT2G41500 | Embryo Sac Development |
| 571 | chrC04 | 2240382 | 2242577 | *ACS1* | AT2G43750 | Double Fertilization |
| 572 | chrC04 | 2240416 | 2242236 | *ATCS-C* | AT3G59760 | Double Fertilization |
| 573 | chrC04 | 2249100 | 2250992 | *ATMAPK6* | AT2G43790 | ovlue development |
| 574 | chrC04 | 3450748 | 3452119 | *ARID1* | AT2G46040 | pollen development |
| 575 | chrC04 | 3742759 | 3743637 | *PSF3* | AT1G19080 | ovlue development |
| 576 | chrC04 | 5246900 | 5251508 | *ATAMPD* | AT2G38280 | embryo development ending in seed dormancy |
| 577 | chrC04 | 5284510 | 5312896 | *APD3* | AT2G38220 | pollen development |
| 578 | chrC04 | 7068891 | 7071698 | *BLH1* | AT2G35940 | Embryo Sac Development |
| 579 | chrC04 | 7232916 | 7244696 | *GEM1* | AT2G35630 | pollen development |
| 580 | chrC04 | 7432088 | 7432930 | *AHL21* | AT2G35270 | ovlue development |
| 581 | chrC04 | 7953872 | 7956345 | *EDA18* | AT2G34920 | Embryo Sac Development |
| 582 | chrC04 | 8022685 | 8025247 | *EDA28* | AT2G34790 | Embryo Sac Development |
| 583 | chrC04 | 8071826 | 8076223 | *ATHB14* | AT2G34710 | ovlue development |
| 584 | chrC04 | 8072536 | 8075994 | *ATHB9* | AT1G30490 | ovlue development |
| 585 | chrC04 | 8702187 | 8703064 | *BDL* | AT1G04550 | Embryo Development |
| 586 | chrC04 | 10860196 | 10862553 | *FIONA* | AT2G31170 | Embryo Sac Development |
| 587 | chrC04 | 22670774 | 22672012 | *RMF* | AT3G61730 | pollen development |
| 588 | chrC04 | 23096413 | 23097626 | *AGL13* | AT3G61120 | ovlue development |
| 589 | chrC04 | 23734791 | 23736698 | *ATCS-C* | AT3G59760 | Double Fertilization |
| 590 | chrC04 | 24054316 | 24057947 | *ATSYN3* | AT3G59550 | pollen development |
| 591 | chrC04 | 24468738 | 24473914 | *AGL5* | AT2G42830 | ovlue development |
| 592 | chrC04 | 26652134 | 26660414 | *OVA1* | AT3G55400 | ovlue development |
| 593 | chrC04 | 30416668 | 30418585 | *EDA38* | AT4G14040 | Embryo Sac Development |
| 594 | chrC04 | 30892529 | 30893129 | *ATMGT5* | AT4G28580 | pollen development |
| 595 | chrC04 | 30970853 | 30971418 | *UNE14* | AT4G12860 | Double Fertilization |
| 596 | chrC04 | 31483064 | 31488259 | *SAP* | AT5G35770 | Embryo Sac Development |
| 597 | chrC04 | 34581093 | 34582034 | *EDA3* | AT2G34860 | Embryo Sac Development |
| 598 | chrC04 | 34734145 | 34736217 | *ATNOB1* | AT5G41190 | Embryo Sac Development |
| 599 | chrC04 | 36630125 | 36631269 | *EDA40* | AT4G37890 | Embryo Sac Development |
| 600 | chrC04 | 37724833 | 37725226 | *BEL1* | AT5G41410 | ovlue development |
| 601 | chrC04 | 38808311 | 38808887 | *AGL61* | AT2G24840 | Embryo Sac Development |
| 602 | chrC04 | 39490438 | 39491396 | *HLL* | AT1G17560 | ovlue development |
| 603 | chrC04 | 43208332 | 43210630 | *CVP2* | AT1G05470 | Embryo Development |
| 604 | chrC04 | 44809001 | 44811632 | *BLH1* | AT2G35940 | Embryo Sac Development |
| 605 | chrC04 | 45457931 | 45458528 | *LTP1* | AT2G15230 | Embryo Development |
| 606 | chrC04 | 46665589 | 46667650 | *EMB2776* | AT2G41500 | Embryo Sac Development |
| 607 | chrC04 | 47470777 | 47472984 | *ACS1* | AT2G43750 | Double Fertilization |
| 608 | chrC04 | 47471155 | 47472914 | *ATCS-C* | AT3G59760 | Double Fertilization |
| 609 | chrC04 | 47786339 | 47796330 | *CRM1B* | AT3G03110 | Embryo Sac Development |
| 610 | chrC04 | 48180341 | 48180917 | *EDA13* | AT2G47990 | Embryo Sac Development |
| 611 | chrC04 | 48241970 | 48243614 | *ARID1* | AT2G46040 | pollen development |
| 612 | chrC04 | 48377352 | 48377939 | *EDA4* | AT2G48140 | Embryo Sac Development |
| 613 | chrC05 | 16578 | 17585 | *HAP1* | AT1G02140 | Embryo Development |
| 614 | chrC05 | 37524 | 39342 | *SPL8* | AT1G02065 | Embryo Sac Development |
| 615 | chrC05 | 172258 | 173217 | *ATPRD3* | AT1G01690 | Embryo Sac Development |
| 616 | chrC05 | 279185 | 280237 | *BPS1* | AT1G01550 | Embryo Development |
| 617 | chrC05 | 354636 | 356302 | *CYP703* | AT1G01280 | pollen development |
| 618 | chrC05 | 435336 | 442475 | *ASU1* | AT1G01040 | Embryo Development |
| 619 | chrC05 | 435362 | 442475 | *ASU1* | AT1G01040 | Embryo Development |
| 620 | chrC05 | 720737 | 721468 | *TAF13* | AT1G02680 | Embryo Development |
| 621 | chrC05 | 741920 | 743187 | *EMB2386* | AT1G02780 | Embryo Development |
| 622 | chrC05 | 756941 | 757169 | *ATLEA3* | AT1G02820 | Embryo Development |
| 623 | chrC05 | 1044457 | 1048289 | *CACTIN* | AT1G03910 | Embryo Development |
| 624 | chrC05 | 1044601 | 1048289 | *CACTIN* | AT1G03910 | Embryo Development |
| 625 | chrC05 | 1282427 | 1283738 | *BDL* | AT1G04550 | Embryo Development |
| 626 | chrC05 | 1564256 | 1565417 | *EMB2394* | AT1G05190 | Embryo Development |
| 627 | chrC05 | 1725780 | 1729660 | *CVP2* | AT1G05470 | Embryo Development |
| 628 | chrC05 | 1753276 | 1754291 | *OBAP1A* | AT1G05510 | Embryo Development |
| 629 | chrC05 | 1873551 | 1873834 | *BT3* | AT1G05690 | Embryo Sac Development |
| 630 | chrC05 | 2700263 | 2701642 | *APX1* | AT1G07890 | Embryo Development |
| 631 | chrC05 | 2926006 | 2940893 | *ABO4* | AT1G08260 | Embryo Development |
| 632 | chrC05 | 3828320 | 3830705 | *GRP23* | AT1G10270 | Embryo Development |
| 633 | chrC05 | 4060407 | 4061513 | *ARR4* | AT1G10470 | Embryo Development |
| 634 | chrC05 | 4423289 | 4427444 | *ATWAPL1* | AT1G11060 | embryo development ending in seed dormancy |
| 635 | chrC05 | 4423563 | 4428289 | *ATWAPL2* | AT1G61030 | embryo development ending in seed dormancy |
| 636 | chrC05 | 4471525 | 4475423 | *SCM* | AT1G11130 | ovlue development |
| 637 | chrC05 | 4936242 | 4936991 | *DRN* | AT1G12980 | ovlue development |
| 638 | chrC05 | 5232412 | 5233804 | *ANAC007* | AT1G12260 | Embryo Development |
| 639 | chrC05 | 6086590 | 6088802 | *ATMYB124* | AT1G14350 | Embryo Sac Development |
| 640 | chrC05 | 6326351 | 6328808 | *TTA1* | AT1G14740 | ovlue development |
| 641 | chrC05 | 6829219 | 6832517 | *ATPWP2* | AT1G15440 | Embryo Sac Development |
| 642 | chrC05 | 6931231 | 6936431 | *TPL* | AT1G15750 | ovlue development |
| 643 | chrC05 | 7889536 | 7890588 | *HLL* | AT1G17560 | ovlue development |
| 644 | chrC05 | 8153210 | 8157147 | *ATNACK1* | AT1G18370 | Embryo Sac Development |
| 645 | chrC05 | 8325238 | 8326942 | *PSP* | AT1G18640 | ovlue development |
| 646 | chrC05 | 8884136 | 8886421 | *DA1* | AT1G19270 | ovlue development |
| 647 | chrC05 | 9259472 | 9263813 | *ARF5* | AT1G19850 | ovlue development |
| 648 | chrC05 | 10403043 | 10403679 | *EMB2170* | AT1G21390 | ovlue development |
| 649 | chrC05 | 10641717 | 10644658 | *SECA2* | AT1G21650 | ovlue development |
| 650 | chrC05 | 10687865 | 10692930 | *EMB1968* | AT1G21690 | ovlue development |
| 651 | chrC05 | 11373328 | 11377085 | *AGL104* | AT1G22130 | pollen development |
| 652 | chrC05 | 12428820 | 12430504 | *INO* | AT1G23420 | ovlue development |
| 653 | chrC05 | 12704649 | 12705946 | *ATHAP2B* | AT3G05690 | Double Fertilization |
| 654 | chrC05 | 12831037 | 12831377 | *ATLEC1* | AT1G21970 | embryo development ending in seed dormancy |
| 655 | chrC05 | 14208025 | 14212817 | *OVA9* | AT1G25350 | ovlue development |
| 656 | chrC05 | 14941299 | 14943526 | *SEPALLATA3* | AT1G24260 | ovlue development |
| 657 | chrC05 | 17674291 | 17677827 | *EMB2279* | AT1G30610 | embryo development ending in seed dormancy |
| 658 | chrC05 | 18730241 | 18732648 | *ATP8* | AT1G32230 | ovlue development |
| 659 | chrC05 | 18811667 | 18815509 | *KAN2* | AT1G32240 | ovlue development |
| 660 | chrC05 | 21531872 | 21534427 | *EMB1144* | AT1G48850 | ovlue development |
| 661 | chrC05 | 22159425 | 22160645 | *EDA11* | AT1G55420 | Embryo Sac Development |
| 662 | chrC05 | 26982758 | 27031152 | *ATMDN1* | AT1G67120 | Embryo Sac Development |
| 663 | chrC05 | 27218643 | 27219142 | *SAMBA* | AT1G32310 | pollen development |
| 664 | chrC05 | 27283213 | 27287255 | *KAN2* | AT1G32240 | ovlue development |
| 665 | chrC05 | 27412876 | 27415298 | *ATP8* | AT1G32230 | ovlue development |
| 666 | chrC05 | 30840295 | 30841319 | *AGC2-3* | AT1G51170 | ovlue development |
| 667 | chrC05 | 31924546 | 31929450 | *ATUBP14* | AT3G20630 | embryo development ending in seed dormancy |
| 668 | chrC05 | 33585526 | 33587014 | *ATMGT5* | AT4G28580 | pollen development |
| 669 | chrC05 | 36715076 | 36717843 | *MS1* | AT5G22260 | pollen development |
| 670 | chrC05 | 38233420 | 38236516 | *OVA5* | AT3G13490 | ovlue development |
| 671 | chrC05 | 39034536 | 39046390 | *RRP5* | AT3G11964 | Embryo Sac Development |
| 672 | chrC05 | 40517315 | 40520131 | *IPGAM2* | AT3G08590 | pollen development |
| 673 | chrC05 | 40517339 | 40520167 | *IPGAM1* | AT1G09780 | pollen development |
| 674 | chrC05 | 41319935 | 41321469 | *ATPPR2* | AT3G06430 | embryo development ending in seed dormancy |
| 675 | chrC05 | 41331531 | 41336754 | *CHR11* | AT3G06400 | Embryo Sac Development |
| 676 | chrC05 | 41797974 | 41800189 | *ATS6A.2* | AT3G05530 | Embryo Sac Development |
| 677 | chrC05 | 42769755 | 42771502 | *EDA5* | AT3G03650 | Embryo Sac Development |
| 678 | chrC06 | 1663692 | 1668501 | *ATMCM2* | AT1G44900 | ovlue development |
| 679 | chrC06 | 2525940 | 2527025 | *ATTAD2* | AT1G48175 | ovlue development |
| 680 | chrC06 | 5025262 | 5026356 | *AGC2-3* | AT1G51170 | ovlue development |
| 681 | chrC06 | 5221573 | 5223187 | *ASH2R* | AT1G51450 | embryo development ending in seed dormancy |
| 682 | chrC06 | 5886407 | 5890475 | *ATHB15* | AT1G52150 | ovlue development |
| 683 | chrC06 | 7850957 | 7860365 | *ATDEK1* | AT1G55350 | embryo development ending in seed dormancy |
| 684 | chrC06 | 9143017 | 9144964 | *EMB3003* | AT1G34430 | ovlue development |
| 685 | chrC06 | 9156271 | 9159491 | *EMB2756* | AT1G34550 | ovlue development |
| 686 | chrC06 | 15370571 | 15372667 | *ATCUL1* | AT4G02570 | embryo development ending in seed dormancy |
| 687 | chrC06 | 19706281 | 19709207 | *AGL5* | AT2G42830 | ovlue development |
| 688 | chrC06 | 20532058 | 20533925 | *RMF* | AT3G61730 | pollen development |
| 689 | chrC06 | 21519545 | 21524862 | *TPL* | AT1G15750 | ovlue development |
| 690 | chrC06 | 21919432 | 21924243 | *EMB1047* | AT1G79560 | embryo development ending in seed dormancy |
| 691 | chrC06 | 21919810 | 21922695 | *EMB1047* | AT1G79560 | embryo development ending in seed dormancy |
| 692 | chrC06 | 25616031 | 25618986 | *EDA17* | AT1G72970 | Embryo Sac Development |
| 693 | chrC06 | 26179279 | 26182986 | *PIN7* | AT1G23080 | embryo development ending in seed dormancy |
| 694 | chrC06 | 28224133 | 28225135 | *YAO* | AT4G05410 | Embryo Sac Development |
| 695 | chrC06 | 28536950 | 28539794 | *GLC* | AT1G65450 | Double Fertilization |
| 696 | chrC06 | 30085046 | 30085383 | *EDA32* | AT3G62210 | Embryo Sac Development |
| 697 | chrC06 | 30196742 | 30200817 | *POD1* | AT1G67960 | Double Fertilization |
| 698 | chrC06 | 30710516 | 30715464 | *MGP3* | AT1G68990 | embryo development ending in seed dormancy |
| 699 | chrC06 | 32873966 | 32877051 | *ATSERK1* | AT1G71830 | embryo development ending in seed dormancy |
| 700 | chrC06 | 32965395 | 32967278 | *HISN6A* | AT5G10330 | embryo development ending in seed dormancy |
| 701 | chrC06 | 33458358 | 33463153 | *EDA25* | AT1G72440 | Embryo Sac Development |
| 702 | chrC06 | 34621003 | 34621995 | *BZR1* | AT1G75080 | ovlue development |
| 703 | chrC06 | 35075430 | 35076923 | *EMB2719* | AT1G20200 | ovlue development |
| 704 | chrC06 | 35430869 | 35432630 | *ATSPP* | AT2G03120 | pollen development |
| 705 | chrC06 | 35772434 | 35779266 | *ASHH2* | AT1G77300 | Embryo Sac Development |
| 706 | chrC06 | 36027692 | 36031170 | *AGL66* | AT1G77980 | pollen development |
| 707 | chrC07 | 2194397 | 2195167 | *PLA2-BETA* | AT2G19690 | pollen development |
| 708 | chrC07 | 4484913 | 4486121 | *ARL2* | AT2G18390 | Embryo Sac Development |
| 709 | chrC07 | 9986575 | 9986981 | *GLC* | AT1G65450 | Double Fertilization |
| 710 | chrC07 | 15842709 | 15865922 | *ENP1* | AT1G31660 | Embryo Sac Development |
| 711 | chrC07 | 16059757 | 16063783 | *ATLEC2* | AT1G28300 | ovlue development |
| 712 | chrC07 | 16276076 | 16277071 | *ATTAD2* | AT1G48175 | ovlue development |
| 713 | chrC07 | 16918463 | 16921480 | *EMB2421* | AT1G24340 | embryo development ending in seed dormancy |
| 714 | chrC07 | 16990435 | 16992424 | *SEPALLATA3* | AT1G24260 | ovlue development |
| 715 | chrC07 | 18603603 | 18605241 | *INO* | AT1G23420 | ovlue development |
| 716 | chrC07 | 18901778 | 18904248 | *PIN7* | AT1G23080 | embryo development ending in seed dormancy |
| 717 | chrC07 | 19794635 | 19796619 | *AGL104* | AT1G22130 | pollen development |
| 718 | chrC07 | 19944413 | 19945050 | *ATLEC1* | AT1G21970 | embryo development ending in seed dormancy |
| 719 | chrC07 | 20975080 | 20975987 | *BIG3* | AT1G01960 | Embryo Sac Development |
| 720 | chrC07 | 20997732 | 21005667 | *EMB1507* | AT1G20960 | ovlue development |
| 721 | chrC07 | 21404687 | 21407031 | *EMB2719* | AT1G20200 | ovlue development |
| 722 | chrC07 | 25581271 | 25581834 | *HLL* | AT1G17560 | ovlue development |
| 723 | chrC07 | 27040619 | 27043792 | *ATPRD3* | AT1G01690 | Embryo Sac Development |
| 724 | chrC07 | 28470232 | 28471856 | *ATMYB124* | AT1G14350 | Embryo Sac Development |
| 725 | chrC07 | 28568241 | 28569066 | *EMB93* | AT2G03050 | embryo development ending in seed dormancy |
| 726 | chrC07 | 28601746 | 28603430 | *ATSPP* | AT2G03120 | pollen development |
| 727 | chrC07 | 29467966 | 29468356 | *BEL1* | AT5G41410 | ovlue development |
| 728 | chrC07 | 32316334 | 32318670 | *AACT2* | AT5G48230 | embryo development ending in seed dormancy |
| 729 | chrC07 | 32521464 | 32523277 | *XRI* | AT5G48720 | pollen development |
| 730 | chrC07 | 32640520 | 32647060 | *OVA2* | AT5G49030 | ovlue development |
| 731 | chrC07 | 33433812 | 33436728 | *EMB161* | AT5G27740 | embryo development ending in seed dormancy |
| 732 | chrC07 | 33542663 | 33543793 | *DPD1* | AT5G26940 | pollen development |
| 733 | chrC07 | 35316774 | 35317274 | *ANK6* | AT5G61230 | Embryo Sac Development |
| 734 | chrC07 | 37186398 | 37187733 | *ATRAB1C* | AT4G17530 | pollen development |
| 735 | chrC07 | 37186398 | 37187733 | *ATRAB1A* | AT5G47200 | pollen development |
| 736 | chrC07 | 38781438 | 38784873 | *ATML1* | AT4G21750 | embryo development ending in seed dormancy |
| 737 | chrC07 | 40703556 | 40706398 | *UNE17* | AT4G26330 | Double Fertilization |
| 738 | chrC07 | 41553873 | 41555243 | *ATMND1* | AT4G29170 | Embryo Sac Development |
| 739 | chrC07 | 42101765 | 42103383 | *ATS2* | AT4G30580 | embryo development ending in seed dormancy |
| 740 | chrC07 | 42223264 | 42224674 | *NFD1* | AT4G30930 | Embryo Sac Development |
| 741 | chrC07 | 43187290 | 43189411 | *EDA9* | AT4G34200 | Embryo Sac Development |
| 742 | chrC07 | 44180702 | 44185148 | *ACA7* | AT2G22950 | pollen development |
| 743 | chrC07 | 44311239 | 44313373 | *EDA40* | AT4G37890 | Embryo Sac Development |
| 744 | chrC07 | 44311239 | 44313052 | *EDA40* | AT4G37890 | Embryo Sac Development |
| 745 | chrC07 | 44578294 | 44578670 | *EC1.4* | AT4G39340 | Double Fertilization |
| 746 | chrC08 | 546458 | 547382 | *MEE4* | AT1G04630 | Embryo Development |
| 747 | chrC08 | 952355 | 956275 | *CVP2* | AT1G05470 | Embryo Development |
| 748 | chrC08 | 1114847 | 1116041 | *BT3* | AT1G05690 | Embryo Sac Development |
| 749 | chrC08 | 1768415 | 1769727 | *APX1* | AT1G07890 | Embryo Development |
| 750 | chrC08 | 1768415 | 1769314 | *APX1* | AT1G07890 | Embryo Development |
| 751 | chrC08 | 2433426 | 2433774 | *EMB1129* | AT1G49400 | ovlue development |
| 752 | chrC08 | 3001098 | 3004158 | *EMB1144* | AT1G48850 | ovlue development |
| 753 | chrC08 | 3878702 | 3879796 | *ATTAD2* | AT1G48175 | ovlue development |
| 754 | chrC08 | 8330861 | 8331462 | *ATMND1* | AT4G29170 | Embryo Sac Development |
| 755 | chrC08 | 8414238 | 8416218 | *AGL104* | AT1G22130 | pollen development |
| 756 | chrC08 | 8660029 | 8663372 | *PIN7* | AT1G23080 | embryo development ending in seed dormancy |
| 757 | chrC08 | 8828184 | 8828782 | *YAO* | AT4G05410 | Embryo Sac Development |
| 758 | chrC08 | 9228823 | 9229442 | *RPL21C* | AT1G35680 | ovlue development |
| 759 | chrC08 | 9499659 | 9507214 | *EMB2756* | AT1G34550 | ovlue development |
| 760 | chrC08 | 9928156 | 9929293 | *ATCYP5* | AT1G11680 | Embryo Development |
| 761 | chrC08 | 11262704 | 11272844 | *KAN2* | AT1G32240 | ovlue development |
| 762 | chrC08 | 12426016 | 12428107 | *EDA38* | AT4G14040 | Embryo Sac Development |
| 763 | chrC08 | 13171339 | 13172825 | *UNE16* | AT4G13640 | Double Fertilization |
| 764 | chrC08 | 18611748 | 18614520 | *DIG6* | AT1G08410 | Embryo Development |
| 765 | chrC08 | 18723370 | 18726386 | *ATPXL1* | AT1G08590 | Embryo Development |
| 766 | chrC08 | 18862460 | 18865059 | *IPGAM1* | AT1G09780 | pollen development |
| 767 | chrC08 | 18862498 | 18865069 | *IPGAM2* | AT3G08590 | pollen development |
| 768 | chrC08 | 19030441 | 19032794 | *GRP23* | AT1G10270 | Embryo Development |
| 769 | chrC08 | 19116639 | 19117741 | *ARR4* | AT1G10470 | Embryo Development |
| 770 | chrC08 | 19149714 | 19152734 | *EMB2004* | AT1G10510 | Embryo Development |
| 771 | chrC08 | 19243786 | 19245941 | *ACLA-1* | AT1G10670 | Embryo Development |
| 772 | chrC08 | 19860269 | 19861634 | *ANAC007* | AT1G12260 | Embryo Development |
| 773 | chrC08 | 20093878 | 20095524 | *EMB1586* | AT1G12770 | Embryo Development |
| 774 | chrC08 | 20097553 | 20098956 | *UGE3* | AT1G63180 | pollen development |
| 775 | chrC08 | 20423233 | 20425425 | *TTA1* | AT1G14740 | ovlue development |
| 776 | chrC08 | 20453743 | 20457358 | *ADL1C* | AT1G14830 | pollen development |
| 777 | chrC08 | 20612412 | 20615543 | *ATPWP2* | AT1G15440 | Embryo Sac Development |
| 778 | chrC08 | 21457787 | 21459324 | *PSP* | AT1G18640 | ovlue development |
| 779 | chrC08 | 21523567 | 21525671 | *AGL65* | AT1G18750 | pollen development |
| 780 | chrC08 | 21766289 | 21768188 | *DA1* | AT1G19270 | ovlue development |
| 781 | chrC08 | 21799064 | 21800167 | *BZR1* | AT1G75080 | ovlue development |
| 782 | chrC08 | 22023102 | 22026522 | *ARF5* | AT1G19850 | ovlue development |
| 783 | chrC08 | 22712577 | 22719401 | *SECA2* | AT1G21650 | ovlue development |
| 784 | chrC08 | 22824610 | 22825028 | *ATLEC1* | AT1G21970 | embryo development ending in seed dormancy |
| 785 | chrC08 | 27138418 | 27141314 | *FBL17* | AT3G54650 | pollen development |
| 786 | chrC08 | 27683500 | 27686377 | *OVA1* | AT3G55400 | ovlue development |
| 787 | chrC08 | 28750243 | 28754086 | *EDA7* | AT3G56990 | Embryo Sac Development |
| 788 | chrC08 | 29346471 | 29346867 | *GIG1* | AT3G57860 | Embryo Sac Development |
| 789 | chrC08 | 29930899 | 29933827 | *ATSYN3* | AT3G59550 | pollen development |
| 790 | chrC08 | 30298126 | 30299517 | *EDA14* | AT3G60360 | Embryo Sac Development |
| 791 | chrC08 | 31973486 | 31981768 | *PKL* | AT2G25170 | Embryo Development |
| 792 | chrC08 | 32117965 | 32118686 | *AGL61* | AT2G24840 | Embryo Sac Development |
| 793 | chrC08 | 32332744 | 32335100 | *LAP1* | AT2G24200 | Double Fertilization |
| 794 | chrC08 | 32755300 | 32759531 | *ACA7* | AT2G22950 | pollen development |
| 795 | chrC08 | 33337844 | 33338154 | *EC1.2* | AT2G21740 | Double Fertilization |
| 796 | chrC08 | 33337844 | 33338160 | *EC1.3* | AT2G21750 | Double Fertilization |
| 797 | chrC08 | 34172012 | 34175859 | *ATNACK1* | AT1G18370 | Embryo Sac Development |
| 798 | chrC08 | 34727347 | 34729322 | *ATRH36* | AT1G16280 | Embryo Sac Development |
| 799 | chrC08 | 34826415 | 34832089 | *TPL* | AT1G15750 | ovlue development |
| 800 | chrC08 | 35003071 | 35003494 | *HOS9* | AT2G01500 | Embryo Sac Development |
| 801 | chrC08 | 35063472 | 35066926 | *ADL1C* | AT1G14830 | pollen development |
| 802 | chrC08 | 35280066 | 35280874 | *ATMYB124* | AT1G14350 | Embryo Sac Development |
| 803 | chrC08 | 35740250 | 35743425 | *ATGLE1* | AT1G13120 | embryo development ending in seed dormancy |
| 804 | chrC08 | 35820928 | 35821547 | *DRN* | AT1G12980 | ovlue development |
| 805 | chrC08 | 36046175 | 36047546 | *ANAC007* | AT1G12260 | Embryo Development |
| 806 | chrC08 | 36230606 | 36233353 | *OVA7* | AT1G11870 | ovlue development |
| 807 | chrC08 | 36481912 | 36485687 | *SCM* | AT1G11130 | ovlue development |
| 808 | chrC08 | 36495076 | 36499358 | *ATWAPL1* | AT1G11060 | embryo development ending in seed dormancy |
| 809 | chrC08 | 36495426 | 36498398 | *ATWAPL2* | AT1G61030 | embryo development ending in seed dormancy |
| 810 | chrC08 | 36899100 | 36902223 | *PIAL1* | AT1G08910 | Embryo Development |
| 811 | chrC08 | 36911900 | 36920239 | *EMB2411* | AT1G08840 | Embryo Development |
| 812 | chrC08 | 37186481 | 37188148 | *APX1* | AT1G07890 | Embryo Development |
| 813 | chrC08 | 37232864 | 37235914 | *CACTIN* | AT1G03910 | Embryo Development |
| 814 | chrC08 | 37497209 | 37497547 | *EMB2394* | AT1G05190 | Embryo Development |
| 815 | chrC08 | 37788924 | 37792344 | *CLO* | AT1G06220 | Embryo Sac Development |
| 816 | chrC08 | 38349750 | 38350812 | *EMB2386* | AT1G02780 | Embryo Development |
| 817 | chrC09 | 4419 | 8291 | *ATCUL1* | AT4G02570 | embryo development ending in seed dormancy |
| 818 | chrC09 | 11584 | 13393 | *UNE12* | AT4G02590 | Double Fertilization |
| 819 | chrC09 | 524892 | 525872 | *EDA35* | AT4G05440 | Embryo Sac Development |
| 820 | chrC09 | 1304165 | 1306134 | *OBE2* | AT5G48160 | embryo development ending in seed dormancy |
| 821 | chrC09 | 1313855 | 1316227 | *AACT2* | AT5G48230 | embryo development ending in seed dormancy |
| 822 | chrC09 | 1429428 | 1430639 | *XRI* | AT5G48720 | pollen development |
| 823 | chrC09 | 2859983 | 2861956 | *ABS* | AT5G23260 | ovlue development |
| 824 | chrC09 | 3059461 | 3059775 | *ANK6* | AT5G61230 | Embryo Sac Development |
| 825 | chrC09 | 3986844 | 3987064 | *EC1.5* | AT5G64720 | Double Fertilization |
| 826 | chrC09 | 5209888 | 5211438 | *AKRP* | AT5G66055 | embryo development ending in seed dormancy |
| 827 | chrC09 | 5993588 | 5995942 | *EMB2762* | AT2G17250 | embryo development ending in seed dormancy |
| 828 | chrC09 | 6632097 | 6632495 | *PLA2-BETA* | AT2G19690 | pollen development |
| 829 | chrC09 | 8258483 | 8260386 | *SPO11-2SPO11-2* | AT1G63990 | Embryo Sac Development |
| 830 | chrC09 | 9267117 | 9270642 | *EMB71* | AT1G63700 | embryo development ending in seed dormancy |
| 831 | chrC09 | 11177344 | 11179649 | *ACLA-1* | AT1G10670 | Embryo Development |
| 832 | chrC09 | 11438314 | 11439223 | *ARR4* | AT1G10470 | Embryo Development |
| 833 | chrC09 | 13636290 | 13643588 | *SPP* | AT5G42390 | embryo development ending in seed dormancy |
| 834 | chrC09 | 15523791 | 15525263 | *MOB1-LIKE* | AT5G45550 | Embryo Sac Development |
| 835 | chrC09 | 16040554 | 16040900 | *HLL* | AT1G17560 | ovlue development |
| 836 | chrC09 | 18462900 | 18464577 | *ATSPP* | AT2G03120 | pollen development |
| 837 | chrC09 | 19006017 | 19007821 | *YAO* | AT4G05410 | Embryo Sac Development |
| 838 | chrC09 | 30998361 | 31001106 | *OVA6* | AT5G52520 | ovlue development |
| 839 | chrC09 | 33064140 | 33066857 | *EMB2737* | AT5G53860 | embryo development ending in seed dormancy |
| 840 | chrC09 | 33763173 | 33763566 | *BEL1* | AT5G41410 | ovlue development |
| 841 | chrC09 | 34280846 | 34282503 | *ATGPT1* | AT5G54800 | Embryo Sac Development |
| 842 | chrC09 | 35152926 | 35154930 | *KAN* | AT5G16560 | ovlue development |
| 843 | chrC09 | 36922324 | 36924117 | *ATMSI1* | AT5G58230 | embryo development ending in seed dormancy |
| 844 | chrC09 | 38664001 | 38666170 | *ATRPP30* | AT5G59980 | Embryo Sac Development |
| 845 | chrC09 | 39940151 | 39941788 | *RHF2A* | AT5G22000 | Embryo Sac Development |
| 846 | chrC09 | 40119214 | 40124694 | *AGO9* | AT5G21150 | Embryo Sac Development |
| 847 | chrC09 | 41089604 | 41093837 | *ATOEP80* | AT5G19620 | embryo development ending in seed dormancy |
| 848 | chrC09 | 41842648 | 41847517 | *CHR11* | AT3G06400 | Embryo Sac Development |
| 849 | chrC09 | 42889476 | 42890337 | *UNE6* | AT3G03340 | Double Fertilization |
| 850 | chrC09 | 43141705 | 43149363 | *CRM1B* | AT3G03110 | Embryo Sac Development |
| 851 | chrC09 | 43219201 | 43223057 | *DOT2* | AT5G16780 | embryo development ending in seed dormancy |
| 852 | chrC09 | 43231612 | 43236116 | *TOZ* | AT5G16750 | embryo development ending in seed dormancy |
| 853 | chrC09 | 43784549 | 43786920 | *AGL2* | AT5G15800 | ovlue development |
| 854 | chrC09 | 44842167 | 44846314 | *NEF1* | AT5G13390 | pollen development |
| 855 | chrC09 | 48454004 | 48455256 | *CYP77A7* | AT3G10560 | Double Fertilization |

# Table S5. Information of 80 pairs primers evenly distributed on the 19 linkage groups in *B. napus* used for genome background estimation.

| Linkage groups | Primers |
| --- | --- |
| A01 | BrBAC001 |
| A01 | BrBAC019 |
| A02 | BrBAC031 |
| A02 | BrBAC051 |
| A02 | BrSF000037-20 |
| A02 | BrSF000037-64 |
| A02 | BrSF000043-57 |
| A02 | CB10628 |
| A02 | cnu046 |
| A02 | sR6293 |
| A03 | BnEMS0894 |
| A03 | BoSF1271 |
| A03 | BoSF1276 |
| A03 | BrBAC092 |
| A03 | BrGMS2766 |
| A03 | BrSF000211-2 |
| A03 | Na14G10 |
| A03 | sN11722 |
| A03 | BoGMS0707 |
| A03 | BrBAC090 |
| A04 | BoSF1161 |
| A05 | BoSF1230 |
| A05 | BrSF000017-169 |
| A05 | BrSF000020-125 |
| A05 | BrSF000030-103 |
| A05 | Na14G02 |
| A06 | BoGMS0314 |
| A06 | BrBAC149 |
| A06 | BrSF000042-5 |
| A06 | BrSF46-291 |
| A07 | cnu037 |
| A08 | BnEMS0236 |
| A08 | BnEMS1142 |
| A08 | BrBAC210 |
| A09 | BnEMS0337 |
| A09 | BnSF2342-39 |
| A09 | BrBAC250 |
| A09 | BrBAC251 |
| A09 | BrSF000007-101 |
| A09 | ni60 |
| A09 | P525 |
| A09 | BnSF400-237 |
| A10 | niab103 |
| A10 | sS2066 |
| C01 | BoSF0442 |
| C01 | BoSF1270 |
| C01 | BoSF1372 |
| C01 | CB10277 |
| C01 | CB10369 |
| C02 | BnEMS0959 |
| C02 | BoGMS0665 |
| C02 | BoSF0284 |
| C02 | BoSF0757 |
| C02 | CB10026 |
| C03 | BoGMS0678 |
| C03 | BoGMS0819 |
| C03 | P068 |
| C04 | BoGMS0282 |
| C04 | BoGMS1219 |
| C04 | BoGMS2091 |
| C04 | BoSF0318 |
| C04 | BoSF0997 |
| C04 | BoSF1492 |
| C05 | BoSF1600 |
| C05 | BrBAC040 |
| C05 | CB10487 |
| C05 | sS2131 |
| C06 | CB10534 |
| C07 | BoSF1144 |
| C07 | BoSF1468 |
| C08 | BnEMS0020 |
| C08 | BnEMS0606 |
| C08 | BnGMS0509 |
| C08 | BoSF0365 |
| C08 | BoSF1222 |
| C08 | BrGMS0025 |
| C08 | CB10053 |
| C08 | CB10179 |
| C09 | BoSF0980 |
| C09 | SF36885 |

# Supplementary figures


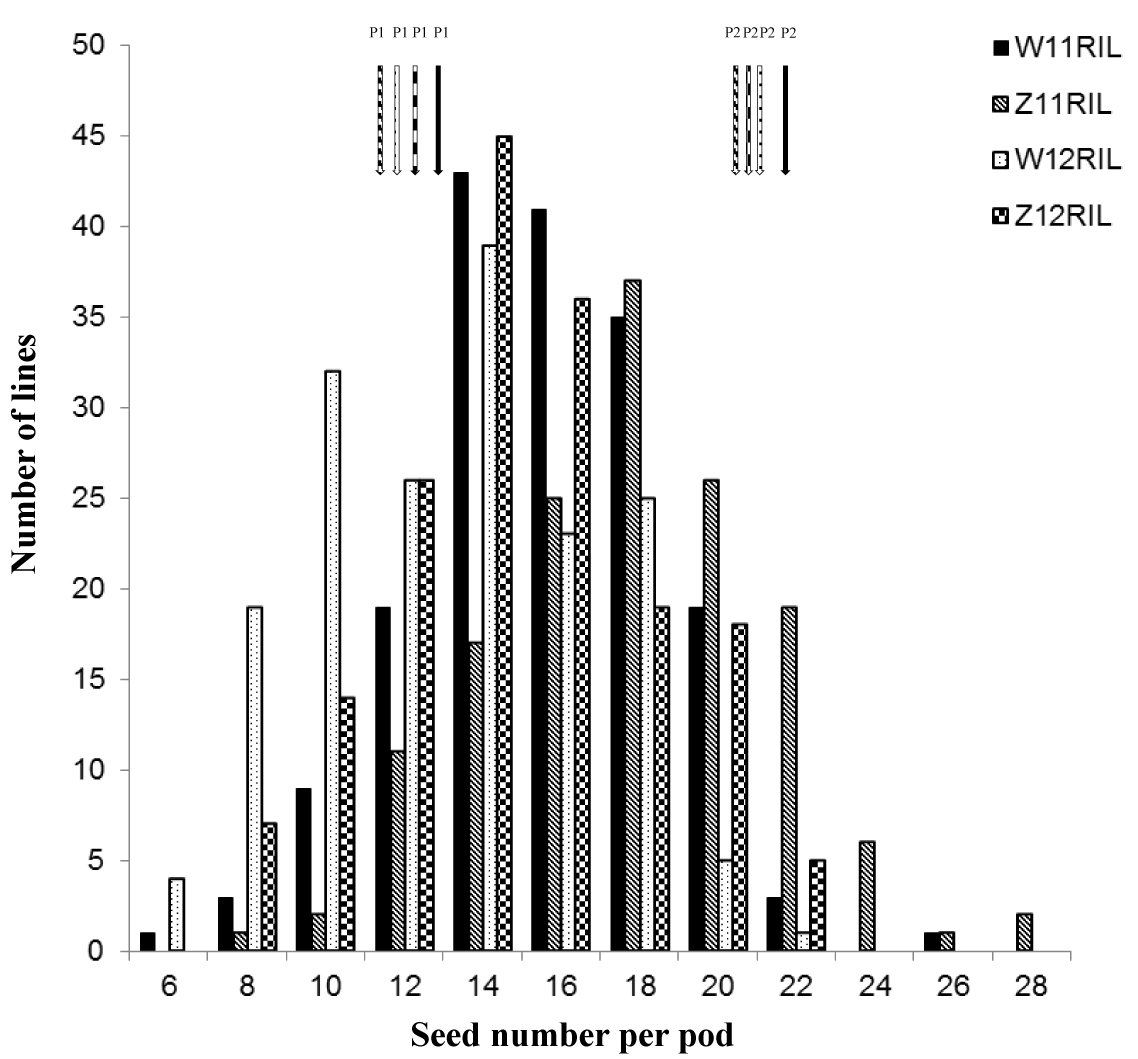


**Figure S1. Distribution of SNPP in the RIL population planted in four environments.** The horizontal axis represents the trait value of SNPP. The vertical axis represents the number of lines within the population. The different experiments are represented by different column types. P1: Zhongshuang11, P2: No. 73290.


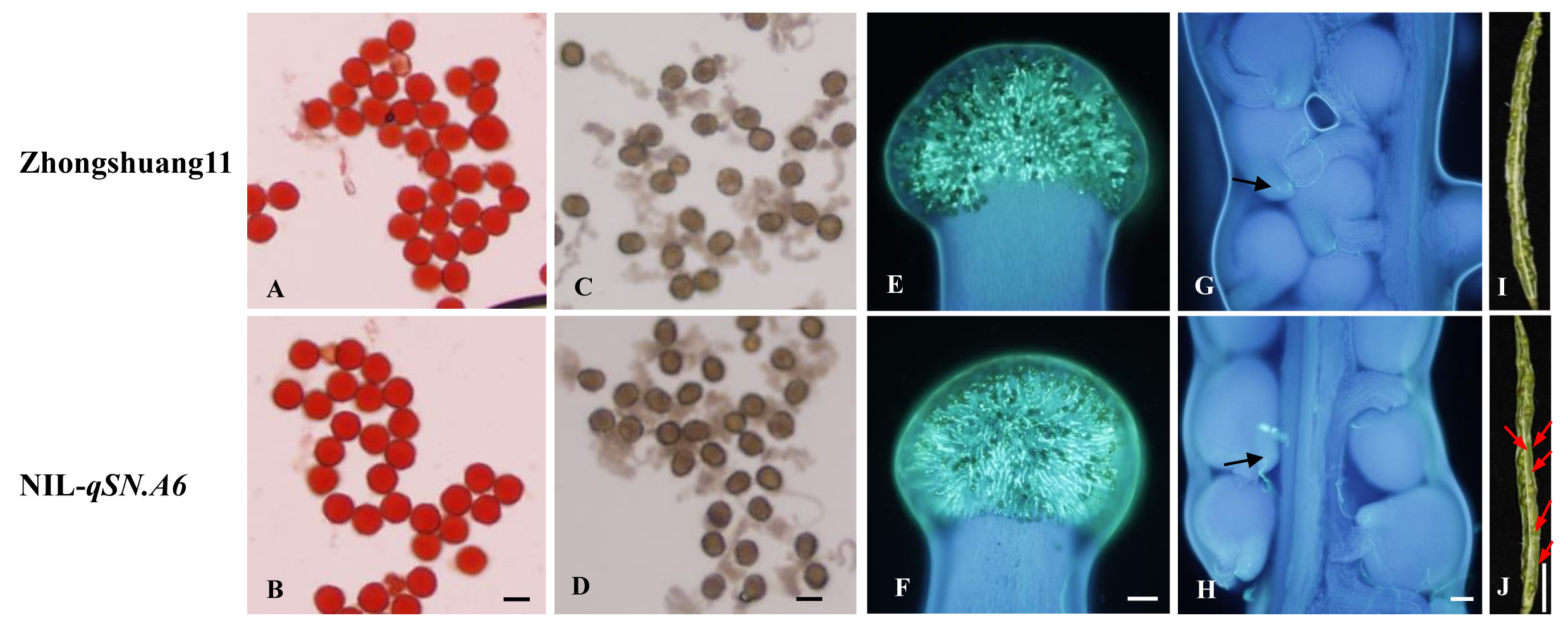


**Figure S2. Cytological observation of pollen viability, pollen germination (in vitro and in vivo), and pollen tube growth.** Characterization of the pollen viability of Zhongshuang11 and NIL-*qSN.A6* by 1% acetocarmine staining. Pollen of both lines appears to be fertile (A and B). Pollen germination in vitro of both Zhongshuang11 and NIL-*qSN.A6* is normal (C and D). Pollen grains germinate on the stigma of Zhongshuang11 (E) and NIL-*qSN.A6* (F) at 3DBF, the development of the pollen tube appears normal, and the pollen tubes have passed into the micropyle (arrow in G) of the ovule of Zhongshuang11 and NIL-*qSN.A6* (arrow in H), which also appear normal. (I, J) Pods of Zhongshuang11 and NIL-*qSN.A6*; arrowheads indicate sterility. *Bar* 10 μm (A, B, C, D), 100 μm (E, F, G, H), 1 cm (I, J).


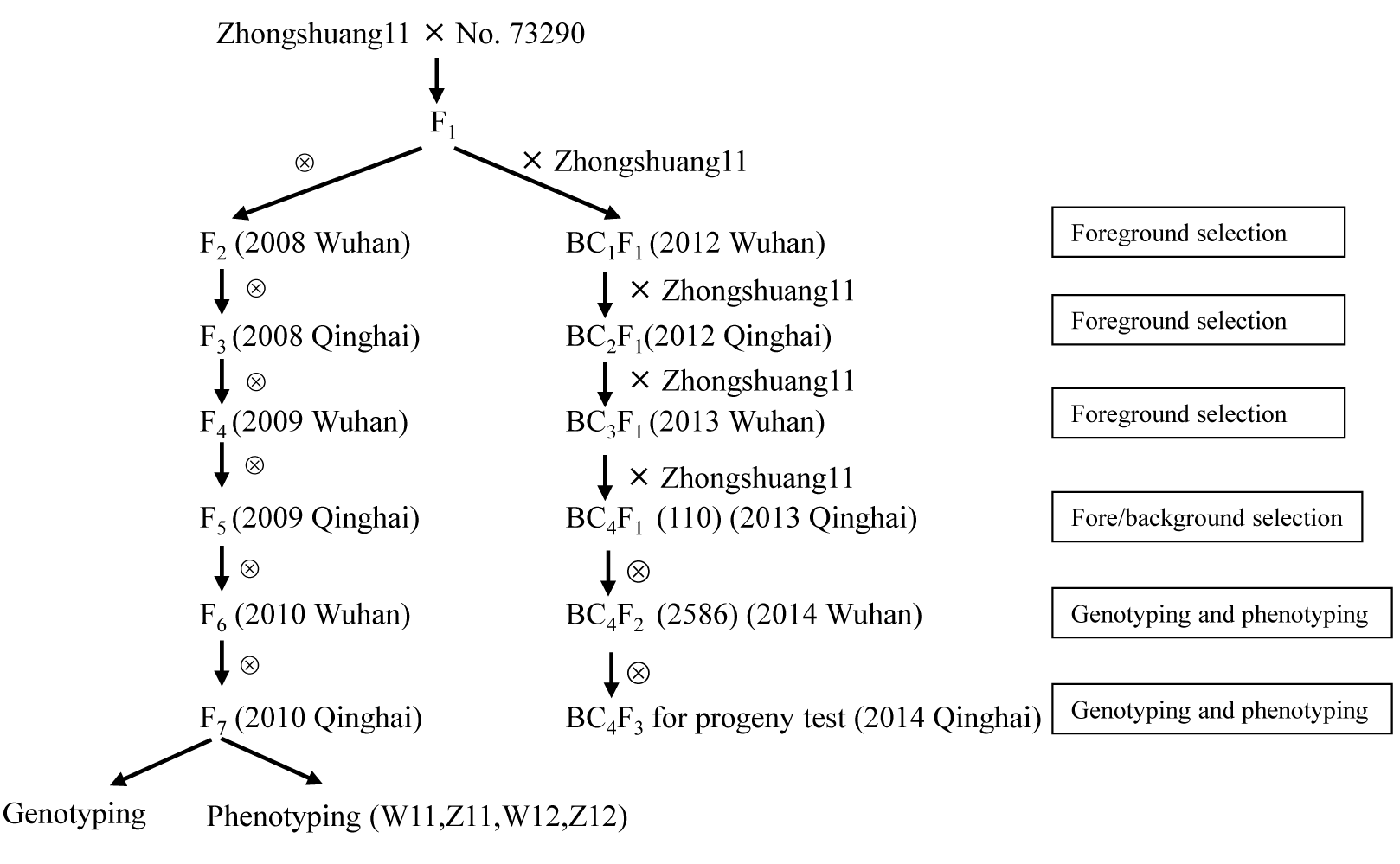


**Figure S3. Procedure for the development of RIL (left) and NIL (right) populations.** The descriptions in the parentheses following each generation of materials give the year and location.
